# Glycogen synthase kinase-3 inhibition and insulin enhance proliferation and inhibit maturation of human iPSC-derived cardiomyocytes via TCF and FOXO signaling

**DOI:** 10.1016/j.stemcr.2024.11.001

**Published:** 2024-12-05

**Authors:** Qianliang Yuan, Devin Verbueken, Rafeeh Dinani, Rosa Kim, Eric Schoger, Chloé D. Morsink, Shamim Amiri Simkooei, Luuk J.M. Kemna, Jesper Hjortnaes, Diederik W.D. Kuster, Reinier A. Boon, Laura Cecilia Zelarayan, Jolanda van der Velden, Jan W. Buikema

**Affiliations:** 1 Amsterdam Cardiovascular Sciences, Department of Physiology, Amsterdam University Medical Center, VU University, Amsterdam, the Netherlands; 2 Amsterdam Heart Center, Department of Cardiology, Amsterdam University Medical Center, Amsterdam, the Netherlands; 3 DZHK (German Centre for Cardiovascular Research) Partner Site Göttingen, Göttingen, Germany; 4 Institute of Pharmacology and Toxicology, University Medical Center Göttingen, Göttingen, Germany; 5 Laboratory of Experimental Cardiology, Department of Cardiology, Leiden University Medical Center, Leiden, the Netherlands; 6 Heart Lung Center, Department of Cardiothoracic Surgery, Leiden University Medical Center, Leiden, the Netherlands; 7 Justus Liebig University, Medical Clinic I, Department of Cardiology and Angiology, Giessen, Germany

**Keywords:** hiPSC-CMs, iPSC, cardiomyocytes, CHIR99021, Wnt, insulin, proliferation, maturation, FOXO, TCF

## Abstract

Embryonic signaling pathways exert stage-specific effects during cardiac development, yet the precise signals for proliferation or maturation remain elusive. To uncover the cues for proliferation, we performed a combinatory cell-cycle screen for insulin and glycogen synthase kinase-3 (GSK3) inhibition in spontaneously beating human induced pluripotent stem cell-derived cardiomyocytes (hiPSC-CMs). Our analysis for proliferation, and subsequential downstream sarcomere development, gene expression analysis, and molecular interventions identified a temporal interplay between insulin/Akt/FOXO and CHIR99021/Wnt/GSK3/TCF signaling. Combined pathway activation led to proliferation of immature hiPSC-CMs with low sarcomere and mitochondria content, while, in the absence of pathway activators, cardiomyocytes rapidly exited the cell cycle and fetched higher organization of sarcomeres and mitochondria. Our data demonstrate two important pathways, which enhance proliferation and inhibit maturation, and provide molecular mechanistic understanding of these cell fate decisions in immature hiPSC-CMs.

## Highlights

• Insulin and GSK3β inhibition are two important transient cues for proliferation of immature hiPSC-CMs and inhibit maturation
• GSK3β inhibition represses sarcomere buildup in immature hiPSC-CMs
• Inhibition of FOXO transcription underlies cell-cycle activity in response to insulin

## Graphical abstract

**Figure:**
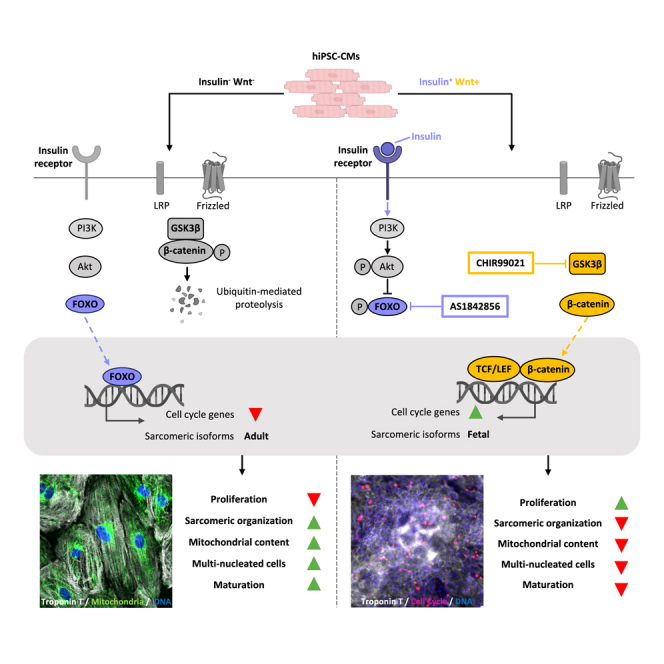

## Teaser

Understanding of the molecular cues that drive hiPSC-cardiomyocyte proliferation or maturation would be beneficial for tissue engineering and regenerative approaches for heart failure. Buikema and colleagues demonstrate that insulin and glycogen synthase kinase-3 inhibition concomitantly promote proliferation while inhibiting maturation via FOXO and TCF signaling.

## Introduction

The lack of understanding about true regeneration of the human heart is reflected in the current medical therapies for heart failure that do not aim to cure the underlying deficit of functional cardiomyocytes (Galdos et al., 2017; Lazar et al., 2017; Ouwerkerk et al., 2023). Whereas cell turnover in the adult heart is extremely low, during development embryonic signaling pathways regulate the robust increase in myocardial mass through cardiomyocyte duplication. Orchestration of these cell fate decisions require temporal and stage-specific specification and growth of the various myocardial compartments. This involves a tight regulation of the local cardiomyocyte proliferation and is directed by a network of signaling pathways, including Neuregulin1/ErbB2/4 (Bersell et al., 2009; D'Uva et al., 2015; Grego-Bessa et al., 2023), bone morphogenic proteins (BMPs) (Klaus et al., 2007), insulin growth factor (IGF)/phosphatidylinositol 3-kinase (PI3K)-AKT (Engels et al., 2014), Hippo-YAP (Heallen et al., 2011; Lin et al., 2015; von Gise et al., 2012; Xin et al., 2013), and NOTCH (Collesi et al., 2008; Grego-Bessa et al., 2007).

Remarkably, within the developing ventricular compartment, differential proliferation rates are observed within the compacted, trabecular, and septal zones. The proliferation rates of immature cardiomyocytes in the compacted myocardium gradually decline following specification and maturation (Buikema et al., 2013; Ye et al., 2015). Differentiation coincides with multiple isoform switches of genes that regulate the expression of proteins essential for the contractile apparatus, culminating in their functional maturity (Miyata et al., 2000; Reiser et al., 2001), In parallel, these sarcomeric isotype switches coincide with a metabolic preference for fatty acids as the substrate for adenosine triphosphate (ATP) production (Lopaschuk et al., 1991). These functional and metabolic transitions in ventricular cardiomyocytes are driven by a demand for increased cardiac output of the growing body.

We and others have previously shown that during mitosis cardiomyocytes disassemble all sarcomeres before proceeding to cytokinesis, a phenomenon inversely related to contractility (Buikema et al., 2020; Yuan et al., 2022). Moreover, we and others have shown that the canonical Wnt/β-catenin signaling pathway plays an essential role in regulating the cell fate decisions such as required for cardiogenesis and cardiac development. Notably, the canonical Wnt/β-catenin signaling pathway exerts a biphasic effect on mesoderm development versus cardiac specification and growth, which is incorporated in the robust cardiac differentiation of human induced pluripotent stem cells (hiPSCs) (Lian et al., 2012, 2013) and proliferation of immature hiPSC-derived cardiomyocytes (hiPSC-CMs) (Buikema et al., 2020; Mills et al., 2019; Sharma et al., 2018; Titmarsh et al., 2016; Uosaki et al., 2013).

Yet, it remains unknown what cues facilitate the switch between proliferation and maturation in immature hiPSC-CMs (Fetterman et al., 2024). Here, we study the contribution of the CHIR99021 (CHIR) small molecule via the Wnt/glycogen synthase kinase (GSK)3β/β-catenin/T-cell factor (TCF) axis and insulin via the insulin/PI3K-AKT/Forkhead box O (FOXO) route on the stage-specific effects for proliferation and maturation in immature hiPSC-CMs. In a combinatory screen we found that both insulin and CHIR had effects on promoting proliferation of immature hiPSC-CMs, whereas the absence of these pathway activators led to rapid cell-cycle exit and metabolic and structural maturation of hiPSC-CMs. These findings provide novel insights into the molecular signaling pathways that control the interplay between proliferation and maturation of cardiomyocytes during heart development.

## Results

### Insulin and CHIR99021 are important cues for proliferation of immature hiPSC-CMs

**Figure 1:**
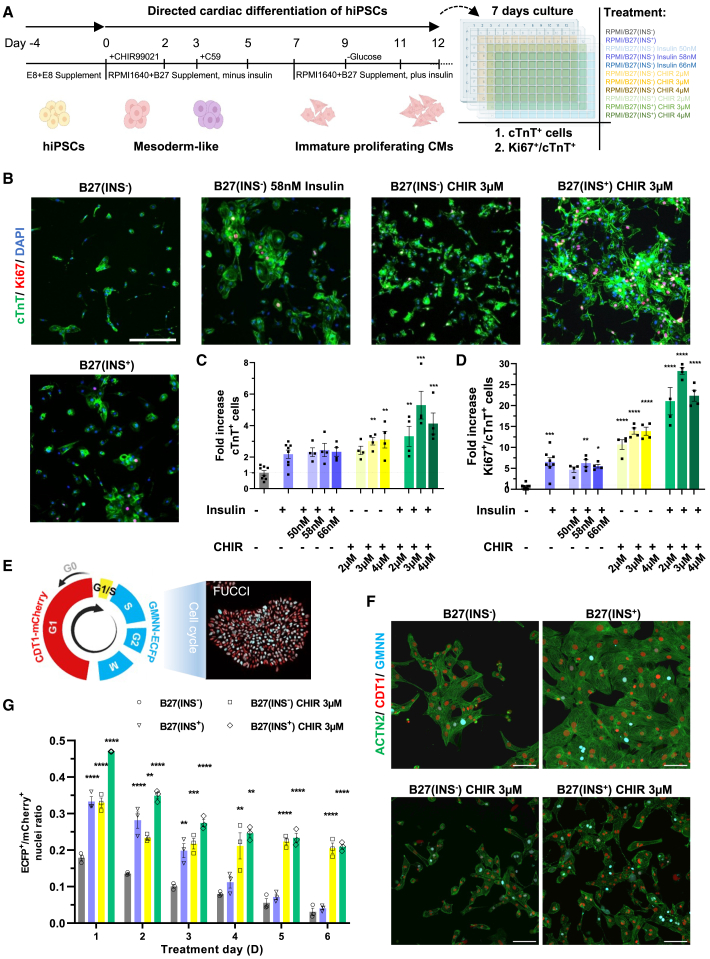
**Combinatorial screen of insulin and GSK3β inhibitorCHIR99021for hiPSC-CM proliferation** (A) Schematic of combinatory screen setup for insulin and CHIR99021 (CHIR) in hiPSC-CMs. (B) Representative immunofluorescence images of hiPSC-CMs for the optimized concentrations of insulin (58 nM–340 ng/mL) and/or CHIR (3.0 μM). Cells were stained for cTnT (green), Ki67 (red), and nuclei (DAPI, blue). Scale bar, 200 μm. (C) Graph displaying fold increase in cTnT^+^ cells normalized to B27(INS^−^). (D) Graph displaying fold increase in percentage of Ki67^+^/cTnT+ cells normalized to B27(INS^−^). (E) Schematic of FUCCI cell-cycle reporter system with CDT1-mCherry and GMNN-ECFP. (F) Representative triple fluorescent live-cell imaging hiPSC-CMs expressed CDT1-mCherry, GMNN-eCFP, and ACTN2-eGFP for indicated culture conditions. Scale bar, 100 μm. (G) Graph displaying ratios of CDT1-mCherry-positive cells (nuclei) and GMNN-ECFP-positive cells (nuclei). (B–D) Data are presented as mean ± SEM. *n* ≥ 4 independent cultures from three batches of SCVI-273 cell line. Data are normalized to B27(INS^−^) condition for fold increase of cell number in cTnT^+^ and/or Ki67^+^ hiPSC-CMs. (F and G) *N* = 3 independent experiments from the TC1133-ACTN2-Citrine-FUCCI cell line were analyzed in 9 replicates per condition. (C, D, and G) Statistical analysis by one-way ANOVA followed by Dunn’s multiple comparisons test compared to the B27(INS^−^) condition (. ^∗∗^*p* < 0.01, ^∗∗∗^*p* < 0.001, and ^∗∗∗∗^*p* < 0.0001.

One of the widely used methods for hiPSC-CM differentiation and culture consists of RPMI 1640 medium supplemented with B27 components containing an estimated final concentration of 58 nM (340 ng/mL) of insulin (Lian et al., 2012; Decourtye et al., 2018). Previous research demonstrated that the addition of CHIR in 2–5 μM to the culture media resulted in significant long-term expansion of hiPSC-CMs (Buikema et al., 2020; Quaife-Ryan et al., 2020). Remarkably, addition of CHIR to the chemically defined media consisting of three components (CDM3) did not result in a long-lasting expansion of hiPSC-CMs (data not shown). We set up a combinatory screen for a gradient of insulin and CHIR concentrations to assess the effects on hiPSC-CM proliferation (Figures 1A and S1). Our screen showed that the combination of 58 nM (340 ng/mL) insulin and 3.0 μM CHIR resulted in optimal proliferation of hiPSC-CMs (Figures 1B and S1D). Moreover, when both insulin and CHIR compounds were withdrawn from the RPMI 1640 with B27 supplement (minus insulin) media, the hiPSC-CMs that were sparsely seeded exhibited extremely low proliferation rates (1.48% ± 0.18%) versus high average proliferation rates (38.45% ± 3.86%) in cells stimulated with both insulin and CHIR, such as quantified by the fraction of Ki67- and Troponin T-positive cells (Figures 1C and 1D). In contrast, stimulating hiPSC-CMs with insulin or CHIR alone only increased proliferation by 5- to 10-fold (Figure 1D). We further confirmed hiPSC-CM cell-cycle activity upon CHIR and insulin exposure in a double-transgenic FUCCI hiPSC cell cycle reporter line (Figure 1E). Culturing cells in CHIR-containing media increased the percentage of S/G2-phase cardiomyocytes (ECFP^+^ cells), and supplementation of insulin enhanced the cell-cycle activation in the first days of treatment (Figures 1F, 1G, and S1E). We conclude that in our previously described expansion media, consisting of multiple factors (B27) and CHIR, the insulin and CHIR form the two essential molecular cues for hiPSC-CM proliferation.

### No exposure to insulin and CHIR99021 results in differentiation of immature hiPSC-CMs

**Figure 2:**
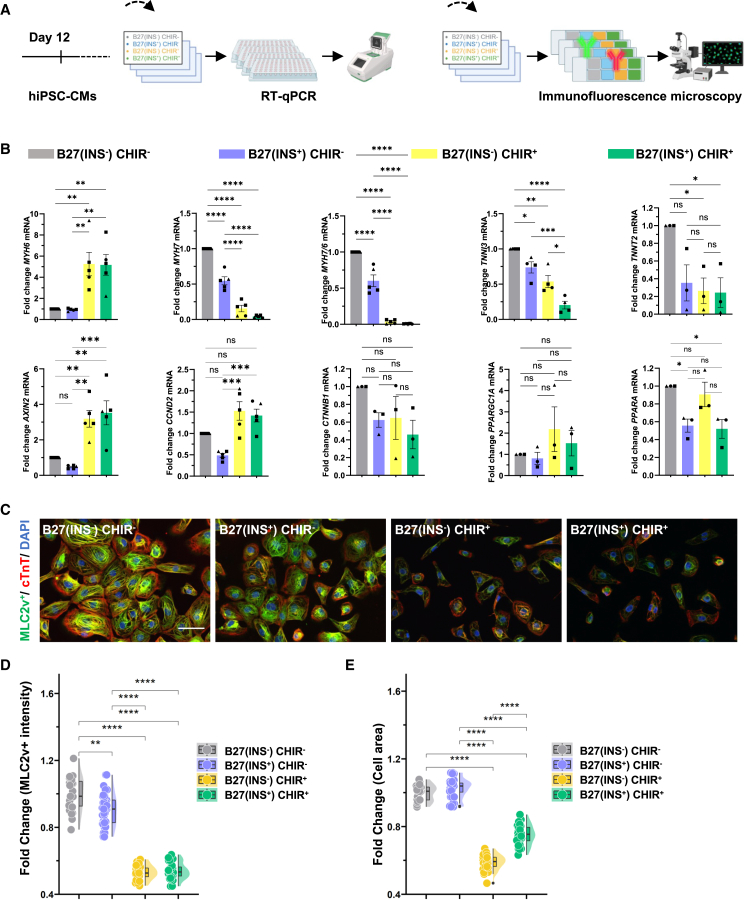
**Molecular characterization of immature hiPSC-CMs exposed to combinations of insulin and CHIR99021** (A) Schematic of the experimental setup (B) RT-qPCR analysis of mRNA expression of the indicated sarcomere, cell cycle, Wnt target, and glucose homeostasis genes. Data from each independent biological experiment are normalized to B27(INS^−^) CHIR^−^, as indicated by the dots: ▲ = SCVI-111 ● = SCVI-114 ▪ = SCVI-273. (C) Representative immunofluorescence images of hiPSC-CMs for the indicated treatments. Images show cTnT (red), MLC2v (green), and nuclei (DAPI, blue). Scale bar, 50 μm. (D and E) Quantification of the MLC2v^+^ intensity (D) and cell surface area; cTnT signal was thresholded and binarized, and holes were filled, whereafter the area per cell was measured (E) in 3 independent batches of hiPSC-CMs of the ● = SCVI-114 line. (B) Data are presented as mean ± SEM. Significance is determined by one-way ANOVA followed by Dunn’s multiple comparisons test standardized to B27(INS^−^) CHIR^−^ condition. (D and E) Data are presented as medians and compared using the Kruskal-Wallis test. *n* = 3 independent experiments from 3 different cell lines (SCVI-273, SCVI-114, and SCVI-111), unless specified different. ^∗^*p* < 0.05, ^∗∗^*p* < 0.01, ^∗∗∗^*p* < 0.001, and ^∗∗∗∗^*p* < 0.0001; ns, not significant.

Previous studies in hiPSCs have shown that the cell-cycle pathways act as a deterministic restriction on the pluripotent state of stem cells. Pluripotency control is connected to the cell-cycle machinery, where S and G2 phase-specific pathways deterministically restrict pluripotent state dissolution (Gonzales et al., 2015). In this process, pathways such as PI3K/AKT, transforming growth factor β, MEK, and FGF in G1 phase potentially permit the initiation of differentiation (Gonzales et al., 2015; Liu et al., 2019). Based on our previously described hiPSC-CM expansion media (Buikema et al., 2020; Maas et al., 2021), we identified two main pathway activators for cell division in Figure 1. To further investigate the impact of CHIR and insulin on the maturity of hiPSC-CMs, we established four distinct treatment groups based on the presence or absence of CHIR (3 μM) and/or insulin, denoted as “B27(INS^−^) CHIR^−^,” “B27(INS^+^) CHIR^−^,” “B27(INS^−^) CHIR^+^,” and “B27(INS^+^) CHIR^+^.” Using quantitative reverse-transcription PCR (RT-qPCR) and immunofluorescence assays, we examined mRNA expression levels associated with cardiomyocyte proliferation or maturation (Figures 2A and S2A–S2F). We found a 5-fold higher expression of the embryonic isoform of myosin heavy chain (*MYH6*) in hiPSC-CMs treated with CHIR when compared to only insulin or no stimuli present in the media (Figure 2B). In hiPSC-CMs treated with CHIR, we confirmed activated Wnt target gene expression by *AXIN2* and observed increased expression of the cell-cycle gene *CCND2* (Figure 2B). Interestingly, we found that withdrawal of CHIR and/or insulin gradually led to increased expression of the adult isoform of myosin heavy chain (*MYH7*), the mature/immature myosin heavy chain ratio (*MYH7/6*), and the Troponin I (*TNNI3*) gene. Stable transcriptional activity was found for β-catenin (*CTNNB1*) or the PPAR transcription factors involved in energy homeostasis (Figure 2B). To evaluate the expression of sarcomere proteins after exposure to the defined four treatment conditions, we stained for one cardiac marker, cardiac troponin T (cTnT), and one cardiomyocyte maturation marker, ventricular specific myosin light chain 2v (MLC2v). Notably, we found that MLC2v signal intensity was most highly expressed in the non-proliferative hiPSC-CMs, which were cultured without insulin or CHIR (Figures 2C and 2D). Additionally, we noted a significant increase in cell size upon withdrawal of CHIR from the media (Figures 2C and 2E). Taken together, these findings indicate that insulin and CHIR inhibit the expression of mature cardiomyocyte markers in hiPSC-CMs.

### Sarcomere structure and contractile properties in response to insulin and GSK3 inhibition

**Figure 3:**
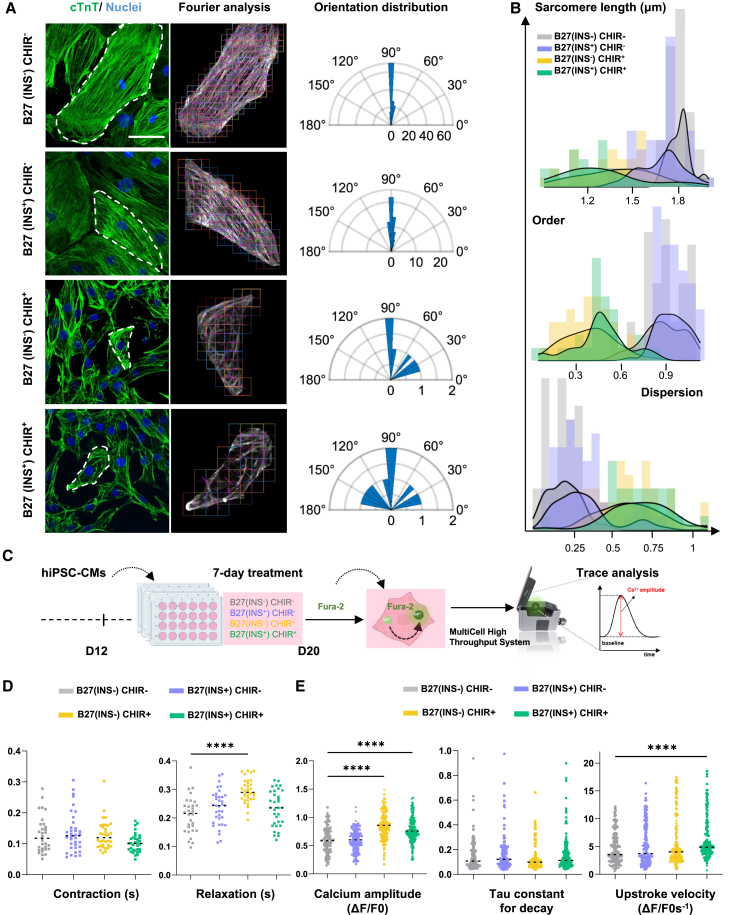
**Structural and functional characterization of contractile hiPSC-CMs treated with insulin and/or CHIR99021** (A) Representative immunofluorescence images of cardiac Troponin T (cTnT) (green) staining and automated sarcomere orientation and distribution analysis using the MorphoScript tool. Nuclei visualized with DAPI (blue). Scale bar, 50 μm. (B) Sarcomere length (upper), order (middle), and dispersion (lower) for indicated treatments presented as histograms. The y axis on each histogram indicates normalized density. (C) Schematic of treatments followed by calcium transient and contractility analysis. (D) Scatterplots of contractility analyses; contraction time (time to peak 70%) and relaxation time (time to baseline 70%) in ▪ = SCVI-273, 3 biological experiments in 10–12 technical replicates per condition. (E) Scatterplots of calcium handling dynamics; calcium amplitude, upstroke velocity, and Tau decay. (D and E) Data are represented as mean ± SD. *n* ≥ 30 cells for each condition were analyzed from *N* = 3 independent experiments from 3 different cell lines (▪ = SCVI-273, ♦ = SCVI-202, and ● = SCVI-114) (Figure S4), unless specified otherwise. Comparisons are performed by Kruskal-Wallis test followed by Dunn’s multiple comparisons test with B27(INS^−^) CHIR^−^ as reference condition. Not significant, *p* > 0.05, ^∗^*p* < 0.05, ^∗∗^*p* < 0.01, ^∗∗∗∗^*p* < 0.0001.

The regulation of cardiac contractile function is dependent upon the structure and organization of the sarcomeres. In addition to the observed impact of insulin and CHIR on the expression of sarcomere-associated genes and protein profiles, we further conducted a previously established morphological analysis of sarcomere structure under the designed conditions (Homan et al., 2021). Our analysis of the order and dispersion parameters demonstrated that hiPSC-CMs cultured under the B27(INS^−^) CHIR^−^ condition displayed the most highly organized cTnT striations when compared to cells cultured in B27(INS^+^) CHIR^−^ (Figures 3A, 3B, and S3A). In contrast to hiPSC-CMs cultured in B27(INS^−^) CHIR^−^, the addition of CHIR (B27(INS^−^) CHIR^+^) led to a significant increase in dispersion (0.261 versus 0.653) (Figure 3B, Dispersion) and a decrease in the order parameter (0.785 versus 0.361) (Figure 3B, Order). This difference in dispersion in response to CHIR was amplified by the addition of insulin (B27(INS^+^) CHIR^+^) (0.346 versus 0.706) (Figure 3B, Dispersion). Moreover, average sarcomere length was 1.703 μm in the B27(INS^+^) CHIR^−^ condition, versus an increased sarcomere length with an average of 1.798 μm in hiPSC-CMs cultured in B27(INS^−^) CHIR^−^ (Figure 3B, Sarcomere length). In contrast, the addition of CHIR significantly reduced the average sarcomere length to 1.467 and 1.401 μm in hiPSC-CMs cultured in B27(INS^−^) CHIR^+^ and B27(INS^+^) CHIR^+^, respectively (Figure 3B, Sarcomere length). Previous studies have shown an association between sarcomere function and polyploidization (Pettinato et al., 2021; Yuan et al., 2022), and, in line with that, we observed a larger proportion of mononucleated hiPSC-CMs in the presence of CHIR, versus a larger proportion of multinucleated cells in the absence of CHIR, independent from insulin (Figures S3B–S3D).

Next, we evaluated the contraction, relaxation, and calcium kinetics of hiPSC-CMs (Dinani et al., 2023) with and without CHIR and/or insulin (Figure 3C). In all four conditions we found contractile cells with active calcium handling, indicating that these cells were functional cardiomyocytes. Contraction duration was not altered significantly, while relaxation was shortened in the absence of pathway stimuli (Figure 3D). Next, calcium tracings showed similar Tau decay for all four conditions, while calcium amplitude and upstroke velocity were shorter in the absence of CHIR; however, spontaneous beating frequencies also differed per condition (Figures 3E and S4). Taken together, these results highlight that mostly CHIR and to a lesser extent insulin signaling negatively impact sarcomere development. At the two-dimensional level all treatment groups were functional, but no important signs of mature contractility or calcium kinetics were observed in the absence of growth stimuli.

### Metabolic function and substrate utilization in response to GSK3β inhibition and insulin

**Figure 4:**
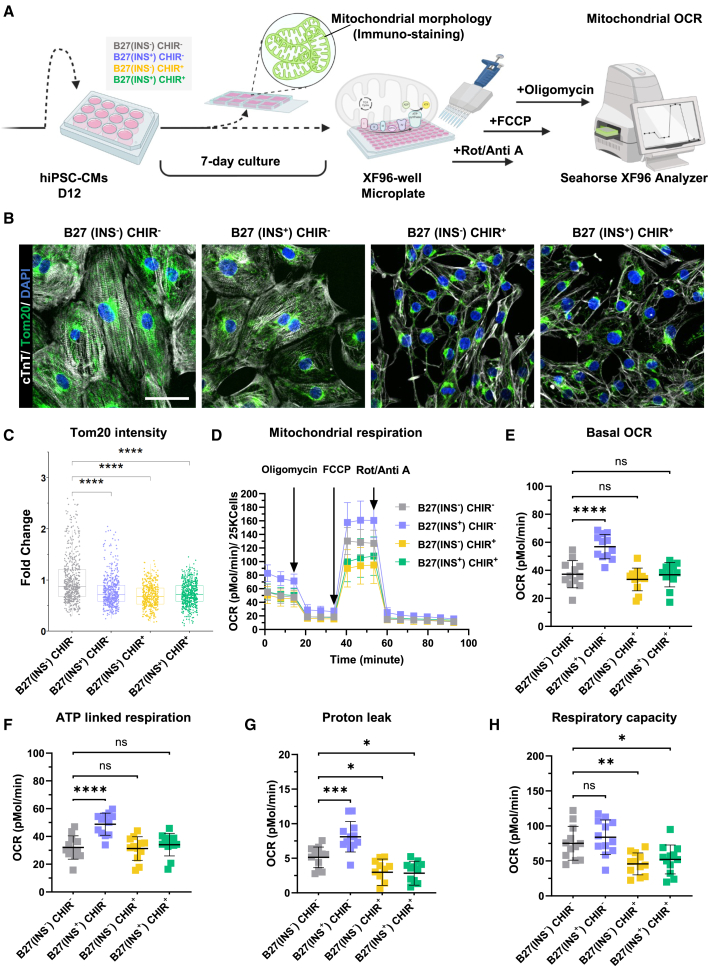
**The effects of insulin andCHIR99021on mitochondrial distribution and function** (A) Schematic of experimental setup for mitochondria assays. (B) Representative immunofluorescence images of cTnT (white) and Tom20 (green). DAPI staining for nuclei (blue). Scale bar, 200 μm. (C) Quantification of Tom20 immunofluorescence intensity in ±550 cells/condition, relative to B27(INS^−^) CHIR^−^. (D) Overview of changes in mitochondrial oxygen consumption rate in response to oligomycin, FCCP, (carbonyl cyanide 4-(trifluoromethoxy)phenylhydrazone), and Rot/Anti A (rotenone and antimycin A). Per well 25K cells were plated. (E) Basal oxygen consumption rate (OCR) calculated by subtracting the non-mitochondrial respiration from the basal OCR. (F) ATP-linked respiration calculated based on the OCR after oligomycin injection. (G) Proton leak obtained as the remaining OCR, which is not coupled to ATP-linked respiration. (H) Respiratory capacity of mitochondria measured by deduction of basal OCR from the maximum OCR in response to FCCP injection. (C) Data are presented as medians and compared using the Kruskal-Wallis test, *N* ≥ 5 independent cultures from 3 batches of SCVI-273 cell line. (D–H) Data are presented as mean ± SD, and statistical significance tested with one-way ANOVA followed by Tukey’s multiple comparison test. *n* ≥ 11 independent cultures from 3 batches of SCVI-273 cell line. ^∗^*p* < 0.05, ^∗∗^*p* < 0.01, ^∗∗∗^*p* < 0.001, ^∗∗∗∗^*p* < 0.0001; ns, not significant.

The metabolic compartmentalization of the embryonic heart displays an enhanced glycolytic program in the compacted myocardium, versus more fatty acid-oxidative metabolic utilization in the more differentiated trabeculae (Menendez-Montes et al., 2016). This metabolic compartmentalization follows the pattern of highly proliferative cardiomyocytes in the compacted myocardium versus more differentiated trabeculae areas (Buikema et al., 2013, 2014; Jeter and Cameron, 1971; Luxán et al., 2013; Ye et al., 2015). To characterize mitochondrial content and function, we used day 12 immature hiPSC-CMs and treated them under the described conditions (Figure 4A). After the four different treatment regimens, we found the highest mitochondria content (Tom20 intensity/cell) in hiPSC-CMs cultured without insulin and CHIR, versus insulin alone and/or CHIR/insulin combinations (Figures 4B and 4C). Moreover, we mostly observed a perinuclear localization of mitochondria in cells cultured with CHIR, as an indicator of immaturity, versus combined perinuclear and cytoplasmic localization of mitochondria in cells cultured without CHIR (Figure 4B). In contrast, insulin played a facilitating role in hiPSC-CMs in terms of mitochondrial respiration, as evidenced by the enhancements observed in basal oxygen respiration, ATP-linked respiration, proton leak, and spare reserve capacity (Figures 4D–4H). Conversely, the inhibition of GSK3β led to further reductions in these parameters of mitochondrial respiration, irrespective of insulin presence (Figures 4D–4H). Additionally, substrate-blocking assays performed in different media compositions revealed that in hiPSC-CMs cultured without insulin, and supplemented with fatty acids, the oxygen consumption rate was restored to a level comparable to when insulin was present in the media (Figures S5A–S5D). This indicated a relative dependency on fatty acids for ATP production in cells cultured without insulin and CHIR. To this end we verified the uptake of fatty acids in cells treated with all four groups and indeed found that in the absence of insulin and CHIR, the hiPSC-CMs had the highest uptake of fatty acids, as an indicator of more maturity of these cells (Figures S6A–S6C). Interestingly, insulin was less essential than CHIR in this process, and the addition of fatty acids to the culture media induced a switch to mitochondrial oxidative phosphorylation and decreased glycolysis dependency and glutamine metabolism (Figures S5B–S5D). These results collectively indicate that insulin is not essential for the metabolic switch of hiPSC-CMs but is required for the oxidation of substrates such as glucose and glutamine. Altogether, those results indicate that insulin and CHIR stimulation led to decreased mitochondria content, lower energy production, and repression of fatty acids utilization in hiPSC-CMs.

### RNA sequencing identifies FOXO as candidate mediating the interplay between proliferation and maturation of hiPSC-CMs

**Figure 5:**
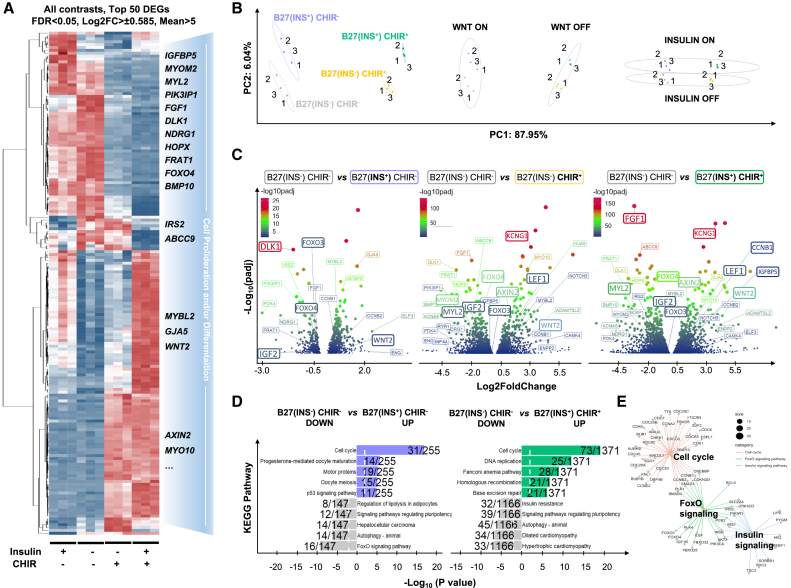
**RNA sequencing analysis of hiPSC-CMs treated with insulin and/or CHIR99021** (A) Heatmap of top 50 differentially expressed genes (DEG) filtered based on base mean ≥ 5, log2fc >= 0.585 or =< −0.585, FDR < 0.05; pink to red highlights increased gene expression, and the light to dark blue denotes decreased gene expression. (B) PCA plots distinguishing between CHIR and/or insulin treatment. *N* = 3 independent cultures from SCVI-273 cell line. (C) Volcano plots of highlight DEGs of the following conditions: B27(INS^+^) CHIR^−^, B27(INS^−^) CHIR^+^, and B27(INS^+^) CHIR^+^ each compared versus B27(INS^−^) CHIR^−^ (Table S3). (D) KEGG enrichment analysis between conditions. (E) Molecular network of insulin signaling pathway and FOXO signaling pathway for Wnt signaling-activated cell cycle.

To enhance our understanding of the effects of insulin and CHIR on proliferation and maturation in immature hiPSC-CMs, we conducted high-throughput transcriptome sequencing (RNA sequencing [RNA-seq]) to evaluate the differences among the described culture conditions. The top 50 differentially expressed genes (DEGs) were highlighted to those linked to embryonic pathways in cardiac differentiation and development. Notably, these include sarcomere structure-related genes *MYL2* and *MYOM2*, as well as up- and downstream regulating genes of the insulin/PI3K-Akt signaling pathway, such as *IGFBP5*, *PIK3IP1*, and *FOXO4*; WNT signaling genes *WNT2* and *AXIN2*; and other genes involved in proliferation and differentiation such as *BMP10*, *MYBL2*, *FRAT1*, *HOPX*, and *DLK1* (Figure 5A). Principal-component analysis (PCA) revealed that CHIR contributed to the greatest variation (87.95%) among these DEGs, involving 5,336 variables. In contrast, insulin only contributed 6.04% (994 variables), and the combined influences of insulin and CHIR accounted for 7,003 variables (Figure 5B). Notably, our volcano plots displayed a gradient upregulation of the *WNT2* gene activated by CHIR, interestingly, with a sequential increase in significance following the addition of insulin, CHIR, and the combination of insulin and CHIR (Figure 5C and Table S3). This pattern indicates that insulin and CHIR exhibit effects on *WNT2* activation. Similarly, sarcomere-associated genes *MYOM2* and *MYL2* showed a gradual downregulation induced by insulin and/or CHIR (Figure 5C), reinforcing our observation of their effects on proliferation and sarcomere development for immature hiPSC-CMs (Figures 1 and 3). Moreover, this impact further extended to cellular gap junctions (*GJA5*) and also affected differentiation-associated genes (*MYOM2*, *DLK1*, *KCNA5*) (Figure 5C). Interestingly, the FOXO family member *FOXO4* also displayed a gradual upregulation in the absence of insulin and CHIR, such as that observed with *WNT2* activation (Figure 5C). To explore functional batch annotation and ontology enrichment of the DEGs, we conducted Kyoto Encyclopedia of Genes and Genomes (KEGG) pathway analysis. As expected, the activated WNT target genes in DEGs showed significant enrichment in cell-cycle-related pathways (Figures 5D and S2K). Moreover, our analysis suggested that this effect is likely due to the suppression of FOXO via insulin (Figures 5D and 5E). In addition to DEGs and pathway enrichment, gene ontology (GO) analysis highlighted significant changes in molecular functions related to tubulin and actin binding (Figures S2G–S2I). Correspondingly, the effects of insulin and/or CHIR were specifically evident in the contractile machinery, involving Z-disc, sarcomere, myofibril, actinin binding, and I band, in addition to cell-cycle activity (Figure S2J). Taken together, these data support the observation that activation of cell-cycle genes is inversely associated with mature cardiomyocyte genes. Moreover, our analysis identifies FOXO as a potential downstream target of insulin signaling in hiPSC-CMs renewal.

### Molecular inhibition of FOXO signaling rescues cell-cycle exit of immature hiPSC-CMs

**Figure 6:**
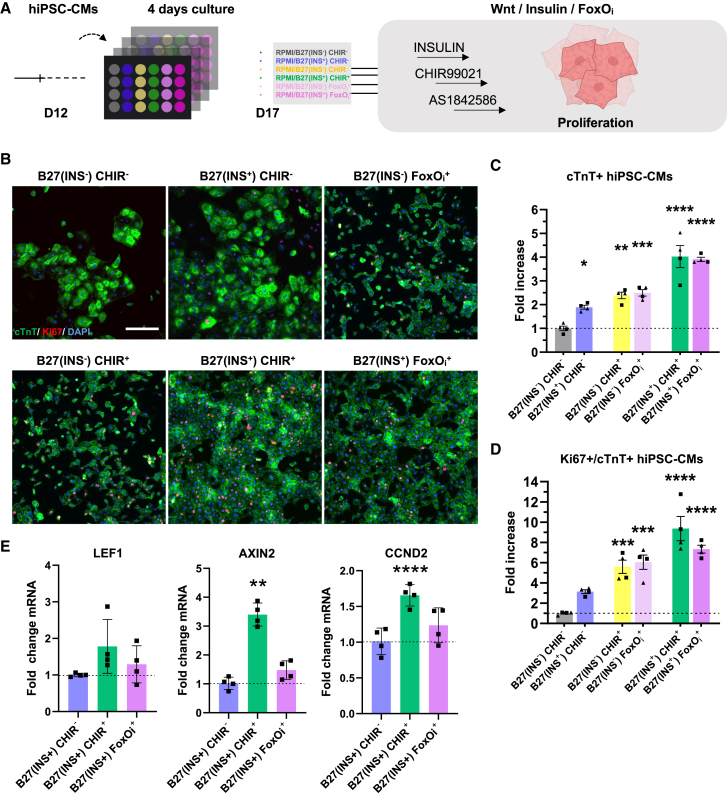
**WNT activation (CHIR99021)/FOXOinhibition (AS1842856) promotes early immature hiPSC-CM proliferation** (A) Schematic of timeline for investigating the effects of Wnt/Insulin/FOXO inhibition (FOXOi) on the proliferation of hiPSC-CMs. (B) Representative immunofluorescence images of hiPSC-CMs subjected to cTnT antibody (green), Ki67 antibody (red), and DAPI for indicated conditions. Scale bar, 200 μm. (C and D) Quantification of cTnT-positive hiPSC-CMs (C) and the fraction of Ki67-and cTnT-positive hiPSC-CMs (D) by fold increase under the indicated culture conditions. Data are presented as mean ± SEM. *n* = 4 independent cultures from 2 different cell lines (▪ = SCVI-273, ▲ = SCVI-111). (E) Fold mRNA changes of distinct Wnt target genes after 24 h treatment with the indicated culture conditions. Data are presented as mean ± SD. *N* = 4 biological replicates of each 3 technical replicates were performed using the SCVI-273 line. Data normalized to GAPDH. Statistical significance tested using one-way ANOVA followed by Dunn’s test for multiple comparisons relative to control B27(INS^−^) CHIR^−^ (C and D) and B27(INS+) CHIR- (E), ^∗^*p* < 0.05, ^∗∗^*p* < 0.01, ^∗∗∗^*p* < 0.001, and ^∗∗∗∗^*p* < 0.0001.

Recently, the molecular inhibition of FOXO with AS1842586 was found to increase hiPSC-CMs proliferation (Schade et al., 2022). We further conducted the investigation into the role of insulin/FOXO signaling in CHIR-stimulated proliferation of hiPSC-CMs (Figure 6A). When hiPSC-CMs were cultured under FOXO inhibition with AS1842586, we observed a 6-fold increase in the proliferation rate of immature hiPSC-CMs (D12) after 4 days. This led to an approximate 2.4-fold increase in the number of cTnT-positive cells among hiPSC-CMs, even in the absence of insulin in the culture. Remarkably, this outcome was comparable to the effect observed with CHIR alone (without insulin), which stimulated an increase in both cTnT-positive cell number and the proliferation rate (Ki67^+^/cTnT^+^) of immature hiPSC-CMs (D12) (Figures 6B–6D). This suggests that the activation of FOXO in the culture of immature hiPSC-CMs inhibited proliferation, as predicted by RNA-seq analysis (Figures 5C and 5D). However, this proliferative effect of FOXO inhibition did not seem to be induced through direct upregulation of Wnt target genes (Figure 6E). Significantly, when insulin was reintroduced into the culture in the presence of AS1842586, there was a 6.5-fold increase in the proliferation rate and a 4-fold increase in cTnT-positive cell number of immature hiPSC-CMs compared to the culture without insulin or AS1842586 stimulation (Figures 6C and 6D). Notably, the increase in cTnT-positive cell number induced by insulin and FOXO inhibition was comparable to the CHIR-stimulated proliferation of hiPSC-CMs (Figure 6C). Importantly, to rule out the possibility that the increase in cTnT-positive cell numbers and the proliferation rate (Ki67+/cTnT+) we observed is rather due to a protective effect from apoptosis, we show that all of the conditions maintain equal cellular viability (∼90%) (Figure S7). Collectively, the inhibition of FOXO facilitated proliferation of hiPSC-CMs, presumably in part by shortcutting the insulin-Akt route.

### Stage-specific effects of insulin and Wnt signaling pathways on early- and late-stage hiPSC-CMs

**Figure 7:**
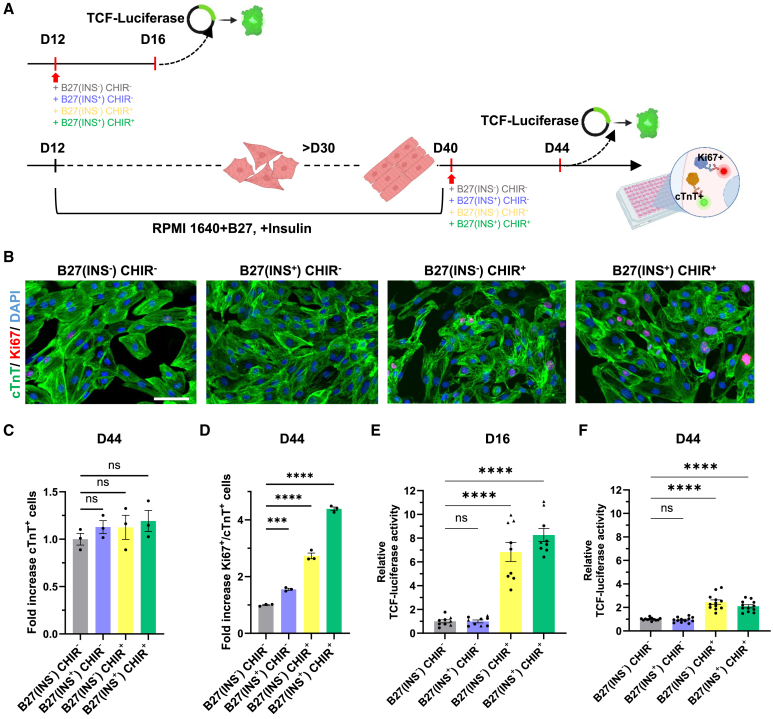
**Impaired effects of insulin and Wnt/β-catenin signaling on late-stage hiPSC-CM proliferation** (A) Schematic of early-stage and late-stage hiPSC-CMs proliferation assays. (B) Representative immunofluorescence images of hiPSC-CMs subjected to cTnT antibody (green), Ki67 antibody (red), and DAPI under the designed culture conditions. Scale bar, 50 μm. (C and D) Quantification of cTnT+ hiPSC-CMs (C) and fraction of Ki67+/cTnT+ hiPSC-CMs (D) in late-stage (D44) iPSC-CMs by fold increase under the indicated culture conditions. (E and F) Fold increase in luciferase activity after transfection of luciferase reporter (TOPFlash) in early-stage (D16) hiPSC-CMs (E) and late-stage (D44) hiPSC-CMs (F). Data presented as mean ± SEM. *n* ≥ 3 independent cultures from 2 different hiPSC lines (● = SCVI-114, ▲ = SCVI-111). Statistical significance was assessed by one-way ANOVA followed by Dunn’s test for multiple comparisons relative to B27(INS^−^) CHIR^−^. Not significant (ns), *p* > 0.05, ^∗∗∗^*p* < 0.001, and ^∗∗∗∗^*p* < 0.0001.

In the developing heart, the proliferative capacity of fetal cardiomyocytes rapidly diminishes shortly after birth (Li et al., 1996). This phenomenon has also been observed in hiPSC-CMs following maturation. To this end, we prolonged the culture of hiPSC-CMs in the widely used RPMI 1640 basal medium supplemented with B27, including insulin, to 40 days following the initial differentiation at day 12 (Figure 7A). To evaluate potential stage-specific effects of embryonic proliferation signals, we introduced insulin and/or CHIR in the “late-stage” hiPSC-CMs at day 40. We observed up to a 4-fold increase in Ki67+/cTnT+ cells upon stimulation with CHIR and insulin when compared to no insulin/CHIR. The stimulation of “late-stage” hiPSC-CMs with insulin and CHIR did not result in more cTnT+ cell numbers, indicating that only a small subset of “late-stage” hiPSC-CMs was still responsive to the signaling molecules (Figures 7B–7D). In this context, we examined the activity of β-catenin/TCF, a center effector of the canonical Wnt/β-catenin signaling pathway that promotes cell-cycle progression (Nusse and Clevers, 2017), via TOPFlash expression in hiPSC-CMs. Our data revealed a significant decreased TCF activity upon CHIR stimulation in “late-stage” hiPSC-CMs when compared to “early-stage” hiPSC-CMs (Figures 7E and 7F). Collectively, our findings demonstrate that insulin and CHIR exhibit stage-specific effect on proliferation, associated with lower nuclear TCF activation in “late-stage” versus “early-stage” hiPSC-CMs.

## Discussion

Here we report dual effects of insulin and CHIR99021 on proliferation of immature hiPSC-CMs and blocking of maturation, while the removal of these pathway activators results in rapid cell-cycle exit (Figure 1) and further differentiation (Figures 2B and 3). While CHIR inhibits GSK3β and activates canonical Wnt signaling via TCF-mediated target gene transcription, we also identify a novel interplay between insulin-Akt and FOXO transcription as regulator between proliferation and maturation of immature hiPSC-CMs (Figures 5 and 6).

In the context of B27, commonly used as a supplement to the basal RPMI 1640 medium for hiPSC-CM culture, insulin, a component in B27, acts as a stimulant of the PI3K-AKT pathway, leading to the phosphorylation of GSK3β (Abel, 2021; Buikema et al., 2020). Our work provides insights into the positive effects of insulin on proliferation and energy production in hiPSC-CMs (Figures 1 and 4). On the contrary, insulin has a negative impact on the differentiation and organization of the sarcomeres (Figures 2 and 3). Therefore, the concentration of insulin in the media would be an important consideration for the production of more mature cardiomyocytes.

Our RNA-seq analysis revealed that insulin upregulated cell-cycle signaling pathways, while the removal of insulin led to a significant upregulation of FOXO signaling (Figure 5). Undoubtedly, the insulin-mediated PI3K-Akt signaling cascade serves as a pivotal regulator of FOXO (Litwiniuk et al., 2016). This signaling pathway plays a crucial role in regulating proliferation in both cultured primary embryonic cardiomyocytes (Evans-Anderson et al., 2008) and hiPSC-CMs (Schade et al., 2022). In our studies we found that addition of the FOXO inhibitor AS1842856 to media depleted from insulin and CHIR resulted in proliferation of hiPSC-CMs. This suggests that without insulin in the media, in part FOXO acts as a negative regulator of hiPSC-CM expansion, and subsequent inhibition of FOXO leads to cell division. Moreover, our experiments combining insulin with a FOXO inhibitor demonstrated a more powerful proliferative response, also indicating indirect effects of AS1842856 (Figure 6). These findings suggest a promising direction for further investigation.

Mills et al.’s investigation into human pluripotent stem cell-derived cardiac organoids revealed that the absence of insulin in the culture medium, when exposed to palmitate, induces cell-cycle arrest and instigates a metabolic shift (Mills et al., 2017). Furthermore, Garay et al.’s work underscored the significance of concurrent inhibition of both the PI3K-AKT pathway and mitogen-activated protein kinase pathway that promotes the maturation of hiPSC-CMs across multiple facets, encompassing transcriptional profiles, sarcomere development, metabolism, and electrophysiology (Garay et al., 2022). In our studies we found that CHIR has significant negative influences on the localization and function of mitochondria. Moreover, insulin had a positive effect on overall metabolism of hiPSC-CMs. Interestingly, in the absence of CHIR and insulin, we found an increase in mitochondria and a preference for fatty acid uptake (Figures 4, S5, and S6).

Notably, our assays in “early-stage” and “late-stage” cardiomyocytes indicated a stage-specific effect of CHIR and insulin on the robust proliferation of hiPSC-CMs. In part, this could be the result of impaired TCF activation by β-catenin such as investigated in the TOPFlash luciferase reporter assay (Figure 7). Alternatively, β-catenin may already have different transcriptional effects in these “late-stage” hiPSC-CMs when compared to the “early-stage,” such as has been studied by Quaife-Ryan et al. before (Quaife-Ryan et al., 2020). Moreover, the treatment of hiPSC-CMs with CHIR results in rapid disorganization of sarcomeres either via canonical Wnt signaling or via direct effects on GSK3, resulting in proliferation. Additional evidence suggests that sarcomeres negatively regulated cell replication and promoted polyploidization by activating p53-dependent DNA damage (Pettinato et al., 2021). In line with that evidence, our study observed a most profound effect of insulin and CHIR99021 on producing mononuclear hiPSC-CMs (Figure S3).

Moreover, we found that CHIR99021-mediated GSK3β inhibition disrupts sarcomere structure, which is independent of insulin (Figures 3A and 3B). This implies that inducing sarcomere disassembly through CHIR could be a potential strategy to promote renewal of cardiomyocytes. Although no dramatic effects in calcium handling and contractility were observed in the presence or absence of CHIR and insulin, this could be explained by a number of factors such as increased cell growth, varying spontaneous beating frequencies (Figure S4E), and the two-dimensional culture set-up. For future studies it would be of interest to test the effect of Wnt and insulin in three-dimensional cardiac tissues.

In summary, we provide novel insights into the cues to maintain cardiomyocyte proliferation versus maturation via an interplay between the insulin/FOXO and Wnt/GSK3β/β-catenin/TCF signaling routes. The understanding of embryonic signaling routes would be of importance for future regenerative strategies in heart failure.

## Experimental procedures

### HiPSCs culture and differentiation to hiPSC-CMs

Four independent hiPSC lines from the Stanford Cardiovasculair Institute (SCVI) biobank (SCVI-273, SCVI-114, SCVI-202, and SCVI-111) were cultured on Matrigel-coated general cell culture plates and maintained in E8 basal medium supplemented with E8 supplement (1×) (Life Technologies). Medium changes were performed daily, and passaging and initiation of differentiation were performed at 70%–80% confluency. For differentiation into hiPSC-CMs, we used the Wnt-modulation protocol with chemical GSK3 (CHIR99021) and Wnt (C59) inhibitors as previously described (Lian et al., 2013). For the TC1133-ACTN2-Citrine-FUCCI hiPSC line (for a more detailed explanation and characterization of the cell line, see Kim et al., 2024), a different differentiation protocol was used; at day 0 we subjected the cells to base medium (RPMI 1640 + GlutaMAX, 2% B27(INS-), 200 μmol/L L-ascorbic acid, 1 mmol/L Na-pyruvate, 100 U/mL penicillin, 100 μg/mL streptomycin) supplemented with 9 ng/mL Activin A (Bio-Techne), 1 μmol/L CHIR99021 (Merck Chemicals GmbH), 5 ng/mL BMP4 (Bio-Techne), and 5 ng/mL FGF (PreproTech) for 3 days. Afterward, the base medium was supplemented with 5 μmol/L IWP4 (ReproCELL) for cardiomyocyte differentiation for 9 days. All hiPSC lines were between passage number 20 and 35.

### Immunofluorescence staining and confocal microscopy imaging

See supplemental information for a detailed description and key resource table for details of antibodies used.

### RT-qPCR

See supplemental information and Table S1 for a list of primers used.

### Contractility measurements and calcium transient analysis

See supplemental information.

### Seahorse XF Mito stress assay

See supplemental information.

### Seahorse XF Mito Fuel Flex assay

See supplemental information.

### Fatty acid uptake assay

See supplemental information.

### Bulk RNA-seq analysis

See supplemental information and GEO submission GSE278598.

### Luciferase-based LEF/TCF activity analysis

See supplemental information.

### Quantification and statistical analysis

Statistical analysis and visualization were conducted using GraphPad Prism version 9 software and/or R v4.0.2. Data were tested for normality using Pearson D’Agostino’s K-squared test and analysis of QQ plots. For normally distributed data, student’s t test or ordinary one-way ANOVA followed by Tukey’s *post hoc* multiple comparisons (comparisons between all group means) or Dunn’s multiple comparisons test (comparisons to control condition) was performed. Non-parametric data with multiple groups were analyzed using Kruskal-Wallis test followed by Dunn’s multiple comparisons test. The levels of significance were represented by the following *p* values: *p* > 0.05 (ns, not significant), ^∗^*p* ≤ 0.05, ^∗∗^*p* ≤ 0.01, ^∗∗∗^*p* ≤ 0.001, and ^∗∗∗∗^*p* ≤ 0.0001.

### hiPSC lines

Research on hiPSC was approved by the medical ethical committee at Amsterdam UMC, the Netherlands. A detailed list of hiPSC lines used for the experiments is provided in the STAR Methods in supplemental information.

## Resource availability

### Lead contact

Request for further information or more detailed protocols should be directed to and will be fulfilled by the corresponding author, Jan W. Buikema (j.w.buikema@amsterdamumc.nl).

### Materials availability

This study did not generate new unique reagents.

### Data and code availability

The RNA-seq data were reposited under GEO accession number GSE278598.

## Acknowledgments

We thank Joseph C. Wu from Stanford University for providing lines SCVI-111, SCVI-114, SCVI-202, and SCVI-273. We thank Amsterdam UMC (location VUmc; O2 building) Microscopy and Cytometry Core Facility (MCCF) for the technological support. We thank Babu Kurakula from Amsterdam University Medical Center for sharing Renilla luciferase reporter plasmid. This research was supported by grants from the Chinese Scholarship Council (CSC no. 201706170068) (Q.Y.), the Dutch Heart Foundation Dekker Senior Clinical Scientist grant (J.W.B.) and the Netherlands Organization for Scientific Research (NWO VICI, grant 91818602)(JvV).

## Author contributions

Experimental design, data analysis, and manuscript writing, Q.Y., D.V., and J.W.B.; experimental procedures and Figures 1 and S1, Q.Y., R.K., E.S., and D.V.; Figures 2 and S1, Q.Y.; Figures 3 and S2A–S2F, S.A.S., C.D.M., and Q.Y.; Figures 4 and S3, Q.Y. and R.D.; Figure S4, R.D., D.V., and Q.Y.; Figures 5 and S6, Q.Y. and D.V.; Figure 6, Q.Y., L.J.M.K., and D.V.; Figure 7, Q.Y. Manuscript reading and editing was done by J.v.d.V., D.W.D.K., L.C.Z., D.v.d.V., J.H., and R.A.B. All authors have read and agreed to the published version of the manuscript.

## Declaration of interests

The authors declare no competing interests.

## Supplemental information

Document S1. Figures S1–S7, Tables S1–S3, and STAR Methods

Document S2. Article plus supplemental information
