# Supplementary material for: Glycogen synthase kinase-3 inhibition and insulin enhance proliferation and inhibit maturation of human iPSC-derived cardiomyocytes via TCF and FOXO signaling
Source: Stem Cell Reports. 2024 Dec 5;20(1):102371. doi: 10.1016/j.stemcr.2024.11.001 (PMC11784517; doi:10.1016/j.stemcr.2024.11.001)
Supplement: Document S1. Figures S1–S7, Tables S1–S3, and STAR Methods [file mmc1.pdf]

Stem Cell Reports, Volume *20*

Supplemental Information

Glycogen synthase kinase-3 inhibition and insulin enhance proliferation and inhibit maturation of human iPSC-derived cardiomyocytes via TCF and FOXO signaling

Qianliang Yuan, Devin Verbueken, Rafeeh Dinani, Rosa Kim, Eric Schoger, Chloé D. Morsink, Shamim Amiri Simkooei, Luuk J.M. Kemna, Jesper Hjortnaes, Diederik W.D. Kuster, Reinier A. Boon, Laura Cecilia Zelarayan, Jolanda van der Velden, and Jan W. Buikema

Supplemental Information

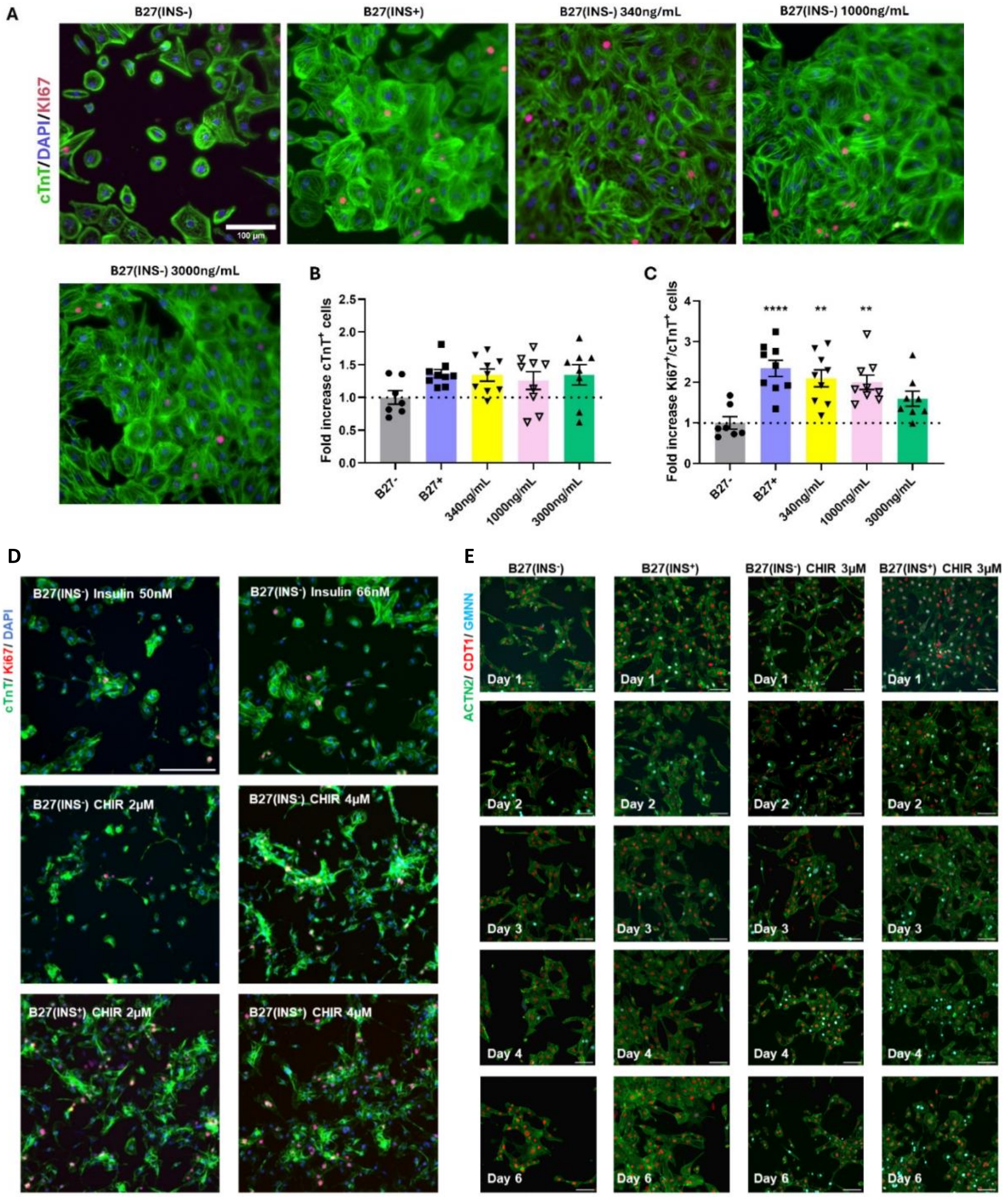

Figure S1: Insulin concentration screening for proliferative response in immature hiPSC-CMs and monitoring of cell cycle activity. (**A**) Representative immunofluorescence images of immature SCVI-273 hiPSC-CMs that were cultured over 7 days exposed to an exponential array of Insulin concentration. Cells were stained for cTnT (green), Ki67 (red), and nuclei (DAPI, blue). (**B**) Quantification of $cTnT^{+}$ hiPSC-CMs cultured in B27(INS+), B27(INS-) + 340ng/mL (~58nM), B27(INS-) + 1000ng/mL (~170nM), B27(INS-) + 3000ng/mL (~500nM), with fold increases relative to B27(INS-) condition. (**C**) Quantification of $Ki67^{+}$ cells among $cTnT^{+}$ hiPSC-CMs for all conditions of (B) with fold increased relative to B27(INS-). Data are presented as mean +/- SEM. Experiment was performed in

triplicate, every data point represents analysis of one image. After confirmation of normality using the D'Agostino & Pearson test, statistical significance was tested using one-way ANOVA followed by Dunn's test for multiple comparisons relative to B27(INS-). $^{**}p < 0.01$, $^{****}p < 0.0001$. (**D)** Representative immunofluorescence images of hiPSC-CMs exposed to the gradient concentrations of stimuli, Insulin and/or CHIR in 7 days. The cells were stained for cTnT (green), Ki67 (red), and nuclei (DAPI, blue). Scale bar=200 µm. (**E**) Live cell imaging of TC1133-ACTN2-Citrine-FUCCI hiPSC-CMs (ACTN2+, green) in the absence or presence of CHIR and Insulin. G1-cardiomyocytes are labeled with mCherry (CDT1, red) and S/G2-cardiomyocytes are labeled with ECFP (GMNN, light blue). Cells were monitored over a time course of 6 days. Scale bar=100 µm.

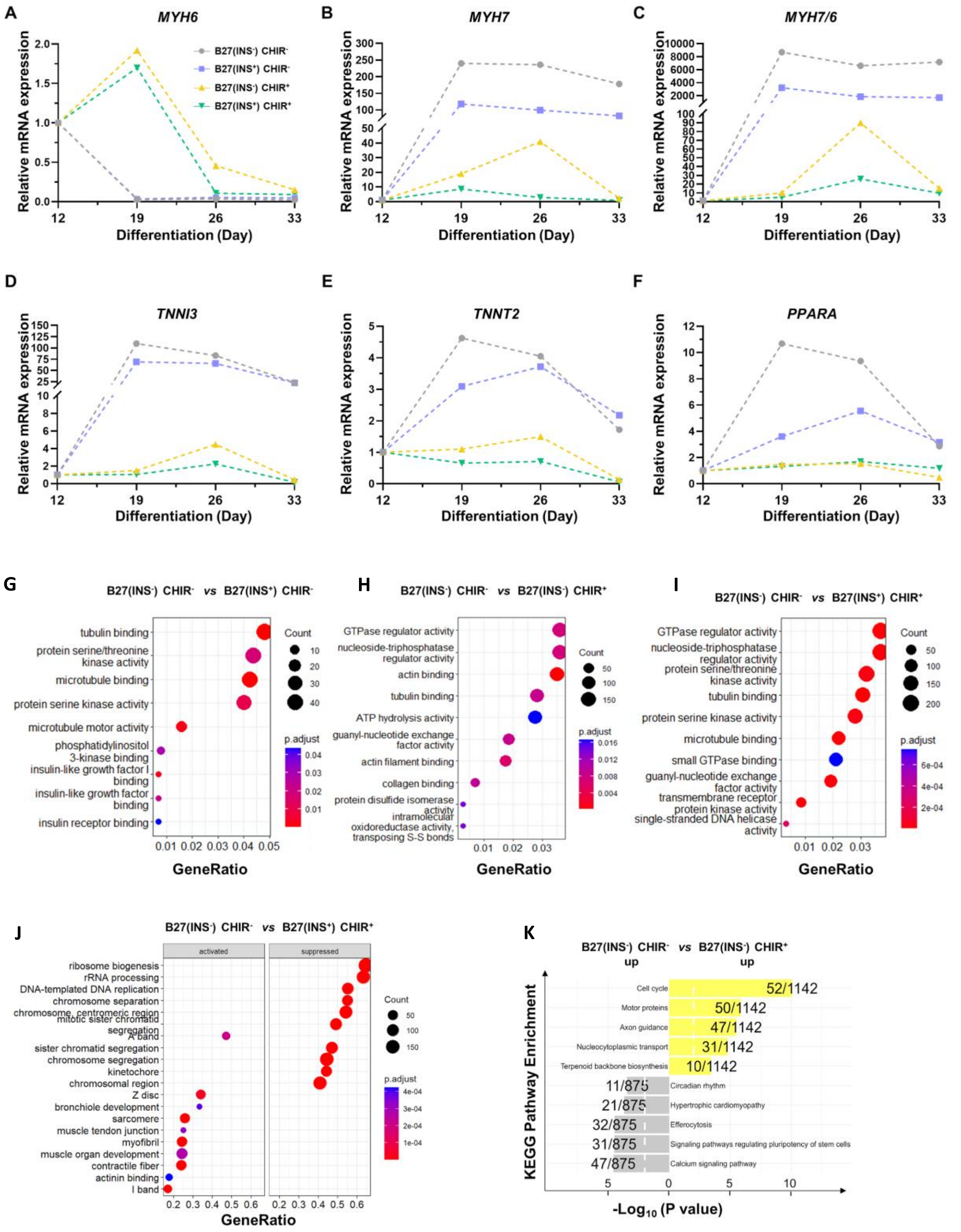

Figure S2. Expression of selected genes in hiPSC-CMs during prolonged treatment with Insulin/AKT and CHIR99021/Wnt activators (Related to Figure 2) and GO terms and KEGG pathway analysis.

(A-F) Gene expression, including *MYH6* (**A**), *MYH7* (**B**), *MYH7*/*MYH6* (**C**), *TNNI3* (**D**), *TNNT2* (**E**), *PPARA* (**F)**, displays a temporal pattern following treatment with insulin and/or CHIR99021 post-differentiation (Day 12). **(G-I**) Gene Ontology (GO) analysis of DEGs identified molecular functions

enriched in the designed conditions in hiPSC-CMs culture B27(INS$^+$) CHIR$^-$ (**G**), B27(INS$^-$) CHIR$^+$ (**H**), and B27(INS$^+$) CHIR$^+$ (**I**), comparing to B27(INS$^-$) CHIR$^-$, respectively. **GeneRatio**, the ratio of the number of DEGs to the total number of genes in the gene set. (**J**) GSEA-GO analysis of B27(INS$^-$) CHIR$^-$ versus B27(INS$^+$) CHIR$^+$. (**K**) Top KEGG pathways enrichment analysis between B27(INS$^-$) CHIR$^-$ and B27(INS$^-$) CHIR$^+$.

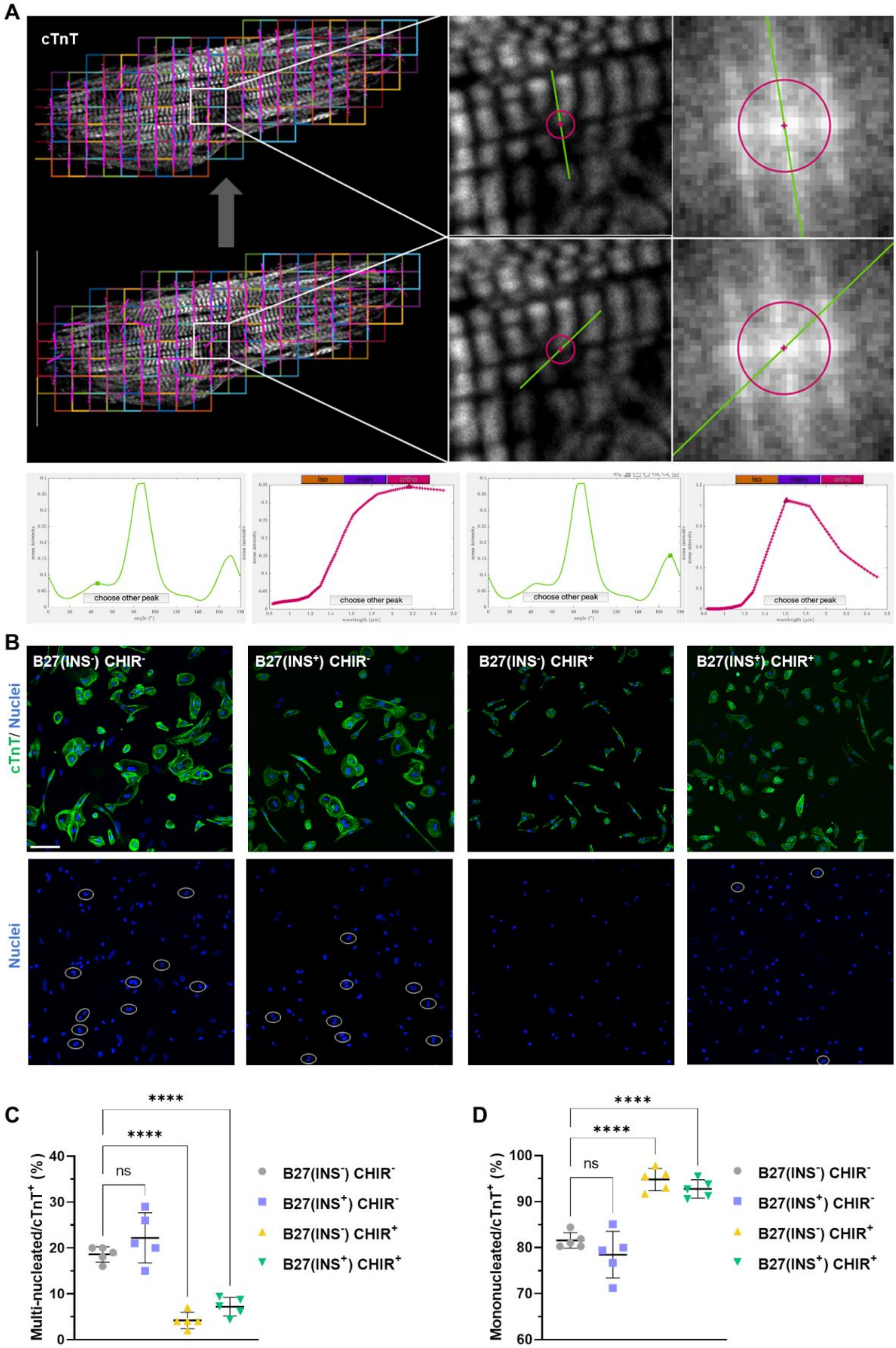
A
cTnT
choose other peak
iso
choose other peak
choose other peak
choose other peak
B
B27(INS-) CHIR-
B27(INS+) CHIR-
B27(INS-) CHIR+
B27(INS+) CHIR+
cTnT/ Nuclei
Nuclei
C
****
****
ns
Multi-nucleated/cTnT+ (%)
40
30
20
10
0
B27(INS-) CHIR-
B27(INS+) CHIR-
B27(INS-) CHIR+
B27(INS+) CHIR+
D
****
****
ns
Mononucleated/cTnT+ (%)
100
90
80
70
60
B27(INS-) CHIR-
B27(INS+) CHIR-
B27(INS-) CHIR+
B27(INS+) CHIR+

Figure S3. Insulin and CHIR99021 prompted the generation of mononuclear hiPSC-CMs. (**A**) Workflow for sarcomere quantification using MATLAB. (**B**) Representative immunofluorescence images of hiPSC-CMs show cTnT staining in red, with nuclei labeled in blue. Multi-nucleated cells are highlighted by circles as indicated. Scale bar=100 µm. (**C**) Quantification of the percentage of multi-nucleated hiPSC-CMs among total cTnT-positive hiPSC-CMs. (**D**) Quantification of the percentage of mononucleated hiPSC-CMs among total cTnT-positive hiPSC-CMs. Data are presented as mean ± SD and compared to B27(INS^-) $CHIR^-$ condition. N=5 independent cultures from SCVI-273 cell line. Not significant (ns), $p > 0.05$, ***$p < 0.001$, and ****$p < 0.0001$.

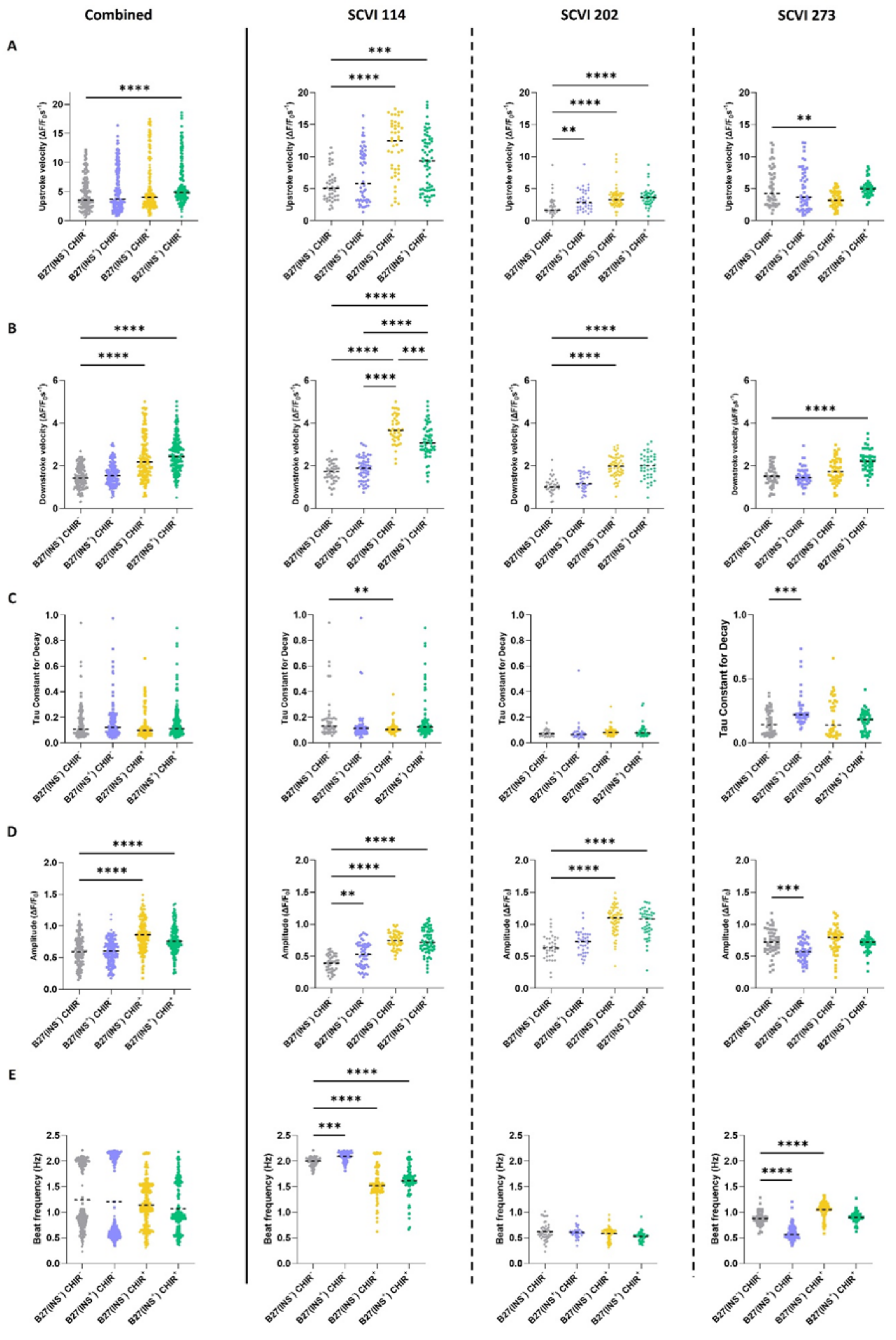
Combined
SCVI 114
SCVI 202
SCVI 273
A
B
C
D
E
Upstroke velocity (ΔF/F0s-1)
Downstroke velocity (ΔF/F0s-1)
Tau Constant for Decay
Amplitude (ΔF/F0)
Beat frequency (Hz)
B27(INS-) CHIR-
B27(INS+) CHIR-
B27(INS-) CHIR+
B27(INS+) CHIR+

Figure S4. Results of calcium measurements subdivided per cell line.
Scatterplots of upstroke velocity (**A**), downstroke velocity (**B**), tau constant for decay (**C**), calcium amplitude (**D**), and beating frequency (**E**) per cell line. Dotted line represents the median (for E the mean is displayed) and every dot is a measurement of one beating area (●= SCVI-114, ♦= SCVI-202, ■= SCVI-273). Comparisons are performed by Kruskal-Wallis test followed by Dunn's multiple comparisons test with B27(INS$^-$) CHIR$^-$ as the reference condition. Not significant, $p > 0.05$, *$p < 0.05$, **$p < 0.01$, ****$p < 0.0001$.

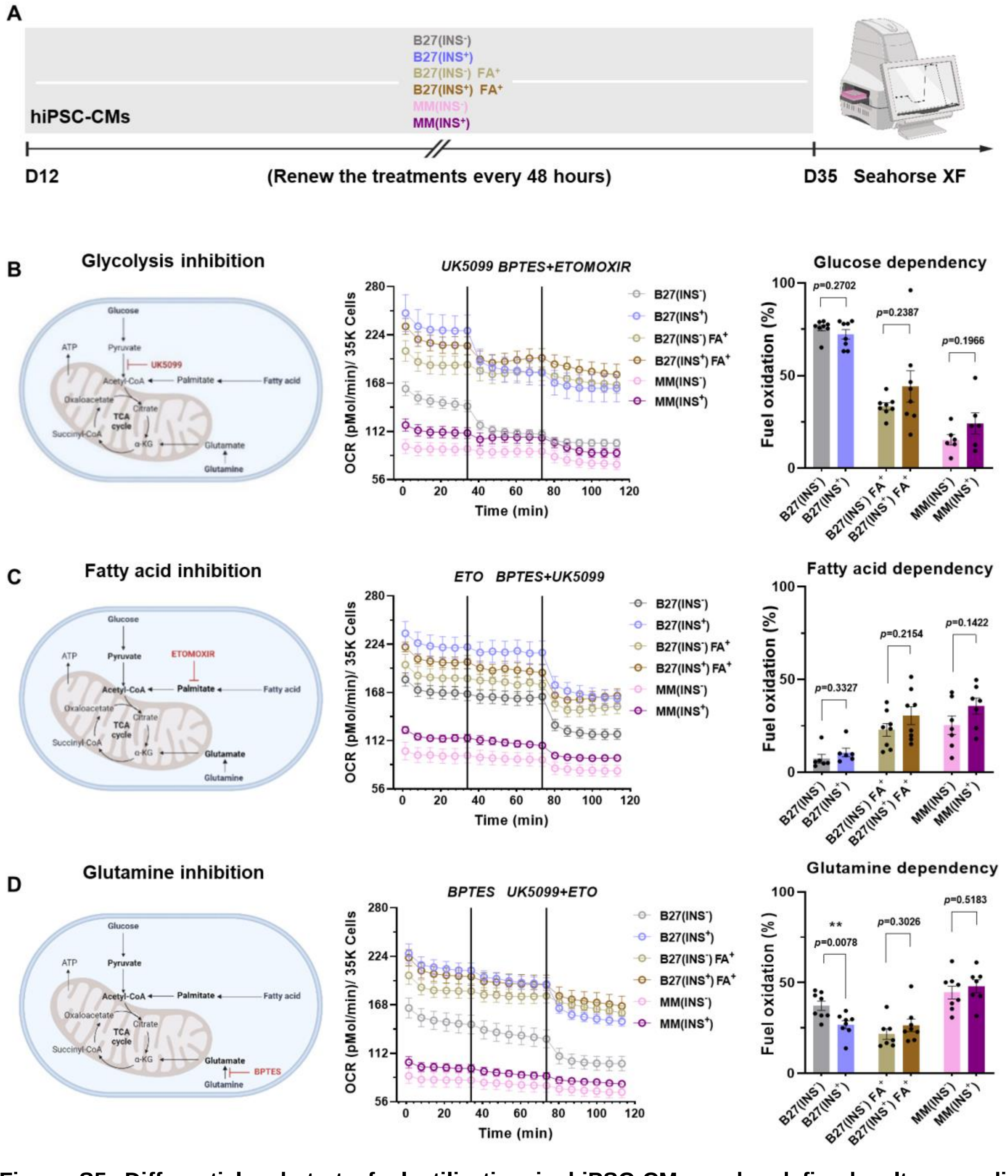

Figure S5. Differential substrate fuel utilization in hiPSC-CMs under defined culture media conditions modulated by insulin. (**A**) Schematic of the experimental timeline for Seahorse XF Mito Fuel-Flex assay involving hiPSC-CMs in culture separately with B27 with insulin (B27(INS$^+$)), B27 without insulin (B27(INS$^-$)), the addition of free fatty acid to B27(INS$^-$) or B27(INS$^+$), maturation medium (MM) with insulin (MM(INS$^+$)) or without insulin (MM(INS$^-$)). (**B-D**) Dependency on glucose (**B**), fatty acid (**C**), and glutamine (**D**) oxidation of hiPSC-CMs cultured in the indicated media. Created with BioRender.com. Data are presented as mean ± SEM. N≥ 6 independent cultures from 3 batches of SCVI-273 cell line. Significance assessed with unpaired Student's t-test between the groups, B27(INS$^-$) and B27(INS$^+$), B27(INS$^-$) FA$^+$ and B27(INS$^+$) FA$^+$, MM(INS$^-$) and MM(INS$^+$), respectively. **p< 0.01, p> 0.05, not significant (ns).

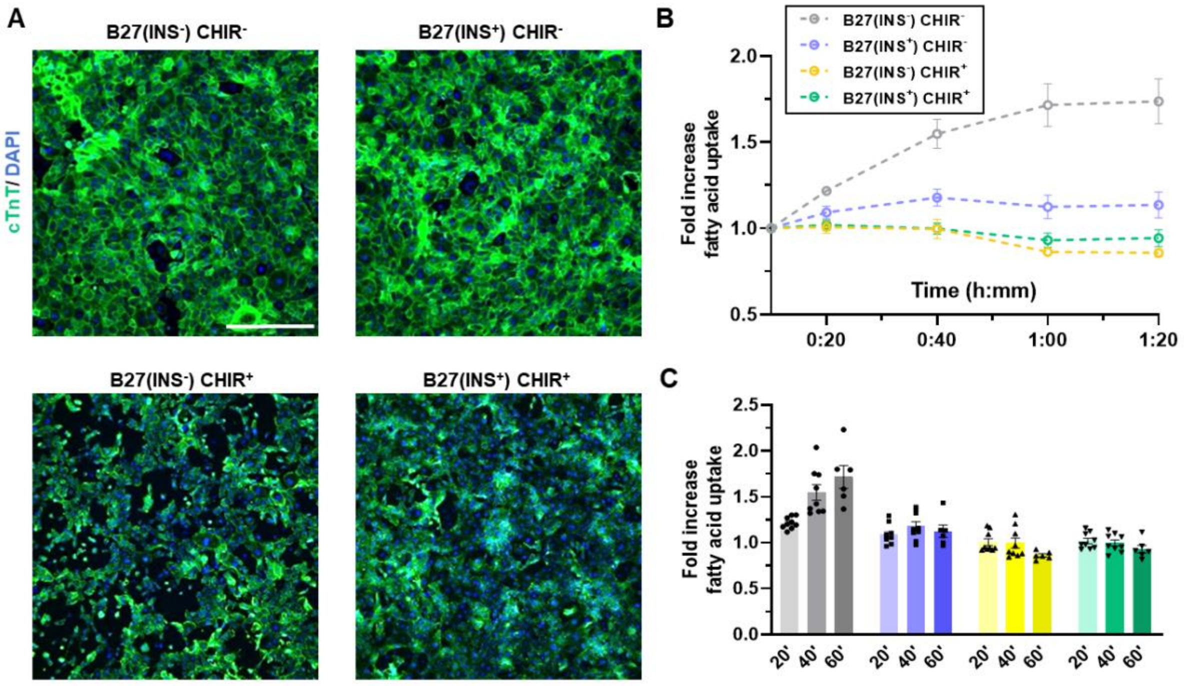

Figure S6. Fatty acid uptake in hiPSC-CMs treated with or without Insulin and/or CHIR99021. (Related to Figure 4). (**A**) Representative immunofluorescence images of hiPSC-CMs cultured under the indicated conditions with cTnT staining (green) and nuclei labeled with DAPI (blue). Scale bar=200 µm. (**B-C**) Fatty acid uptake fold increases measured every 20 minutes for cultured hiPSC-CMs **(B**). Highlight trends for fatty acid uptake increase in 20, 40, and 60 minutes for individual culture conditions (**C**). Data are normalized to the starting point, and presented as mean ± SEM. N≥ 6 independent cultures from 3 batches of SCVI-273 and SCVI-114 cell lines.

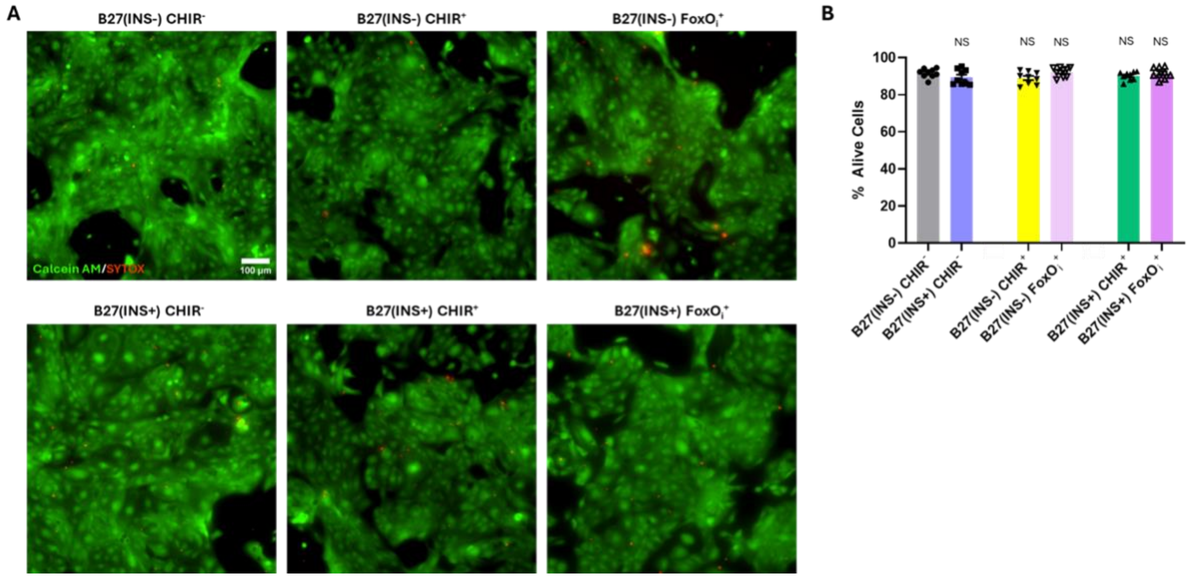

Figure S7. Administration of WNT activator (CHIR99021) and FOXO inhibitor (AS1842856) do not affect cell viability of immature hiPSC-CMs. (**A**) Live/Dead staining of immature SCVI-273 hiPSC-CMs after exposure for 24 hours to the designed culture conditions. Calcein AM indicates alive and viable cells (green), and SYTOX Deep Red staining indicates dead cells (red). (**B**) Quantification of percentage viable hiPSC-CMs per total cells for each condition. Data are presented as mean ± SEM. Experiment was performed in triplicate, every data point represents analysis of one image. After confirmation of normality using the D'Agostino & Pearson test, statistical significance was tested using one-way ANOVA followed by Tukey's multiple comparisons test relative to B27(INS-) CHIR$^-$.

Table S1. Primers

Function	Target	Sequence (5' --> 3')	Length	Tm
Myofibril maturation	*MYH6*	F: CGGTGCTTTTCAACCTCAAGG	21	61.7
		R: GGACTGGTTCTCCCGATCTGT	21	63.0
	MYH7	F: CTTTGCTGTTATTGCAGCCATT	22	60.0
		R: AGATGCCAACTTTCCTGTTGC	21	61.1
	TNNI1	F: CCGGAAGTCGAGAGAAAACCC	21	62.4
		R: TCAATGTCGTATCGCTCCTCA	21	60.7
	TNNI3	F: TTTGACCTTCGAGGCAAGTTT	21	60.1
		R: CCCGGTTTTCCTTCTCGGTG	20	62.8
	TNNT2	F: GGAGGAGTCCAAACCAAAGCC	21	62.9
		R:TCAAAGTCCACTCTCTCTCCATC	23	60.8
	MYL2	F: TTGGGCGAGTGAACGTGAAAA	21	62.7
		R: CCGAACGTAATCAGCCTTCAG	21	60.8
Cellular metabolism	*PPARA*	F: CGGTGACTTATCCTGTGGTCC	21	61.9
		R:CCGCAGATTCTACATTCGATGTT	23	60.7
	PPARG	F: GGGATCAGCTCCGTGGATCT	20	63.0
		R:TGCACTTTGGTACTCTTGAAGTT	23	60.0
	PPARGC1a	F: GCTTTCTGGGTGGACTCAAGT	21	62.0
		R: GAGGGCAATCCGTCTTCATCC	21	62.5
Wnt targets	*CTNNB1*	F: AAAGCGGCTGTTAGTCACTGG	21	62.6
		R: CGAGTCATTGCATACTGTCCAT	22	60.2
	AXIN2	F: CAACACCAGGCGGAACGAA	19	62.8
		R:GCCCAATAAGGAGTGTAAGGACT	23	61.4
	CCND2	F: CTGTCTCTGATCCGCAAGCAT	21	62.2
		R:GGTGGGTACATGGCAAACTTAAA	23	61.0
	LEF1	F: AGAACACCCCGATGACGGA	19	68.0
		R: GGCATCATTATGTACCCGGAAT	22	65.4
Housekeeping gene	*GAPDH*	F: GGAGCGAGATCCCTCCAAAAT	21	61.6
		R: GGCTGTTGTCATACTTCTCATGG	23	60.9

Table S2. Composition of metabolic substrates in media

Media / Compositions	RPMI 1640 basal medium		
	**B27 (+/- Insulin)**	**B27 (+/- Insulin)+ FA**	**MM (+/- Insulin)**
**Glucose**	11 mM	11 mM	3 mM
**L-Glutamine**	2 mM	2 mM	4 mM
**Fatty Acids**	Not disclosed	5 mg/mL	5 mg/mL
**L-carnitine**	Exists, but the conc. not disclosed	2 mM	2 mM

Table S3. Top 50 differentially expressed genes for the indicated comparisons

B27(INS-) CHIR- vs. B27(INS+) CHIR-

	Downregulated genes			
	Gene	*ENSEMBL*	*Log2FoldChange*	*Padj*
1	PDK4	ENSG00000004799	-2,86	1,47E-04
2	ENSG00000231170	ENSG00000231170	-2,20	1,88E-03
3	CCBE1	ENSG00000183287	-2,20	2,17E-04
4	PLSCR5	ENSG00000231213	-2,00	2,79E-06
5	GFRA1	ENSG00000151892	-1,49	5,89E-06
6	DLK1	ENSG00000185559	-1,45	1,64E-27
7	UNC5A	ENSG00000113763	-1,44	2,41E-02
8	PCARE	ENSG00000179270	-1,29	1,05E-10
9	LINC01122	ENSG00000233723	-1,28	3,89E-02
10	TDRD6	ENSG00000180113	-1,26	4,35E-02
11	MT1F	ENSG00000198417	-1,26	4,56E-02
12	PMEL	ENSG00000185664	-1,25	4,92E-12
13	POLN	ENSG00000130997	-1,23	3,98E-02
14	ENSG00000287109	ENSG00000287109	-1,22	2,42E-03
15	NDRG1	ENSG00000104419	-1,18	1,22E-08
16	FSTL5	ENSG00000168843	-1,18	1,84E-02
17	KLF5	ENSG00000102554	-1,17	3,95E-03
18	PIK3IP1	ENSG00000100100	-1,14	8,32E-12
19	C11orf21	ENSG00000110665	-1,13	5,04E-05
20	NEGR1	ENSG00000172260	-1,11	6,59E-03
21	CNTN5	ENSG00000149972	-1,10	2,20E-03
22	NKAIN2	ENSG00000188580	-1,07	3,27E-02
23	PTCHD1	ENSG00000165186	-1,04	5,25E-05
24	SCN11A	ENSG00000168356	-1,01	1,36E-02
25	IRS2	ENSG00000185950	-1,01	2,80E-14
26	ACOXL-AS1	ENSG00000204581	-0,99	2,23E-02
27	LRRN1	ENSG00000175928	-0,99	1,58E-03
28	KCNJ10	ENSG00000177807	-0,97	1,13E-02
29	SLC22A3	ENSG00000146477	-0,97	9,12E-03
30	LSAMP	ENSG00000185565	-0,96	7,00E-17
31	GPR35	ENSG00000178623	-0,95	4,87E-02
32	CNR1	ENSG00000118432	-0,94	4,61E-02
33	PKD1L2	ENSG00000166473	-0,94	1,19E-06
34	KLF3-AS1	ENSG00000231160	-0,92	4,00E-04
35	ACSM3	ENSG00000005187	-0,92	2,05E-02
36	PELI2	ENSG00000139946	-0,92	5,37E-08
37	ENSG00000287315	ENSG00000287315	-0,91	4,94E-05
38	ENSG00000261888	ENSG00000261888	-0,90	4,52E-02
39	ITGB8	ENSG00000105855	-0,89	1,99E-03
40	STAC	ENSG00000144681	-0,89	1,56E-02
41	GRM8	ENSG00000179603	-0,89	4,33E-02
42	ADGRG2	ENSG00000173698	-0,88	1,79E-03
43	UNC80	ENSG00000144406	-0,86	8,59E-03
44	ITGA9	ENSG00000144668	-0,86	3,82E-06
45	ENSG00000271155	ENSG00000271155	-0,86	6,69E-03
46	LINC00842	ENSG00000285294	-0,86	1,28E-02
47	SEMA3E	ENSG00000170381	-0,84	1,04E-04
48	SLITRK5	ENSG00000165300	-0,83	9,72E-03
49	AMPH	ENSG00000078053	-0,82	4,55E-02
50	WNT2B	ENSG00000134245	-0,82	4,52E-02

	Upregulated genes			
	Gene	*ENSEMBL*	*Log2FoldChange*	*Padj*
1	ELF3	ENSG00000163435	2,20	1,28E-04
2	ENG	ENSG00000106991	2,16	4,99E-02
3	FUT1	ENSG00000174951	1,82	1,56E-02
4	SERPINA1	ENSG00000197249	1,77	3,34E-03
5	NEFH	ENSG00000100285	1,77	4,04E-06
6	DNAI3	ENSG00000162643	1,75	8,75E-10
7	CCDC68	ENSG00000166510	1,75	2,88E-03
8	ODAM	ENSG00000109205	1,74	2,49E-03
9	LINC01182	ENSG00000250634	1,68	1,81E-02
10	STC1	ENSG00000159167	1,65	1,03E-04
11	AHNAK2	ENSG00000185567	1,64	8,35E-36
12	GJA5	ENSG00000265107	1,59	2,19E-10
13	RELT	ENSG00000054967	1,50	7,58E-06
14	TMEM63C	ENSG00000165548	1,44	1,32E-04
15	GPR3	ENSG00000181773	1,43	2,11E-02
16	CDH1	ENSG00000039068	1,40	1,52E-02
17	DYNLT5	ENSG00000152760	1,40	4,90E-04
18	SPOCK2	ENSG00000107742	1,39	1,65E-04
19	DUSP13	ENSG00000079393	1,36	2,41E-03
20	GRIN2B	ENSG00000273079	1,34	2,10E-03
21	SPAG4	ENSG00000061656	1,30	6,71E-03
22	DPF1	ENSG00000011332	1,29	7,90E-04
23	MOG	ENSG00000204655	1,24	9,79E-04
24	CNIH2	ENSG00000174871	1,22	1,63E-02
25	MMP1	ENSG00000196611	1,22	3,51E-02
26	HMGB1P1	ENSG00000124097	1,20	4,51E-02
27	HNRNPA1P27	ENSG00000233680	1,20	2,68E-02
28	MAT1A	ENSG00000151224	1,20	2,99E-02
29	XKRX	ENSG00000182489	1,19	1,11E-02
30	TRPV4	ENSG00000111199	1,19	7,50E-03
31	LAMB3	ENSG00000196878	1,19	3,15E-02
32	SNX10	ENSG00000086300	1,17	2,85E-04
33	RDM1	ENSG00000278023	1,17	4,78E-02
34	SLC6A20	ENSG00000163817	1,14	6,09E-06
35	ARHGAP45	ENSG00000180448	1,14	2,90E-07
36	CYTOR	ENSG00000222041	1,12	1,55E-02
37	SERTM2	ENSG00000260802	1,11	2,36E-07
38	NEIL3	ENSG00000109674	1,10	8,80E-05
39	DBNDD1	ENSG00000003249	1,10	1,84E-03
40	RNFT2	ENSG00000135119	1,10	3,96E-06
41	TUBB4A	ENSG00000104833	1,09	4,91E-03
42	ATP8B3	ENSG00000130270	1,09	2,06E-02
43	PLEKHG1	ENSG00000120278	1,08	6,48E-05
44	DDIT4	ENSG00000168209	1,08	2,93E-19
45	SREBF1	ENSG00000072310	1,06	5,74E-13
46	TM6SF1	ENSG00000136404	1,06	1,77E-02
47	L1TD1	ENSG00000240563	1,06	1,57E-02
48	GLT1D1	ENSG00000151948	1,04	2,23E-02
49	LINC00880	ENSG00000243629	1,03	1,07E-02
50	P2RX1	ENSG00000108405	1,02	4,52E-02

B27(INS-) CHIR- vs. B27(INS-) CHIR+

Downregulated genes				
	Gene	*ENSEMBL*	*Log2FoldChange*	*Padj*
1	XIRP2	ENSG00000163092	-4,59	5,28E-23
2	LMOD3	ENSG00000163380	-4,41	1,19E-57
3	PDE1A	ENSG00000115252	-4,41	2,56E-12
4	GPR22	ENSG00000172209	-4,31	4,22E-32
5	TCIM	ENSG00000176907	-4,16	1,58E-04
6	PSKH2	ENSG00000147613	-3,87	6,53E-05
7	BMP10	ENSG00000163217	-3,86	2,75E-37
8	ENSG00000230623	ENSG00000230623	-3,83	4,20E-04
9	NRAP	ENSG00000197893	-3,76	4,48E-04
10	SDAD1P1	ENSG00000228451	-3,61	5,33E-22
11	ASB11	ENSG00000165192	-3,53	1,66E-45
12	LINC01034	ENSG00000224933	-3,50	2,46E-04
13	ANKRD2	ENSG00000165887	-3,46	1,27E-03
14	SLC6A20	ENSG00000163817	-3,45	2,66E-12
15	C11orf21	ENSG00000110665	-3,44	7,99E-30
16	OR51E1	ENSG00000180785	-3,37	1,88E-03
17	ENSG00000249261	ENSG00000249261	-3,36	9,35E-04
18	NT5E	ENSG00000135318	-3,34	7,74E-05
19	KCNA5	ENSG00000130037	-3,34	5,16E-26
20	DMGDH	ENSG00000132837	-3,30	1,31E-05
21	KRT7	ENSG00000135480	-3,27	2,27E-11
22	PTGS2	ENSG00000073756	-3,24	2,55E-05
23	DLK1	ENSG00000185559	-3,21	5,14E-77
24	ENSG00000254202	ENSG00000254202	-3,21	1,40E-06
25	KLHL38	ENSG00000175946	-3,19	3,60E-48
26	KCNA7	ENSG00000104848	-3,16	6,33E-18
27	RGS8	ENSG00000135824	-3,15	1,57E-03
28	CFAP61	ENSG00000089101	-3,15	1,71E-06
29	ENSG00000254966	ENSG00000254966	-3,12	1,54E-04
30	HHATL	ENSG00000010282	-3,08	2,26E-09
31	ACTG2	ENSG00000163017	-3,08	1,38E-26
32	SLITRK6	ENSG00000184564	-3,07	3,73E-07
33	RASSF10	ENSG00000189431	-3,05	6,23E-04
34	SLC16A14	ENSG00000163053	-3,04	4,75E-29
35	ACTN3	ENSG00000248746	-3,03	2,91E-05
36	PPP1R3A	ENSG00000154415	-3,03	3,30E-06
37	ACOXL-AS1	ENSG00000204581	-2,98	1,79E-10
38	ENSG00000231170	ENSG00000231170	-2,96	7,05E-06
39	TTR	ENSG00000118271	-2,94	2,17E-04
40	SOX15	ENSG00000129194	-2,93	1,15E-08
41	NPIPB2	ENSG00000234719	-2,91	1,98E-06
42	SCT	ENSG00000070031	-2,90	4,16E-03
43	ENG	ENSG00000106991	-2,88	5,30E-06
44	CYSLTR1	ENSG00000173198	-2,84	3,40E-08
45	KLRD1	ENSG00000134539	-2,83	1,41E-04
46	LINC01449	ENSG00000224017	-2,83	8,99E-08
47	ATP1B4	ENSG00000101892	-2,80	1,22E-04
48	CACNA1S	ENSG00000081248	-2,77	4,94E-04
49	CTXND1	ENSG00000259417	-2,76	1,62E-13
50	DSCAML1	ENSG00000177103	-2,75	8,41E-05

Upregulated genes				
	Gene	*ENSEMBL*	*Log2FoldChange*	*Padj*
1	TMEM132C	ENSG00000181234	7,79	1,14E-29
2	GLDC	ENSG00000178445	6,44	7,26E-93
3	RSPO2	ENSG00000147655	5,01	4,54E-30
4	ISM1	ENSG00000101230	4,94	9,99E-08
5	SP5	ENSG00000204335	4,68	8,45E-32
6	LURAP1L	ENSG00000153714	4,58	1,20E-75
7	TAGLN3	ENSG00000144834	4,40	2,44E-06
8	UNC5D	ENSG00000156687	4,30	3,15E-130
9	LHX4	ENSG00000121454	4,10	3,34E-13
10	LINC02395	ENSG00000257771	4,06	1,32E-08
11	PDYN	ENSG00000101327	4,04	2,12E-11
12	CES1	ENSG00000198848	3,81	2,34E-05
13	ADAMTSL2	ENSG00000197859	3,72	5,60E-25
14	ENSG00000213981	ENSG00000213981	3,64	2,50E-04
15	ENSG00000278514	ENSG00000278514	3,59	3,24E-13
16	GRIN2B	ENSG00000273079	3,56	5,10E-33
17	LINC00269	ENSG00000215162	3,50	1,16E-04
18	ENSG00000285637	ENSG00000285637	3,44	3,97E-04
19	NEGR1	ENSG00000172260	3,42	5,22E-83
20	DCN	ENSG00000011465	3,42	2,86E-28
21	DIRAS2	ENSG00000165023	3,30	2,12E-52
22	RGCC	ENSG00000102760	3,27	2,18E-13
23	CMTM5	ENSG00000166091	3,21	8,53E-24
24	ENSG00000236924	ENSG00000236924	3,16	6,46E-05
25	OTULINL	ENSG00000145569	3,13	3,03E-28
26	PLPPR4	ENSG00000117600	3,08	8,35E-44
27	KCNG1	ENSG00000026559	3,06	4,56E-76
28	MEGF10	ENSG00000145794	3,05	5,00E-29
29	LYPD1	ENSG00000150551	3,00	1,71E-39
30	NDP	ENSG00000124479	3,00	1,07E-05
31	PALM3	ENSG00000187867	2,94	1,70E-09
32	NKD1	ENSG00000140807	2,91	4,93E-50
33	SLC25A48	ENSG00000145832	2,88	8,69E-23
34	ECEL1	ENSG00000171551	2,85	1,11E-35
35	TOX3	ENSG00000103460	2,85	6,33E-09
36	CD74	ENSG00000019582	2,83	5,93E-18
37	ENSG00000283235	ENSG00000283235	2,81	7,19E-03
38	DISC1FP1	ENSG00000261645	2,79	5,62E-05
39	WNT2	ENSG00000105989	2,78	1,10E-12
40	MYO10	ENSG00000145555	2,76	9,76E-49
41	CA8	ENSG00000178538	2,75	5,58E-59
42	ROBO2	ENSG00000185008	2,75	6,42E-21
43	HOXD9	ENSG00000128709	2,73	3,35E-04
44	HOXD8	ENSG00000175879	2,72	3,12E-08
45	ENSG00000280061	ENSG00000280061	2,71	1,88E-19
46	RP1	ENSG00000104237	2,64	1,62E-03
47	PALMD	ENSG00000099260	2,64	9,15E-10
48	AJAP1	ENSG00000196581	2,63	3,34E-07
49	RPRM	ENSG00000177519	2,63	7,97E-05
50	NRXN3	ENSG00000021645	2,58	2,33E-31

B27(INS-) CHIR- vs. B27(INS+) CHIR+

Downregulated genes

	Gene	*ENSEMBL*	*Log2FoldChange*	*Padj*
1	PDK4	ENSG00000004799	-4,64	5,18E-23
2	XIRP2	ENSG00000163092	-4,56	6,13E-30
3	BMP10	ENSG00000163217	-4,55	1,80E-59
4	KCNA5	ENSG00000130037	-4,40	1,03E-39
5	DNAAF3-AS1	ENSG00000267577	-4,32	1,09E-16
6	LINC00702	ENSG00000233117	-4,29	2,70E-19
7	SEMA5B	ENSG00000082684	-4,21	1,02E-55
8	ENSG00000231170	ENSG00000231170	-4,20	3,73E-07
9	ATP1B4	ENSG00000101892	-4,18	1,60E-05
10	LMOD3	ENSG00000163380	-4,07	1,21E-77
11	SOX15	ENSG00000129194	-3,93	1,98E-13
12	KRT80	ENSG00000167767	-3,93	1,04E-14
13	KLHL38	ENSG00000175946	-3,89	6,99E-88
14	GPR22	ENSG00000172209	-3,85	9,54E-42
15	ENSG00000286198	ENSG00000286198	-3,81	9,35E-12
16	CFAP61	ENSG00000089101	-3,80	5,83E-12
17	C11orf21	ENSG00000110665	-3,79	7,77E-45
18	SLITRK6	ENSG00000184564	-3,79	2,59E-10
19	PKD1L2	ENSG00000166473	-3,79	5,81E-48
20	ENSG00000275216	ENSG00000275216	-3,78	3,91E-06
21	ANKRD34C	ENSG00000235711	-3,78	3,10E-05
22	TCIM	ENSG00000176907	-3,76	1,25E-05
23	NT5E	ENSG00000135318	-3,70	2,67E-06
24	DLK1	ENSG00000185559	-3,69	9,03E-122
25	VIPR2	ENSG00000106018	-3,62	5,45E-25
26	ASB11	ENSG00000165192	-3,62	2,27E-57
27	LINC01449	ENSG00000224017	-3,61	5,37E-10
28	NRAP	ENSG00000197893	-3,58	1,43E-04
29	KCNA7	ENSG00000104848	-3,55	7,62E-26
30	SLC5A7	ENSG00000115665	-3,53	1,88E-15
31	SYNPR	ENSG00000163630	-3,49	9,09E-24
32	ACTG2	ENSG00000163017	-3,48	6,52E-36
33	ENSG00000285774	ENSG00000285774	-3,47	4,66E-04
34	HOPX	ENSG00000171476	-3,39	5,12E-88
35	TNS1-AS1	ENSG00000223923	-3,39	3,33E-61
36	ACOXL-AS1	ENSG00000204581	-3,37	1,73E-12
37	IL31RA	ENSG00000164509	-3,36	1,36E-07
38	CTXND1	ENSG00000259417	-3,34	3,78E-22
39	LINC02175	ENSG00000262155	-3,32	9,17E-45
40	SLC6A5	ENSG00000165970	-3,31	3,70E-06
41	DSCAML1	ENSG00000177103	-3,30	3,99E-06
42	SDAD1P1	ENSG00000228451	-3,30	1,54E-24
43	KRT7	ENSG00000135480	-3,29	2,35E-14
44	ANKRD2	ENSG00000165887	-3,28	5,50E-04
45	C5orf46	ENSG00000178776	-3,22	4,64E-08
46	KLHDC7B	ENSG00000130487	-3,21	1,11E-03
47	TCP11L2	ENSG00000166046	-3,19	1,84E-74
48	ENSG00000283128	ENSG00000283128	-3,19	9,18E-07
49	FGF1	ENSG00000113578	-3,18	1,07E-194
50	ENSG00000279619	ENSG00000279619	-3,17	4,74E-06

Upregulated genes

	Gene	*ENSEMBL*	*Log2FoldChange*	*Padj*
1	TMEM132C	ENSG00000181234	8,31	4,28E-34
2	GLDC	ENSG00000178445	6,69	2,18E-112
3	RSPO2	ENSG00000147655	5,48	6,63E-39
4	PDYN	ENSG00000101327	5,23	4,70E-21
5	TAGLN3	ENSG00000144834	5,22	1,31E-08
6	LINC00269	ENSG00000215162	5,14	1,53E-08
7	SP5	ENSG00000204335	5,08	9,53E-37
8	ISM1	ENSG00000101230	5,08	2,69E-08
9	CES1	ENSG00000198848	4,90	6,28E-08
10	GRIN2B	ENSG00000273079	4,76	1,05E-115
11	UNC5D	ENSG00000156687	4,61	1,31E-237
12	LURAP1L	ENSG00000153714	4,57	8,99E-86
13	LINC02395	ENSG00000257771	4,55	2,65E-12
14	NEFH	ENSG00000100285	4,45	3,52E-89
15	LHX4	ENSG00000121454	4,34	2,10E-15
16	WNT2	ENSG00000105989	4,32	1,10E-57
17	USP18	ENSG00000184979	4,25	3,41E-06
18	ENSG00000278514	ENSG00000278514	4,12	1,35E-18
19	ADAMTSL2	ENSG00000197859	4,10	1,50E-38
20	SPTA1	ENSG00000163554	4,00	1,49E-05
21	ENPP2	ENSG00000136960	3,93	1,57E-15
22	OTULINL	ENSG00000145569	3,90	2,14E-52
23	KCNG1	ENSG00000026559	3,79	1,39E-187
24	NKD1	ENSG00000140807	3,77	1,26E-74
25	CMTM5	ENSG00000166091	3,76	8,60E-46
26	DRD2	ENSG00000149295	3,68	1,55E-09
27	TLR3	ENSG00000164342	3,57	2,03E-06
28	PALM3	ENSG00000187867	3,57	3,48E-17
29	LINC02200	ENSG00000250358	3,57	3,09E-04
30	PLPPR4	ENSG00000117600	3,57	6,31E-96
31	CD74	ENSG00000019582	3,55	1,46E-36
32	CLEC1B	ENSG00000165682	3,53	1,83E-04
33	MEGF10	ENSG00000145794	3,51	1,09E-63
34	LINC01351	ENSG00000237457	3,47	1,69E-04
35	LINC01315	ENSG00000229891	3,42	4,55E-05
36	ENSG00000285637	ENSG00000285637	3,40	3,39E-04
37	DCN	ENSG00000011465	3,39	4,20E-38
38	ENSG00000213981	ENSG00000213981	3,37	5,60E-04
39	DPF1	ENSG00000011332	3,36	1,09E-65
40	HOXD8	ENSG00000175879	3,34	2,73E-13
41	NRXN3	ENSG00000021645	3,34	4,87E-80
42	ENSG00000236924	ENSG00000236924	3,30	1,77E-05
43	ENSG00000287311	ENSG00000287311	3,30	3,76E-04
44	TMEM100	ENSG00000166292	3,28	2,60E-09
45	SLC35D3	ENSG00000182747	3,27	1,98E-18
46	CA8	ENSG00000178538	3,22	6,28E-125
47	SLCO4A1	ENSG00000101187	3,22	6,76E-06
48	DIRAS2	ENSG00000165023	3,22	4,66E-29
49	ECEL1	ENSG00000171551	3,21	1,41E-82
50	GJA5	ENSG00000265107	3,20	3,54E-92

STAR Methods

Key resource table

REAGENT or RESOURCE	SOURCE	IDENTIFIER
**Antibodies**		
Cardiac Troponin T	Abcam	# Ab8295
Ki67	Abcam	# Ab15580
MLC2V	Abcam	# Ab48003
TOMM20	Abcam	# Ab186735
Donkey anti-Rabbit IgG (H+L) Alexa Fluor-488	Invitrogen	# A21206
Donkey anti-Rabbit IgG (H+L) Alexa Fluor-555	Invitrogen	# A31572
Donkey anti-Mouse IgG (H+L) Alexa Fluor-647	Invitrogen	# A31571
**Critical commercial assays**		
PSC Cryopreservation kit	ThermFisher	# A2644601
RNeasy Plus Mini Kit	QIAGEN	# 74134
Cell Line Optimization 4D-Nucleofector™ X Kit	Lonza	# V4XC-9064
Superscript cDNA synthesis kit	ThermFisher	# 11904018
iTaq SYBR Green Supermix	Bio-Rad	# 1725120
Free Fatty Acid Uptake Assay Kit	Abcam	# Ab176768
Seahorse XF Cell Mito Stress Test Kit	Agilent	# 103015-100
Seahorse XF Mito Fuel Flex Test Kit	Agilent	# 103260-100
Lipofectamine™ 3000 Transfection Kit	Invitrogen	# L3000001
Dual-Luciferase® Reporter Assay System	Promega	# E1910
**Biological samples**		
TCF reporter plasmid	Addgene	# M50
**Chemicals, peptides, and recombinant proteins**		
CHIR99021	Selleckchem	# S2924

C59	Selleckchem	# S737
Insulin solution	Sigma	# I9278
Matrigel® Growth Factor Reduced	Corning	# 354230
RPMI 1640	ThermoFisher	# 11875119
RPMI NO GLUCOSE	ThermoFisher	# 11879020
B27 supplement plus insulin	GIBCO	# 317504044
B27 supplement minus insulin	GIBCO	# A1895601
Essential 8 medium	GIBCO	# A1517001
DMEM/F12	GIBCO	# 21331020
DMEM, no glucose	GIBCO	# 11966025
KnockOut™ Serum Replacement	GIBCO	# 10828010
DAPI	ThermFisher	# 62248
DPBS (1X)	GIBCO	# 14190144
Bovine Serum Albumin	Sigma-Aldrich	# A7906
Hoechst 33342	ThermoFisher	# 62249
EDTA	ThermoFisher	# 15575020
TRYPLE 10X SEL	ThermoFisher	# A1217701
Revitacell Supplement (100x)	ThermoFisher	# A2644501
Rock inhibitor Y-27632	StemCell Technologies	# 72304
DMSO	Merck-Sigma	# D2650
Paraformaldehyde, 4% in PBS	ThermFisher	# 61899-AK
2-Phospho-L-ascorbic acid trisodium salt	Sigma-Aldrich	# 66170-10-3
Albumin	Sigma-Aldrich	# 70024-90-7
AlbuMAX™ I Lipid-Rich BSA	ThermFisher	# 11020021
Glucose	Sigma-Aldrich	# G7021
L-lactate	Sigma-Aldrich	# 71718
L-carnitine	Sigma-Aldrich	# C0283
Ascorbic acid	Sigma-Aldrich	# A8960
1x NEAA	ThermFisher	# 11140

Penicillin/Streptomycin	ThermoFisher	# 15140122
Fura-2, AM	Thermo Fisher	# F1221
Lipofectamine	Invitrogen	# L3000001
Luciferase substrate	Promega	# E1910
**Deposited data**		
Bulk RNA sequencing		GSE278598
**Experimental model: Cell lines**		
hiPSC line	Stanford Biobank	SCVI-273
hiPSC line	Stanford Biobank	SCVI-114
hiPSC line	Stanford Biobank	SCVI-111
hiPSC line	Stanford Biobank	SCVI-202
hiPSC line	UMG	TC1133-ACTN2-Citrine
**Oligonucleotide**		
**Primers**	This paper	Table S1
**Software and algorithms**		
GraphPad Prism 9	GraphPad Software	http://survey-smiles.com
Fiji-ImageJ	Shindelin et al., 2012	https://imagej.net/software/fiji/
Python 3.10.13	Python Software Foundation	https://www.python.org/
MATLAB_R2022b	MathWorks	https://www.mathworks.com/products/matlab.html
Ionwizard software	CytoCypher	https://www.ionoptix.com/products/software/ionwizard-core-and-analysis/
QuPath 0.5.0	Bankhead et al., 2017	https://github.com/qupath/qupath
BioRender	Science Suite Inc.	https://www.biorender.com/

R v4.0.2	R Core Team, 2021	https://www.r-project.org/
clusterProfiler	Yu et al., 2012	https://bioconductor.org/packages /release/bioc/html/clusterProfiler.html
DESeq2	Love et al., 2014	https://bioconductor.org/packages /release/bioc/html/DESeq2.html
PCAtools	Blighe and Lun, 2020	https://bioconductor.org/packages /release/bioc/html/PCAtools.html
ggplot2 package	R. A. M. Villanueva 2019	https://ggplot2.tidyverse.org

Resource availability

Lead contact

For additional information or requests regarding resources and reagents, please contact the corresponding author, Jan W. Buikema, j.w.buikema@amsterdamumc.nl

Materials availability

Please direct any additional information or requests for materials to the lead contact.

METHOD DETAILS

Immunofluorescence staining and confocal microscopy imaging

HiPSC-CMs were seeded at a density of approximately 6×10^4 cells/well into a μ-Plate 8-well chamber or 2×10^4 cells/well into a 96-well plate in this study. After experimental treatments, the cells were gently washed with PBS (1X) and fixed using 4% PFA for 10 minutes at room temperature. Following fixation, cells were permeabilized with 0.25% PBS-Triton for 10 minutes and then blocked using 3% BSA for 1 hour. Subsequently, the cells were incubated overnight at 4°C with primary antibodies (see key resource table for details). On the following day, the cells were incubated with corresponding fluorescently labeled secondary antibodies for

1 hour at room temperature. DAPI staining was applied for visualizing nuclear DNA. Imaging was conducted using either an AXR confocal microscope or an Eclipse Ti2 inverted microscope system (Nikon). Optionally, Z-stacks were acquired with a slice thickness of 0.22 µm under the 40X/0.95 NA objective. Subsequent image analysis and quantification were performed using NIS Elements software or ImageJ software.

For FUCCI CM live cell imaging: CQ1 Confocal Imaging Cytometer (Yokogawa) was used with a 20X/0.8 NA objective. Cells seeding density was $5x10^5$ cells/well for a 6-well cell culture plate. Images were quantified by Python and ImageJ.

Quantitative reverse transcription PCR (RT-qPCR)

HiPSC-CMs were harvested and lysed using TRIzol^{TM} reagent (Invitrogen). Total RNA extraction was conducted according to the manufacturer's instructions using the miRNeasy Mini kit (Qiagen). RNA concentration was measured by loading 1µL of extracted RNA samples on NanoDrop (Thermo Fisher Scientific). A total of 1000 ng of RNA was used for cDNA synthesis by using iScript cDNA synthesis kit (BioRad). Subsequently, the cDNA was diluted to a concentration of 5 ng/µL for subsequent RT-qPCR analysis. The RT-qPCR experiment was carried out using SYBR Green Supermix (Bio-Rad) on a Bio-Rad CFX96 device. For gene expression analysis, normalization was performed using the housekeeping gene glyceraldehyde-3-phosphate dehydrogenase (GAPDH) and calculated by the $2^{-\Delta\Delta Ct}$ method (Livak & Schmittgen, 2001).

Contractility measurements and calcium transient analysis

All contractility and calcium imaging experiments were performed on spontaneously beating hiPSC-CMs. To this end, d12 hiPSC-CMs were cultured under the following conditions: B27(INS$^-$) CHIR$^-$, B27(INS$^+$) CHIR$^-$, B27(INS$^-$) CHIR$^+$, and B27(INS$^+$) INS$^+$ for 4 days. Subsequently, the cells were seeded at a concentration of $4x10^5$ cells/well on a 24-well black 14 mm plate (IBIDI, 82421) coated with Matrigel (Corning) for another 3 days, with continuous treatment under the defined conditions as described above. Contractility and calcium transient measurements followed previously described methods (Dinani et al., 2023), with details modified and adapted for this study. Contractility measurements were conducted using the Cytocypher Multicell High Throughput System (Cytocypher BV (Amsterdam, The Netherlands), IonOptix Corporation (Westwood, MA, United States) when the cells were grown and treated under the aforementioned conditions. Spontaneously beating areas from monolayer cells were randomly selected and measured for 10 seconds per well across 12 regions for a total of 10 minutes. The data were collected using Transient Analysis Tool software (Cytocypher BV), and analyzed according to the following calculations: Contraction time = Time to peak – Time to peak 10. Relaxation time = Time to baseline 80 – Time to peak.

%Shortening = Peak height/ Baseline. For calcium transient measurements, Tyrode solution with a 1 mM Calcium concentration was prepared by thawing a 10 mL 10x Tyrode stock. To this stock, 80 mL MilliQ water, 55 mg sodium pyruvate, and 100 µL $CaCl_2$ were added. The pH was adjusted to 7.4 at 37°C. The medium in the wells was replaced with warm Tyrode solution at 37°C and incubated for 15 minutes. Fura-2-AM was diluted to a concentration of 2 µM by adding 2 µL per milliliter. Subsequently, the Tyrode solution was replaced with Fura-2-AM-containing Tyrode and incubated for 15 minutes. This was followed by replacement with normal Tyrode and an additional incubation for 5 minutes. Finally, the Tyrode was refreshed once, and the plate was measured in the Cytocypher (IonOptix) for calcium measurements. Spontaneously beating areas from monolayer cells were randomly selected and measured for 10 seconds per well across 12 regions, totaling 10 minutes. Data were collected using Transient Analysis Tool software (Cytocypher BV) and analyzed for ΔF/F0.

Seahorse XF Mito stress assay

To evaluate the effect of the CHIR99021 and insulin on mitochondrial respiration, Seahorse XF Mito-stress assay was performed according to manufacturer's instructions. HiPSC-CMs were independently cultured for 7 days in B27(INS^-) $CHIR^-$, B27(INS^+) $CHIR^-$, B27(INS^-) $CHIR^+$, and B27(INS^-) $CHIR^+$. 3-5 days prior to the experiment cells were replated into the Seahorse XF96-well cell culture microplate (Agilent) coated with Matrigel (Corning). On the day of the experiment, media were replaced by RPMI 1640 basal medium supplemented with 2 mM glutamine, 10 mM glucose, and 1 mM sodium pyruvate. To normalize the oxygen consumption rate (OCR) values to the actual cell number, cells were incubated with Hoechst (Thermo Fisher Scientific) for live cell imaging, and cell number was counted by a cell analyzer. Subsequently, mitochondrial stress test conditions were assessed according to manufacturer's instructions of the Seahorse XF Cell Mito Stress Test Kit (Agilent) using Seahorse XF96 platform by injecting the following inhibitors in sequence: Oligomycin (2.5 µM), FCCP (carbonyl cyanide-4 (trifluoromethoxy) phenylhydrazone, 2 µM), and Rotenone/antimycin A (2.5 µM). Parameters, including basal respiration, ATP-linked respiration, proton leak, and respiratory capacity, were calculated based on the changes in the OCR upon injection of the beforehand mentioned inhibitors.

Seahorse XF Mito Fuel Flex assay

To assess the effect of insulin on the substrate metabolism dependency of hiPSC-CMs in different defined culture media, Fuel-Flex assay was performed according to manufacturer's instructions. HiPSC-CMs were independently cultured in B27(INS^-) and B27(INS^+), B27(INS^-) FA^+ and B27(INS^+) FA^+, maturation medium minus insulin (MM(INS^-)) and maturation medium plus insulin (MM(INS^+)) until day 35 post-differentiation. 3-5 days prior to the experiment, cells were replated into the Seahorse XFe96 Cell Culture Microplates (Agilent)

coated with Matrigel (Corning). On the day of the experiment, media were replaced by RPMI 1640 basal medium (Thermo Fisher Scientific) supplemented with 2 mM glutamine, 10 mM glucose, and 1 mM sodium pyruvate. To normalize the OCR values to the actual cell number, cells were incubated with Hoechst (Thermo Fisher Scientific) for live cell imaging. Subsequently, cell number was counted by a cell analyzer shortly before the seahorse experiments. Three main substrate oxidation pathways were analyzed according to manufacturer's instructions from Seahorse XF Mito Fuel Flex Test Kit (Agilent). The inhibitors from the kit were injected sequentially for the test: A) UK5099, a pyruvate carrier inhibitor, was used to assess the mitochondrial glycolysis dependency, B) Etomoxir, an inhibitor of long chain fatty acid metabolism, and C) BPTES, an inhibitor of glutamine oxidation pathway. Mitochondrial dependency to each substrate was calculated using the following formula: Substrate fuel dependency = ((baseline OCR – target inhibitor OCR)/ (baseline OCR - all inhibitors OCR))*100.

Fatty acid uptake assay

Four days prior to the assay, the Matrigel-coated 96-well black plate with clear bottom was seeded with $6x10^4$ hiPSC-CMs. The assessment of fatty acid was carried out using the Free Fatty Acid Uptake Assay Kit (Abcam) following the manufacturer's instruction. Briefly, after being washed thrice with serum-free media, the cells were pre-incubated in serum-free medium for 2 hours. A baseline measurement was conducted before introducing the fluorescent fatty acid mixture, followed by measurements every 10 minutes after the addition using a microplate fluorescence reader (Greiner Bio-One) at 485/528 nm performed in Spectramax ID3 (Molecular Devices).

Bulk RNA-seq analysis

D12 hiPSC-CMs were cultured, respectively, under the defined conditions, including B27(INS$^-$) CHIR$^-$, B27(INS$^+$) CHIR$^-$, B27(INS$^-$) CHIR$^+$, and B27(INS$^-$) CHIR$^+$, for 3 days. Subsequently, total RNA was extracted following the described method as above. Library preparations were carried out using the NEBNext® Ultra RNA Library Prep Kit for Illumina® (New England Biolabs), and the prepared libraries underwent sequencing on a Hiseq 4000 platform (Novogene). The RNA-seq reads were aligned to the human reference genome hg19. Differential expression analysis of genes (DEGs) between groups (adjusted p value < 0.01) was conducted by DESeq using RStudio. Functional enrichment analysis was performed by Bioconductor packages using RStudio.

Luciferase-based LEF/TCF activity analysis

β-Catenin/TCF activity was evaluated using the luciferase-based LEF/TCF reporter, TOPFlash plasmid (Addgene). For early-stage cells, D12 hiPSC-CMs were cultured in RPMI 1640 basal medium-based conditions: B27(INS^-) $CHIR^-$, B27(INS^+) $CHIR^-$, B27(INS^-) $CHIR^+$, and B27(INS^-) $CHIR^+$ for 4 days (D16). For late-stage cells, D12 hiPSC-CMs were cultured in RPMI 1640 basal media plus insulin until D40, cells were switched to the conditions: B27(INS^-) $CHIR^-$, B27(INS^+) $CHIR^-$, B27(INS^-) $CHIR^+$, and B27(INS^-) $CHIR^+$ for 4 days (D44). The D16 and D44 cells were, respectively, replated into 96-well clear black bottom plates (Greiner Bio-One) with a cell density 1×10^5 cells/well. The following day, transfection was performed using Lipofectamine™ 3000 Transfection Reagent (Invitrogen) according to the manufacturer's protocol, with the Renilla luciferase gene as an internal control. After 36 h, cells were lysed in standard 1X lysis buffer (Promega) for 30 min at room temperature, and the cell lysates were subjected for both firefly and Renilla luciferase activity using the Dual-Luciferase Reporter assay kit (Promega) following the manufacturer's instructions. Fluorescence was measured using SoftMax® Pro (Molecular Devices), Renilla luciferase was used as a control reporter for normalization. LEF/TCF activity from each culture condition was calculated and finally presented relative to B27(INS^-) $CHIR^-$.
