# Supplementary material for: Glycogen synthase kinase-3 inhibition and insulin enhance proliferation and inhibit maturation of human iPSC-derived cardiomyocytes via TCF and FOXO signaling
Source: Stem Cell Reports. 2024 Dec 5;20(1):102371. doi: 10.1016/j.stemcr.2024.11.001 (PMC11784517; doi:10.1016/j.stemcr.2024.11.001)
Supplement: Document S2. Article plus supplemental information [file mmc2.pdf]

Stem Cell Reports
Article
OPEN ACCESS

Glycogen synthase kinase-3 inhibition and insulin enhance proliferation and inhibit maturation of human iPSC-derived cardiomyocytes via TCF and FOXO signaling

Qianliang Yuan,[1] Devin Verbueken,[1,2] Rafeeh Dinani,[1] Rosa Kim,[3,4] Eric Schoger,[3,4] Chloé D. Morsink,[1] Shamim Amiri Simkooei,[1] Luuk J.M. Kemna,[1,5] Jesper Hjortnaes,[5,6] Diederik W.D. Kuster,[1] Reinier A. Boon,[1] Laura Cecilia Zelarayan,[3,4,7] Jolanda van der Velden,[1] and Jan W. Buikema[1,2,8,*]
[1]Amsterdam Cardiovascular Sciences, Department of Physiology, Amsterdam University Medical Center, VU University, Amsterdam, the Netherlands
[2]Amsterdam Heart Center, Department of Cardiology, Amsterdam University Medical Center, Amsterdam, the Netherlands
[3]DZHK (German Centre for Cardiovascular Research) Partner Site Göttingen, Göttingen, Germany
[4]Institute of Pharmacology and Toxicology, University Medical Center Göttingen, Göttingen, Germany
[5]Laboratory of Experimental Cardiology, Department of Cardiology, Leiden University Medical Center, Leiden, the Netherlands
[6]Heart Lung Center, Department of Cardiothoracic Surgery, Leiden University Medical Center, Leiden, the Netherlands
[7]Justus Liebig University, Medical Clinic I, Department of Cardiology and Angiology, Giessen, Germany
[8]Lead contact
*Correspondence: j.w.buikema@amsterdamumc.nl
https://doi.org/10.1016/j.stemcr.2024.11.001

SUMMARY

Embryonic signaling pathways exert stage-specific effects during cardiac development, yet the precise signals for proliferation or maturation remain elusive. To uncover the cues for proliferation, we performed a combinatory cell-cycle screen for insulin and glycogen synthase kinase-3 (GSK3) inhibition in spontaneously beating human induced pluripotent stem cell-derived cardiomyocytes (hiPSC-CMs). Our analysis for proliferation, and subsequential downstream sarcomere development, gene expression analysis, and molecular interventions identified a temporal interplay between insulin/Akt/FOXO and CHIR99021/Wnt/GSK3/TCF signaling. Combined pathway activation led to proliferation of immature hiPSC-CMs with low sarcomere and mitochondria content, while, in the absence of pathway activators, cardiomyocytes rapidly exited the cell cycle and fetched higher organization of sarcomeres and mitochondria. Our data demonstrate two important pathways, which enhance proliferation and inhibit maturation, and provide molecular mechanistic understanding of these cell fate decisions in immature hiPSC-CMs.

INTRODUCTION

The lack of understanding about true regeneration of the human heart is reflected in the current medical therapies for heart failure that do not aim to cure the underlying deficit of functional cardiomyocytes (Galdos et al., 2017; Lazar et al., 2017; Ouwerkerk et al., 2023). Whereas cell turnover in the adult heart is extremely low, during development embryonic signaling pathways regulate the robust increase in myocardial mass through cardiomyocyte duplication. Orchestration of these cell fate decisions require temporal and stage-specific specification and growth of the various myocardial compartments. This involves a tight regulation of the local cardiomyocyte proliferation and is directed by a network of signaling pathways, including Neuregulin1/ErbB2/4 (Bersell et al., 2009; D'Uva et al., 2015; Grego-Bessa et al., 2023), bone morphogenic proteins (BMPs) (Klaus et al., 2007), insulin growth factor (IGF)/phosphatidylinositol 3-kinase (PI3K)-AKT (Engels et al., 2014), Hippo-YAP (Heallen et al., 2011; Lin et al., 2015; von Gise et al., 2012; Xin et al., 2013), and NOTCH (Collesi et al., 2008; Grego-Bessa et al., 2007).

Remarkably, within the developing ventricular compartment, differential proliferation rates are observed within the compacted, trabecular, and septal zones. The proliferation rates of immature cardiomyocytes in the compacted myocardium gradually decline following specification and maturation (Buikema et al., 2013; Ye et al., 2015). Differentiation coincides with multiple isoform switches of genes that regulate the expression of proteins essential for the contractile apparatus, culminating in their functional maturity (Miyata et al., 2000; Reiser et al., 2001), In parallel, these sarcomeric isotype switches coincide with a metabolic preference for fatty acids as the substrate for adenosine triphosphate (ATP) production (Lopaschuk et al., 1991). These functional and metabolic transitions in ventricular cardiomyocytes are driven by a demand for increased cardiac output of the growing body.

We and others have previously shown that during mitosis cardiomyocytes disassemble all sarcomeres before proceeding to cytokinesis, a phenomenon inversely related to contractility (Buikema et al., 2020; Yuan et al., 2022). Moreover, we and others have shown that the canonical Wnt/β-catenin signaling pathway plays an essential role in regulating the cell fate decisions such as required for cardiogenesis and cardiac development. Notably, the canonical Wnt/β-catenin signaling pathway exerts a biphasic effect on mesoderm development versus cardiac

© 2024 The Authors.
Published by Elsevier Inc. on behalf of International Society for Stem Cell Research.
This is an open access article under the CC BY license (http://creativecommons.org/licenses/by/4.0/).

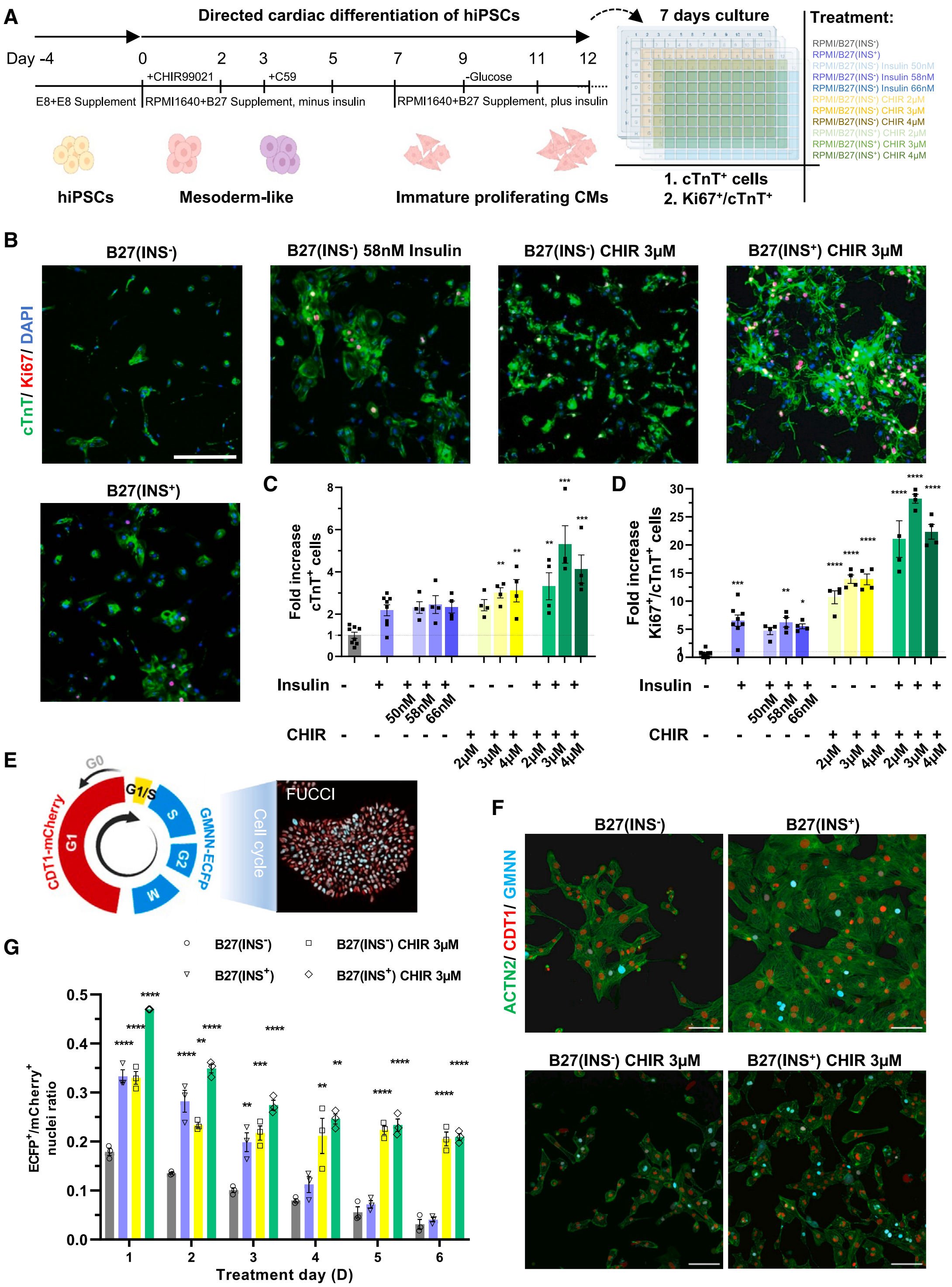

Figure 1. Combinatorial screen of insulin and GSK3β inhibitor CHIR99021 for hiPSC-CM proliferation

(A) Schematic of combinatory screen setup for insulin and CHIR99021 (CHIR) in hiPSC-CMs.

(B) Representative immunofluorescence images of hiPSC-CMs for the optimized concentrations of insulin (58 nM–340 ng/mL) and/or CHIR (3.0 μM). Cells were stained for cTnT (green), Ki67 (red), and nuclei (DAPI, blue). Scale bar, 200 μm.

(C) Graph displaying fold increase in $cTnT^{+}$ cells normalized to $B27(INS^{-})$.

(D) Graph displaying fold increase in percentage of $Ki67^{+}$/cTnT+ cells normalized to $B27(INS^{-})$.

(legend continued on next page)

specification and growth, which is incorporated in the robust cardiac differentiation of human induced pluripotent stem cells (hiPSCs) (Lian et al., 2012, 2013) and proliferation of immature hiPSC-derived cardiomyocytes (hiPSC-CMs) (Buikema et al., 2020; Mills et al., 2019; Sharma et al., 2018; Titmarsh et al., 2016; Uosaki et al., 2013).

Yet, it remains unknown what cues facilitate the switch between proliferation and maturation in immature hiPSC-CMs (Fetterman et al., 2024). Here, we study the contribution of the CHIR99021 (CHIR) small molecule via the Wnt/glycogen synthase kinase (GSK)3β/β-catenin/T-cell factor (TCF) axis and insulin via the insulin/PI3K-AKT/Forkhead box O (FOXO) route on the stage-specific effects for proliferation and maturation in immature hiPSC-CMs. In a combinatory screen we found that both insulin and CHIR had effects on promoting proliferation of immature hiPSC-CMs, whereas the absence of these pathway activators led to rapid cell-cycle exit and metabolic and structural maturation of hiPSC-CMs. These findings provide novel insights into the molecular signaling pathways that control the interplay between proliferation and maturation of cardiomyocytes during heart development.

RESULTS

Insulin and CHIR99021 are important cues for proliferation of immature hiPSC-CMs

One of the widely used methods for hiPSC-CM differentiation and culture consists of RPMI 1640 medium supplemented with B27 components containing an estimated final concentration of 58 nM (340 ng/mL) of insulin (Lian et al., 2012; Decourtye et al., 2018). Previous research demonstrated that the addition of CHIR in 2–5 μM to the culture media resulted in significant long-term expansion of hiPSC-CMs (Buikema et al., 2020; Quaife-Ryan et al., 2020). Remarkably, addition of CHIR to the chemically defined media consisting of three components (CDM3) did not result in a long-lasting expansion of hiPSC-CMs (data not shown). We set up a combinatory screen for a gradient of insulin and CHIR concentrations to assess the effects on hiPSC-CM proliferation (Figures 1A and S1). Our screen showed that the combination of 58 nM (340 ng/mL) insulin and 3.0 μM CHIR resulted in optimal proliferation of hiPSC-CMs (Figures 1B and S1D). Moreover, when both insulin and CHIR compounds were withdrawn from the RPMI 1640 with B27 supplement (minus insulin) media, the hiPSC-CMs that were sparsely seeded exhibited extremely low proliferation rates (1.48% ± 0.18%) versus high average proliferation rates (38.45% ± 3.86%) in cells stimulated with both insulin and CHIR, such as quantified by the fraction of Ki67- and Troponin T-positive cells (Figures 1C and 1D). In contrast, stimulating hiPSC-CMs with insulin or CHIR alone only increased proliferation by 5- to 10-fold (Figure 1D). We further confirmed hiPSC-CM cell-cycle activity upon CHIR and insulin exposure in a double-transgenic FUCCI hiPSC cell cycle reporter line (Figure 1E). Culturing cells in CHIR-containing media increased the percentage of S/G2-phase cardiomyocytes ($ECFP^+$ cells), and supplementation of insulin enhanced the cell-cycle activation in the first days of treatment (Figures 1F, 1G, and S1E). We conclude that in our previously described expansion media, consisting of multiple factors (B27) and CHIR, the insulin and CHIR form the two essential molecular cues for hiPSC-CM proliferation.

No exposure to insulin and CHIR99021 results in differentiation of immature hiPSC-CMs

Previous studies in hiPSCs have shown that the cell-cycle pathways act as a deterministic restriction on the pluripotent state of stem cells. Pluripotency control is connected to the cell-cycle machinery, where S and G2 phase-specific pathways deterministically restrict pluripotent state dissolution (Gonzales et al., 2015). In this process, pathways such as PI3K/AKT, transforming growth factor β, MEK, and FGF in G1 phase potentially permit the initiation of differentiation (Gonzales et al., 2015; Liu et al., 2019). Based on our previously described hiPSC-CM expansion media (Buikema et al., 2020; Maas et al., 2021), we identified two main pathway activators for cell division in Figure 1. To further investigate the impact of CHIR and insulin on the maturity of hiPSC-CMs, we established four distinct treatment groups based on the presence or absence of CHIR (3 μM) and/or insulin, denoted as "B27(INS^-) $CHIR^-$," "B27(INS^+) $CHIR^-$," "B27(INS^-) $CHIR^+$," and "B27(INS^+) $CHIR^+$." Using quantitative reverse-transcription PCR

(E) Schematic of FUCCI cell-cycle reporter system with CDT1-mCherry and GMNN-ECFP.

(F) Representative triple fluorescent live-cell imaging hiPSC-CMs expressed CDT1-mCherry, GMNN-eCFP, and ACTN2-eGFP for indicated culture conditions. Scale bar, 100 μm.

(G) Graph displaying ratios of CDT1-mCherry-positive cells (nuclei) and GMNN-ECFP-positive cells (nuclei).

(B–D) Data are presented as mean ± SEM. $n \geq 4$ independent cultures from three batches of SCVI-273 cell line. Data are normalized to B27(INS^-) condition for fold increase of cell number in $cTnT^+$ and/or $Ki67^+$ hiPSC-CMs. (F and G) $N = 3$ independent experiments from the TC1133-ACTN2-Citrine-FUCCI cell line were analyzed in 9 replicates per condition. (C, D, and G) Statistical analysis by one-way ANOVA followed by Dunn's multiple comparisons test compared to the B27(INS^-) condition (. $^{**}p < 0.01$, $^{***}p < 0.001$, and $^{****}p < 0.0001$.

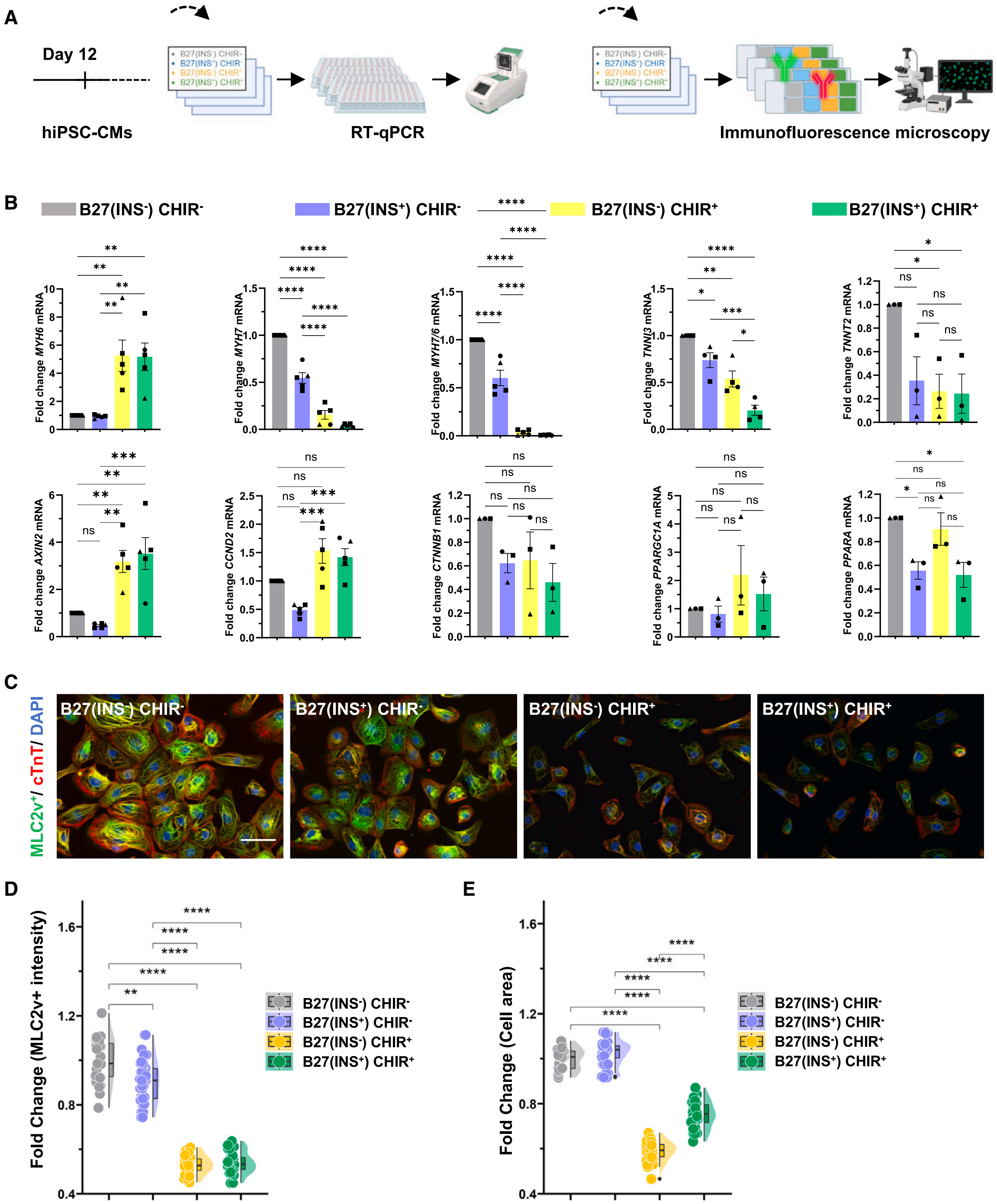

Figure 2. Molecular characterization of immature hiPSC-CMs exposed to combinations of insulin and CHIR99021

(A) Schematic of the experimental setup (B) RT-qPCR analysis of mRNA expression of the indicated sarcomere, cell cycle, Wnt target, and glucose homeostasis genes. Data from each independent biological experiment are normalized to B27(INS$^-$) CHIR$^-$, as indicated by the dots: ▲ = SCVI-111 ● = SCVI-114 ■ = SCVI-273.

(C) Representative immunofluorescence images of hiPSC-CMs for the indicated treatments. Images show cTnT (red), MLC2v (green), and nuclei (DAPI, blue). Scale bar, 50 μm.

(legend continued on next page)

(RT-qPCR) and immunofluorescence assays, we examined mRNA expression levels associated with cardiomyocyte proliferation or maturation (Figures 2A and S2A–S2F). We found a 5-fold higher expression of the embryonic isoform of myosin heavy chain (*MYH6*) in hiPSC-CMs treated with CHIR when compared to only insulin or no stimuli present in the media (Figure 2B). In hiPSC-CMs treated with CHIR, we confirmed activated Wnt target gene expression by *AXIN2* and observed increased expression of the cell-cycle gene *CCND2* (Figure 2B). Interestingly, we found that withdrawal of CHIR and/or insulin gradually led to increased expression of the adult isoform of myosin heavy chain (*MYH7*), the mature/immature myosin heavy chain ratio (*MYH7/6*), and the Troponin I (*TNNI3*) gene. Stable transcriptional activity was found for β-catenin (*CTNNB1*) or the PPAR transcription factors involved in energy homeostasis (Figure 2B). To evaluate the expression of sarcomere proteins after exposure to the defined four treatment conditions, we stained for one cardiac marker, cardiac troponin T (cTnT), and one cardiomyocyte maturation marker, ventricular specific myosin light chain 2v (MLC2v). Notably, we found that MLC2v signal intensity was most highly expressed in the non-proliferative hiPSC-CMs, which were cultured without insulin or CHIR (Figures 2C and 2D). Additionally, we noted a significant increase in cell size upon withdrawal of CHIR from the media (Figures 2C and 2E). Taken together, these findings indicate that insulin and CHIR inhibit the expression of mature cardiomyocyte markers in hiPSC-CMs.

Sarcomere structure and contractile properties in response to insulin and GSK3 inhibition

The regulation of cardiac contractile function is dependent upon the structure and organization of the sarcomeres. In addition to the observed impact of insulin and CHIR on the expression of sarcomere-associated genes and protein profiles, we further conducted a previously established morphological analysis of sarcomere structure under the designed conditions (Homan et al., 2021). Our analysis of the order and dispersion parameters demonstrated that hiPSC-CMs cultured under the B27(INS$^-$) CHIR$^-$ condition displayed the most highly organized cTnT striations when compared to cells cultured in B27(INS$^+$) CHIR$^-$ (Figures 3A, 3B, and S3A). In contrast to hiPSC-CMs cultured in B27(INS$^-$) CHIR$^-$, the addition of CHIR (B27(INS$^-$) CHIR$^+$) led to a significant increase in dispersion (0.261 versus 0.653) (Figure 3B, Dispersion) and a decrease in the order parameter (0.785 versus 0.361) (Figure 3B, Order). This difference in dispersion in response to CHIR was amplified by the addition of insulin (B27(INS$^+$) CHIR$^+$) (0.346 versus 0.706) (Figure 3B, Dispersion). Moreover, average sarcomere length was 1.703 μm in the B27(INS$^+$) CHIR$^-$ condition, versus an increased sarcomere length with an average of 1.798 μm in hiPSC-CMs cultured in B27(INS$^-$) CHIR$^-$ (Figure 3B, Sarcomere length). In contrast, the addition of CHIR significantly reduced the average sarcomere length to 1.467 and 1.401 μm in hiPSC-CMs cultured in B27(INS$^-$) CHIR$^+$ and B27(INS$^+$) CHIR$^+$, respectively (Figure 3B, Sarcomere length). Previous studies have shown an association between sarcomere function and polyploidization (Pettinato et al., 2021; Yuan et al., 2022), and, in line with that, we observed a larger proportion of mononucleated hiPSC-CMs in the presence of CHIR, versus a larger proportion of multinucleated cells in the absence of CHIR, independent from insulin (Figures S3B–S3D).

Next, we evaluated the contraction, relaxation, and calcium kinetics of hiPSC-CMs (Dinani et al., 2023) with and without CHIR and/or insulin (Figure 3C). In all four conditions we found contractile cells with active calcium handling, indicating that these cells were functional cardiomyocytes. Contraction duration was not altered significantly, while relaxation was shortened in the absence of pathway stimuli (Figure 3D). Next, calcium tracings showed similar Tau decay for all four conditions, while calcium amplitude and upstroke velocity were shorter in the absence of CHIR; however, spontaneous beating frequencies also differed per condition (Figures 3E and S4). Taken together, these results highlight that mostly CHIR and to a lesser extent insulin signaling negatively impact sarcomere development. At the two-dimensional level all treatment groups were functional, but no important signs of mature contractility or calcium kinetics were observed in the absence of growth stimuli.

Metabolic function and substrate utilization in response to GSK3β inhibition and insulin

The metabolic compartmentalization of the embryonic heart displays an enhanced glycolytic program in the compacted myocardium, versus more fatty acid-oxidative metabolic utilization in the more differentiated trabeculae (Menendez-Montes et al., 2016). This metabolic

(D and E) Quantification of the MLC2v$^+$ intensity (D) and cell surface area; cTnT signal was thresholded and binarized, and holes were filled, whereafter the area per cell was measured (E) in 3 independent batches of hiPSC-CMs of the ● = SCVI-114 line. (B) Data are presented as mean ± SEM. Significance is determined by one-way ANOVA followed by Dunn's multiple comparisons test standardized to B27(INS$^-$) CHIR$^-$ condition. (D and E) Data are presented as medians and compared using the Kruskal-Wallis test. $n = 3$ independent experiments from 3 different cell lines (SCVI-273, SCVI-114, and SCVI-111), unless specified different. *$p < 0.05$, **$p < 0.01$, ***$p < 0.001$, and ****$p < 0.0001$; ns, not significant.

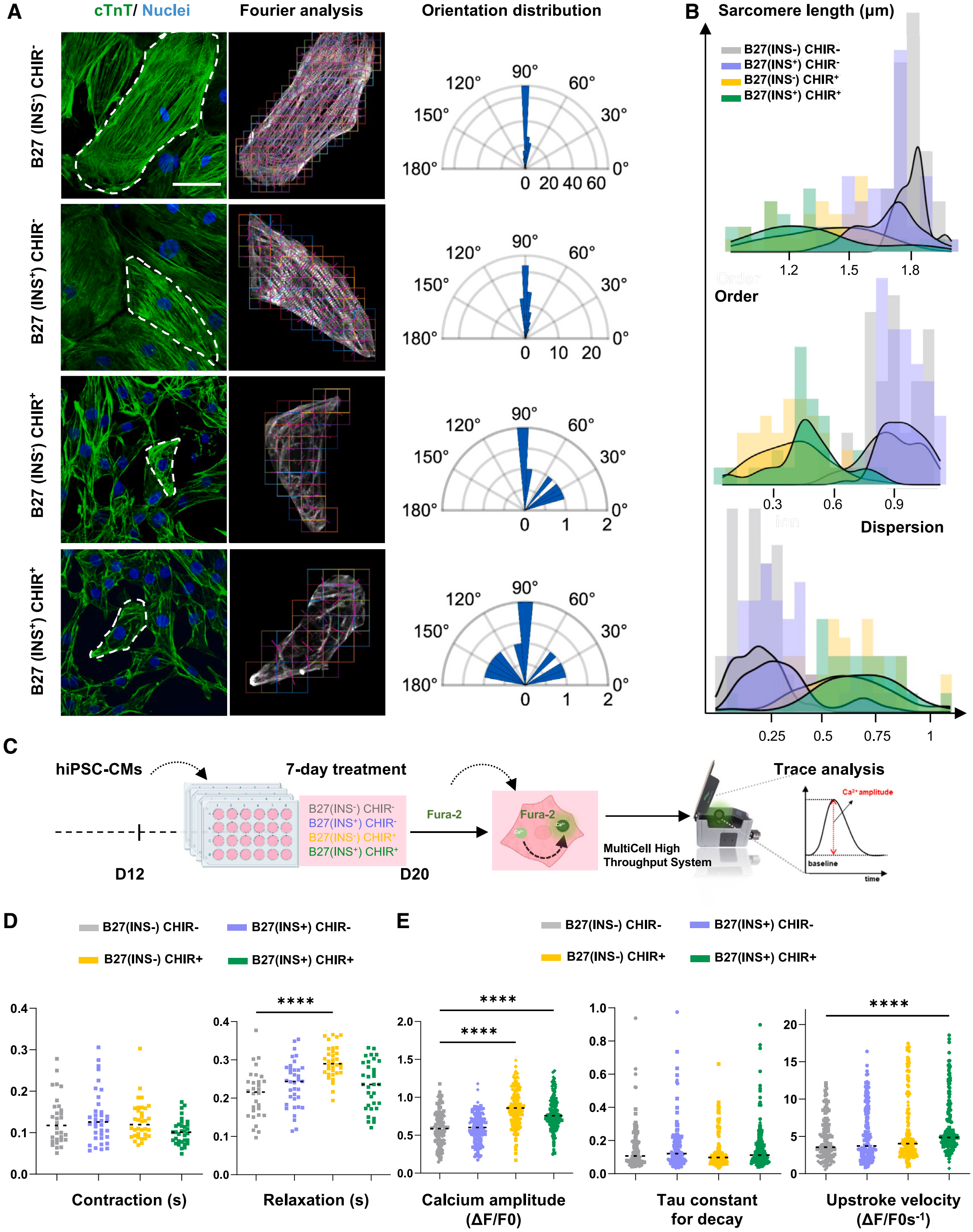

Figure 3. Structural and functional characterization of contractile hiPSC-CMs treated with insulin and/or CHIR99021

(A) Representative immunofluorescence images of cardiac Troponin T (cTnT) (green) staining and automated sarcomere orientation and distribution analysis using the MorphoScript tool. Nuclei visualized with DAPI (blue). Scale bar, 50 μm.

(B) Sarcomere length (upper), order (middle), and dispersion (lower) for indicated treatments presented as histograms. The y axis on each histogram indicates normalized density.

(C) Schematic of treatments followed by calcium transient and contractility analysis.

(D) Scatterplots of contractility analyses; contraction time (time to peak 70%) and relaxation time (time to baseline 70%) in ■ = SCVI-273, 3 biological experiments in 10–12 technical replicates per condition.

(legend continued on next page)

compartmentalization follows the pattern of highly proliferative cardiomyocytes in the compacted myocardium versus more differentiated trabeculae areas (Buikema et al., 2013, 2014; Jeter and Cameron, 1971; Luxán et al., 2013; Ye et al., 2015). To characterize mitochondrial content and function, we used day 12 immature hiPSC-CMs and treated them under the described conditions (Figure 4A). After the four different treatment regimens, we found the highest mitochondria content (Tom20 intensity/cell) in hiPSC-CMs cultured without insulin and CHIR, versus insulin alone and/or CHIR/insulin combinations (Figures 4B and 4C). Moreover, we mostly observed a perinuclear localization of mitochondria in cells cultured with CHIR, as an indicator of immaturity, versus combined perinuclear and cytoplasmic localization of mitochondria in cells cultured without CHIR (Figure 4B). In contrast, insulin played a facilitating role in hiPSC-CMs in terms of mitochondrial respiration, as evidenced by the enhancements observed in basal oxygen respiration, ATP-linked respiration, proton leak, and spare reserve capacity (Figures 4D–4H). Conversely, the inhibition of GSK3β led to further reductions in these parameters of mitochondrial respiration, irrespective of insulin presence (Figures 4D–4H). Additionally, substrate-blocking assays performed in different media compositions revealed that in hiPSC-CMs cultured without insulin, and supplemented with fatty acids, the oxygen consumption rate was restored to a level comparable to when insulin was present in the media (Figures S5A–S5D). This indicated a relative dependency on fatty acids for ATP production in cells cultured without insulin and CHIR. To this end we verified the uptake of fatty acids in cells treated with all four groups and indeed found that in the absence of insulin and CHIR, the hiPSC-CMs had the highest uptake of fatty acids, as an indicator of more maturity of these cells (Figures S6A–S6C). Interestingly, insulin was less essential than CHIR in this process, and the addition of fatty acids to the culture media induced a switch to mitochondrial oxidative phosphorylation and decreased glycolysis dependency and glutamine metabolism (Figures S5B–S5D). These results collectively indicate that insulin is not essential for the metabolic switch of hiPSC-CMs but is required for the oxidation of substrates such as glucose and glutamine. Altogether, those results indicate that insulin and CHIR stimulation led to decreased mitochondria content, lower energy production, and repression of fatty acids utilization in hiPSC-CMs.

RNA sequencing identifies FOXO as candidate mediating the interplay between proliferation and maturation of hiPSC-CMs

To enhance our understanding of the effects of insulin and CHIR on proliferation and maturation in immature hiPSC-CMs, we conducted high-throughput transcriptome sequencing (RNA sequencing [RNA-seq]) to evaluate the differences among the described culture conditions. The top 50 differentially expressed genes (DEGs) were highlighted to those linked to embryonic pathways in cardiac differentiation and development. Notably, these include sarcomere structure-related genes *MYL2* and *MYOM2*, as well as up- and downstream regulating genes of the insulin/PI3K-Akt signaling pathway, such as *IGFBP5*, *PIK3IP1*, and *FOXO4*; WNT signaling genes *WNT2* and *AXIN2*; and other genes involved in proliferation and differentiation such as *BMP10*, *MYBL2*, *FRAT1*, *HOPX*, and *DLK1* (Figure 5A). Principal-component analysis (PCA) revealed that CHIR contributed to the greatest variation (87.95%) among these DEGs, involving 5,336 variables. In contrast, insulin only contributed 6.04% (994 variables), and the combined influences of insulin and CHIR accounted for 7,003 variables (Figure 5B). Notably, our volcano plots displayed a gradient upregulation of the *WNT2* gene activated by CHIR, interestingly, with a sequential increase in significance following the addition of insulin, CHIR, and the combination of insulin and CHIR (Figure 5C and Table S3). This pattern indicates that insulin and CHIR exhibit effects on *WNT2* activation. Similarly, sarcomere-associated genes *MYOM2* and *MYL2* showed a gradual downregulation induced by insulin and/or CHIR (Figure 5C), reinforcing our observation of their effects on proliferation and sarcomere development for immature hiPSC-CMs (Figures 1 and 3). Moreover, this impact further extended to cellular gap junctions (*GJA5*) and also affected differentiation-associated genes (*MYOM2*, *DLK1*, *KCNA5*) (Figure 5C). Interestingly, the FOXO family member *FOXO4* also displayed a gradual upregulation in the absence of insulin and CHIR, such as that observed with *WNT2* activation (Figure 5C). To explore functional batch annotation and ontology enrichment of the DEGs, we conducted Kyoto Encyclopedia of Genes and Genomes (KEGG) pathway analysis. As expected, the activated WNT target genes in DEGs showed significant enrichment in cell-cycle-related pathways (Figures 5D and S2K). Moreover, our analysis suggested that this effect is likely due to the suppression of FOXO

(E) Scatterplots of calcium handling dynamics; calcium amplitude, upstroke velocity, and Tau decay. (D and E) Data are represented as mean ± SD. $n \geq 30$ cells for each condition were analyzed from $N = 3$ independent experiments from 3 different cell lines (■ = SCVI-273, ◆ = SCVI-202, and ● = SCVI-114) (Figure S4), unless specified otherwise. Comparisons are performed by Kruskal-Wallis test followed by Dunn's multiple comparisons test with B27(INS$^-$) CHIR$^-$ as reference condition. Not significant, $p > 0.05$, $^*p < 0.05$, $^{**}p < 0.01$, $^{****}p < 0.0001$.

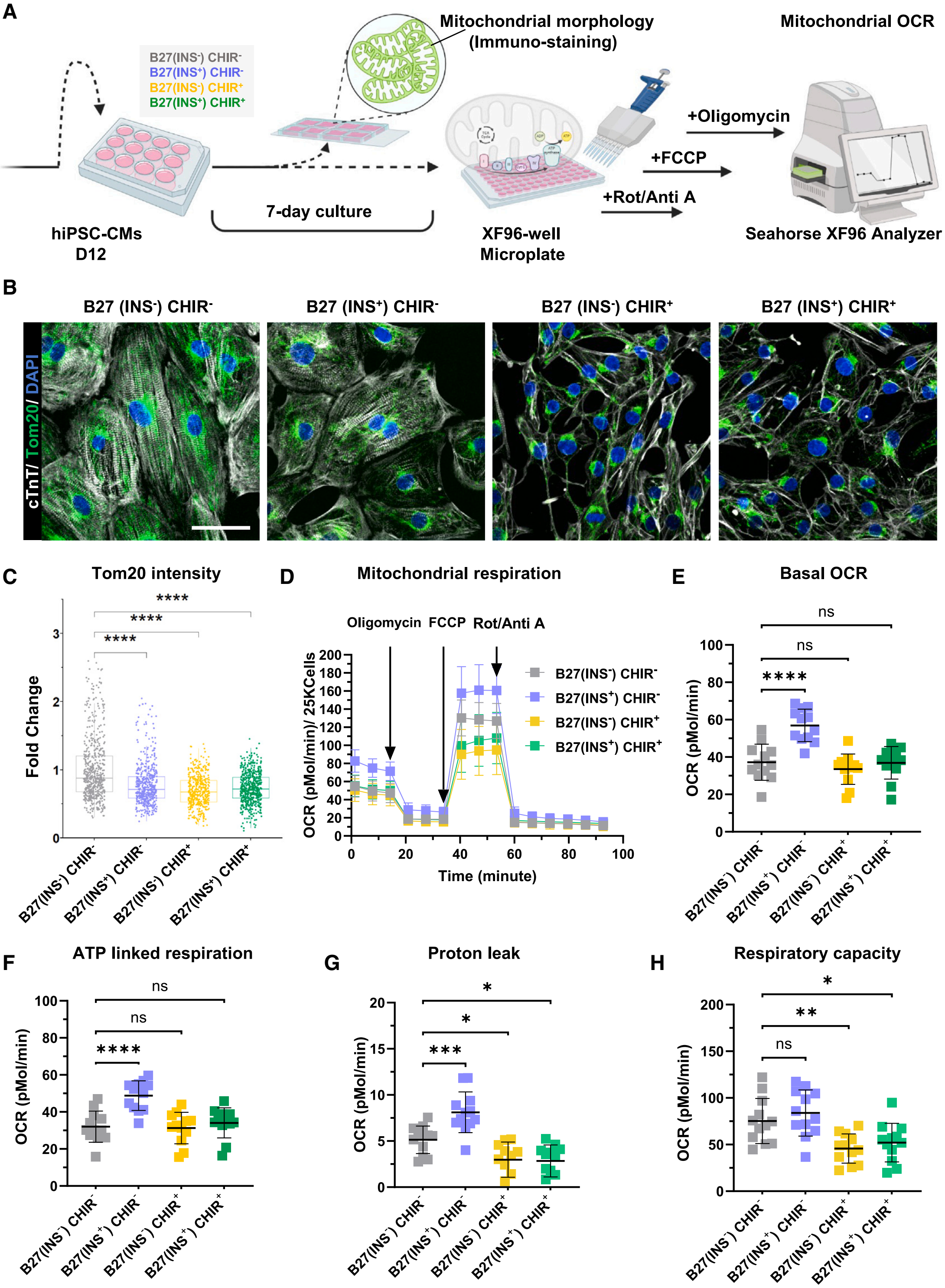

Figure 4. The effects of insulin and CHIR99021 on mitochondrial distribution and function

(A) Schematic of experimental setup for mitochondria assays.

(B) Representative immunofluorescence images of cTnT (white) and Tom20 (green). DAPI staining for nuclei (blue). Scale bar, 200 μm.

(C) Quantification of Tom20 immunofluorescence intensity in ±550 cells/condition, relative to B27(INS^{-}) CHIR^{-}.

(legend continued on next page)

via insulin (Figures 5D and 5E). In addition to DEGs and pathway enrichment, gene ontology (GO) analysis highlighted significant changes in molecular functions related to tubulin and actin binding (Figures S2G–S2I). Correspondingly, the effects of insulin and/or CHIR were specifically evident in the contractile machinery, involving Z-disc, sarcomere, myofibril, actinin binding, and I band, in addition to cell-cycle activity (Figure S2J). Taken together, these data support the observation that activation of cell-cycle genes is inversely associated with mature cardiomyocyte genes. Moreover, our analysis identifies FOXO as a potential downstream target of insulin signaling in hiPSC-CMs renewal.

Molecular inhibition of FOXO signaling rescues cell-cycle exit of immature hiPSC-CMs

Recently, the molecular inhibition of FOXO with AS1842586 was found to increase hiPSC-CMs proliferation (Schade et al., 2022). We further conducted the investigation into the role of insulin/FOXO signaling in CHIR-stimulated proliferation of hiPSC-CMs (Figure 6A). When hiPSC-CMs were cultured under FOXO inhibition with AS1842586, we observed a 6-fold increase in the proliferation rate of immature hiPSC-CMs (D12) after 4 days. This led to an approximate 2.4-fold increase in the number of cTnT-positive cells among hiPSC-CMs, even in the absence of insulin in the culture. Remarkably, this outcome was comparable to the effect observed with CHIR alone (without insulin), which stimulated an increase in both cTnT-positive cell number and the proliferation rate ($Ki67^+/cTnT^+$) of immature hiPSC-CMs (D12) (Figures 6B–6D). This suggests that the activation of FOXO in the culture of immature hiPSC-CMs inhibited proliferation, as predicted by RNA-seq analysis (Figures 5C and 5D). However, this proliferative effect of FOXO inhibition did not seem to be induced through direct upregulation of Wnt target genes (Figure 6E). Significantly, when insulin was reintroduced into the culture in the presence of AS1842586, there was a 6.5-fold increase in the proliferation rate and a 4-fold increase in cTnT-positive cell number of immature hiPSC-CMs compared to the culture without insulin or AS1842586 stimulation (Figures 6C and 6D). Notably, the increase in cTnT-positive cell number induced by insulin and FOXO inhibition was comparable to the CHIR-stimulated proliferation of hiPSC-CMs (Figure 6C). Importantly, to rule out the possibility that the increase in cTnT-positive cell numbers and the proliferation rate (Ki67+/cTnT+) we observed is rather due to a protective effect from apoptosis, we show that all of the conditions maintain equal cellular viability (~90%) (Figure S7). Collectively, the inhibition of FOXO facilitated proliferation of hiPSC-CMs, presumably in part by shortcutting the insulin-Akt route.

Stage-specific effects of insulin and Wnt signaling pathways on early- and late-stage hiPSC-CMs

In the developing heart, the proliferative capacity of fetal cardiomyocytes rapidly diminishes shortly after birth (Li et al., 1996). This phenomenon has also been observed in hiPSC-CMs following maturation. To this end, we prolonged the culture of hiPSC-CMs in the widely used RPMI 1640 basal medium supplemented with B27, including insulin, to 40 days following the initial differentiation at day 12 (Figure 7A). To evaluate potential stage-specific effects of embryonic proliferation signals, we introduced insulin and/or CHIR in the "late-stage" hiPSC-CMs at day 40. We observed up to a 4-fold increase in Ki67+/cTnT+ cells upon stimulation with CHIR and insulin when compared to no insulin/CHIR. The stimulation of "late-stage" hiPSC-CMs with insulin and CHIR did not result in more cTnT+ cell numbers, indicating that only a small subset of "late-stage" hiPSC-CMs was still responsive to the signaling molecules (Figures 7B–7D). In this context, we examined the activity of β-catenin/TCF, a center effector of the canonical Wnt/β-catenin signaling pathway that promotes cell-cycle progression (Nusse and Clevers, 2017), via TOPFlash expression in hiPSC-CMs. Our data revealed a significant decreased TCF activity upon CHIR stimulation in "late-stage" hiPSC-CMs when compared to "early-stage" hiPSC-CMs (Figures 7E and 7F). Collectively, our findings demonstrate that insulin and CHIR exhibit stage-specific effect on proliferation, associated with lower nuclear TCF activation in "late-stage" versus "early-stage" hiPSC-CMs.

(D) Overview of changes in mitochondrial oxygen consumption rate in response to oligomycin, FCCP, (carbonyl cyanide 4-(trifluoromethoxy)phenylhydrazone), and Rot/Anti A (rotenone and antimycin A). Per well 25K cells were plated.
(E) Basal oxygen consumption rate (OCR) calculated by subtracting the non-mitochondrial respiration from the basal OCR.
(F) ATP-linked respiration calculated based on the OCR after oligomycin injection.
(G) Proton leak obtained as the remaining OCR, which is not coupled to ATP-linked respiration.
(H) Respiratory capacity of mitochondria measured by deduction of basal OCR from the maximum OCR in response to FCCP injection. (C) Data are presented as medians and compared using the Kruskal-Wallis test, $N \geq 5$ independent cultures from 3 batches of SCVI-273 cell line. (D–H) Data are presented as mean ± SD, and statistical significance tested with one-way ANOVA followed by Tukey's multiple comparison test. $n \geq 11$ independent cultures from 3 batches of SCVI-273 cell line. *$p < 0.05$, **$p < 0.01$, ***$p < 0.001$, ****$p < 0.0001$; ns, not significant.

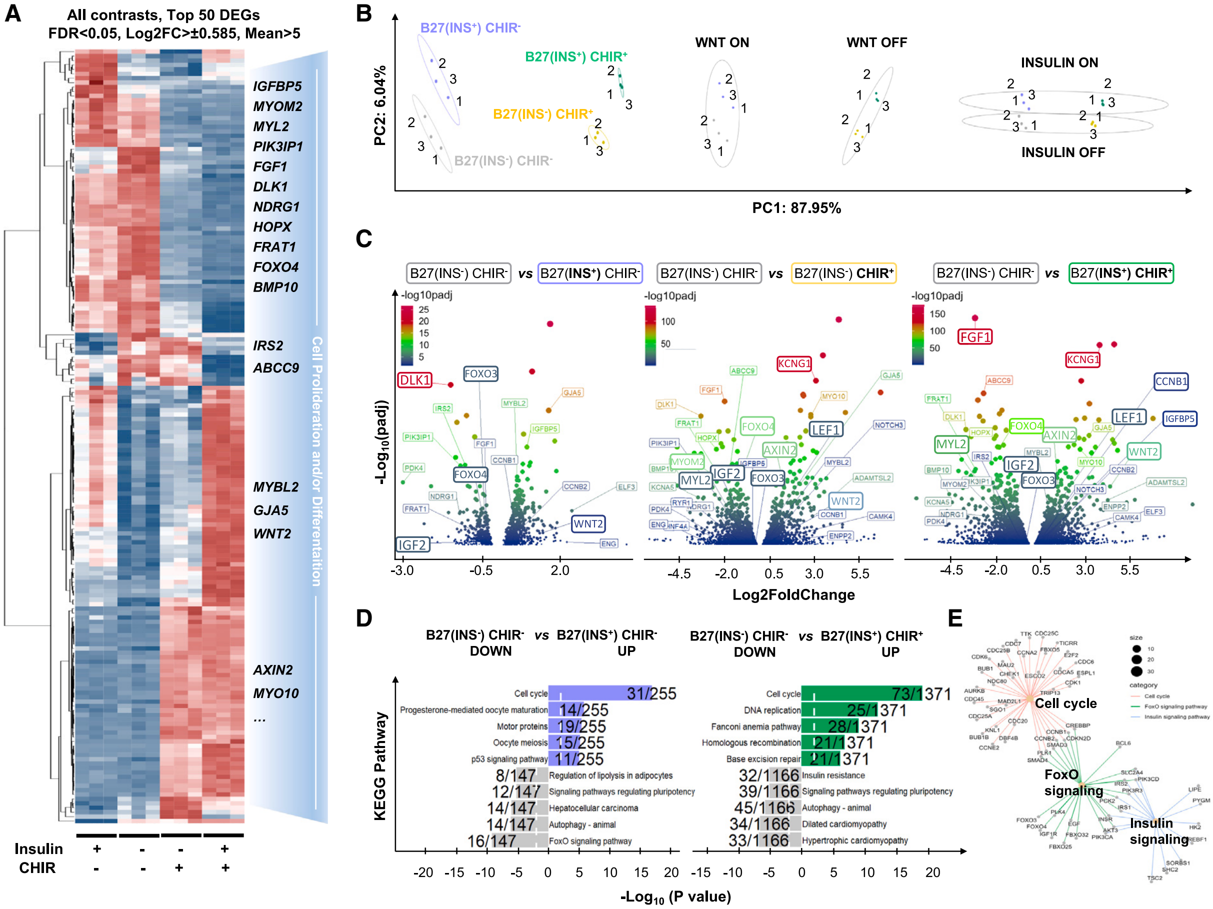

Figure 5. RNA sequencing analysis of hiPSC-CMs treated with insulin and/or CHIR99021

(A) Heatmap of top 50 differentially expressed genes (DEG) filtered based on base mean ≥ 5, log2fc >= 0.585 or =< -0.585, FDR < 0.05; pink to red highlights increased gene expression, and the light to dark blue denotes decreased gene expression.

(B) PCA plots distinguishing between CHIR and/or insulin treatment. $N = 3$ independent cultures from SCVI-273 cell line.

(C) Volcano plots of highlight DEGs of the following conditions: B27(INS$^+$) CHIR$^-$, B27(INS$^-$) CHIR$^+$, and B27(INS$^+$) CHIR$^+$ each compared versus B27(INS$^-$) CHIR$^-$ (Table S3).

(D) KEGG enrichment analysis between conditions.

(E) Molecular network of insulin signaling pathway and FOXO signaling pathway for Wnt signaling-activated cell cycle.

DISCUSSION

Here we report dual effects of insulin and CHIR99021 on proliferation of immature hiPSC-CMs and blocking of maturation, while the removal of these pathway activators results in rapid cell-cycle exit (Figure 1) and further differentiation (Figures 2B and 3). While CHIR inhibits GSK3β and activates canonical Wnt signaling via TCF-mediated target gene transcription, we also identify a novel interplay between insulin-Akt and FOXO transcription as regulator between proliferation and maturation of immature hiPSC-CMs (Figures 5 and 6).

In the context of B27, commonly used as a supplement to the basal RPMI 1640 medium for hiPSC-CM culture, insulin, a component in B27, acts as a stimulant of the PI3K-AKT pathway, leading to the phosphorylation of GSK3β (Abel, 2021; Buikema et al., 2020). Our work provides insights into the positive effects of insulin on proliferation and energy production in hiPSC-CMs (Figures 1 and 4). On the contrary, insulin has a negative impact on the differentiation and organization of the sarcomeres (Figures 2 and 3). Therefore, the concentration of insulin in the media would be an important consideration for the production of more mature cardiomyocytes.

Our RNA-seq analysis revealed that insulin upregulated cell-cycle signaling pathways, while the removal of insulin led to a significant upregulation of FOXO signaling (Figure 5). Undoubtedly, the insulin-mediated PI3K-Akt

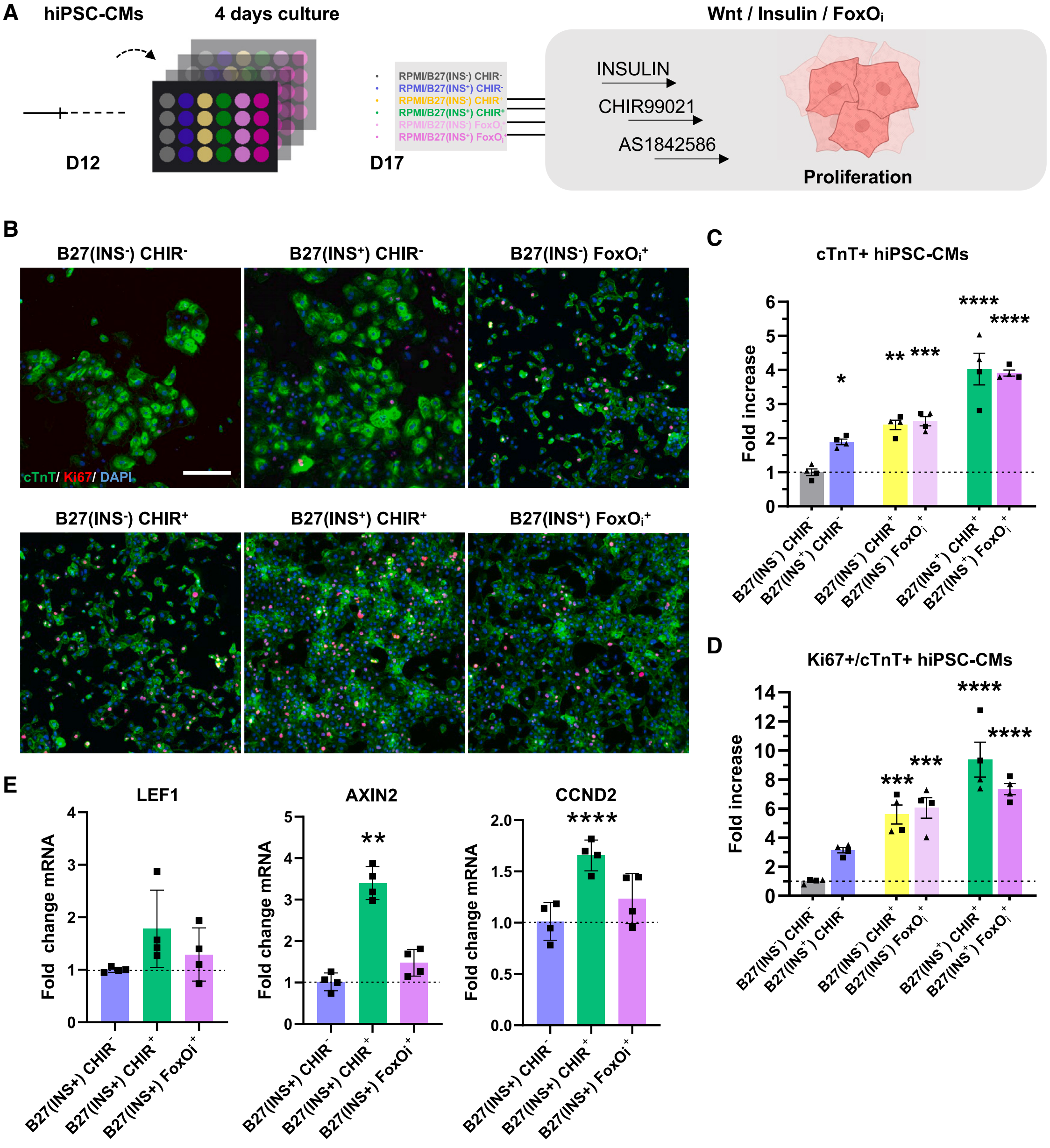

Figure 6. WNT activation (CHIR99021)/FOXO inhibition (AS1842856) promotes early immature hiPSC-CM proliferation

(A) Schematic of timeline for investigating the effects of Wnt/Insulin/FOXO inhibition (FOXOi) on the proliferation of hiPSC-CMs.

(B) Representative immunofluorescence images of hiPSC-CMs subjected to cTnT antibody (green), Ki67 antibody (red), and DAPI for indicated conditions. Scale bar, 200 μm.

(C and D) Quantification of cTnT-positive hiPSC-CMs (C) and the fraction of Ki67-and cTnT-positive hiPSC-CMs (D) by fold increase under the indicated culture conditions. Data are presented as mean ± SEM. $n = 4$ independent cultures from 2 different cell lines (■ = SCVI-273, ▲ = SCVI-111).

(E) Fold mRNA changes of distinct Wnt target genes after 24 h treatment with the indicated culture conditions. Data are presented as mean ± SD. $N = 4$ biological replicates of each 3 technical replicates were performed using the SCVI-273 line. Data normalized to GAPDH. Statistical significance tested using one-way ANOVA followed by Dunn's test for multiple comparisons relative to control B27(INS^{-}) CHIR^{-} (C and D) and B27(INS+) CHIR- (E), *$p < 0.05$, **$p < 0.01$, ***$p < 0.001$, and ****$p < 0.0001$.

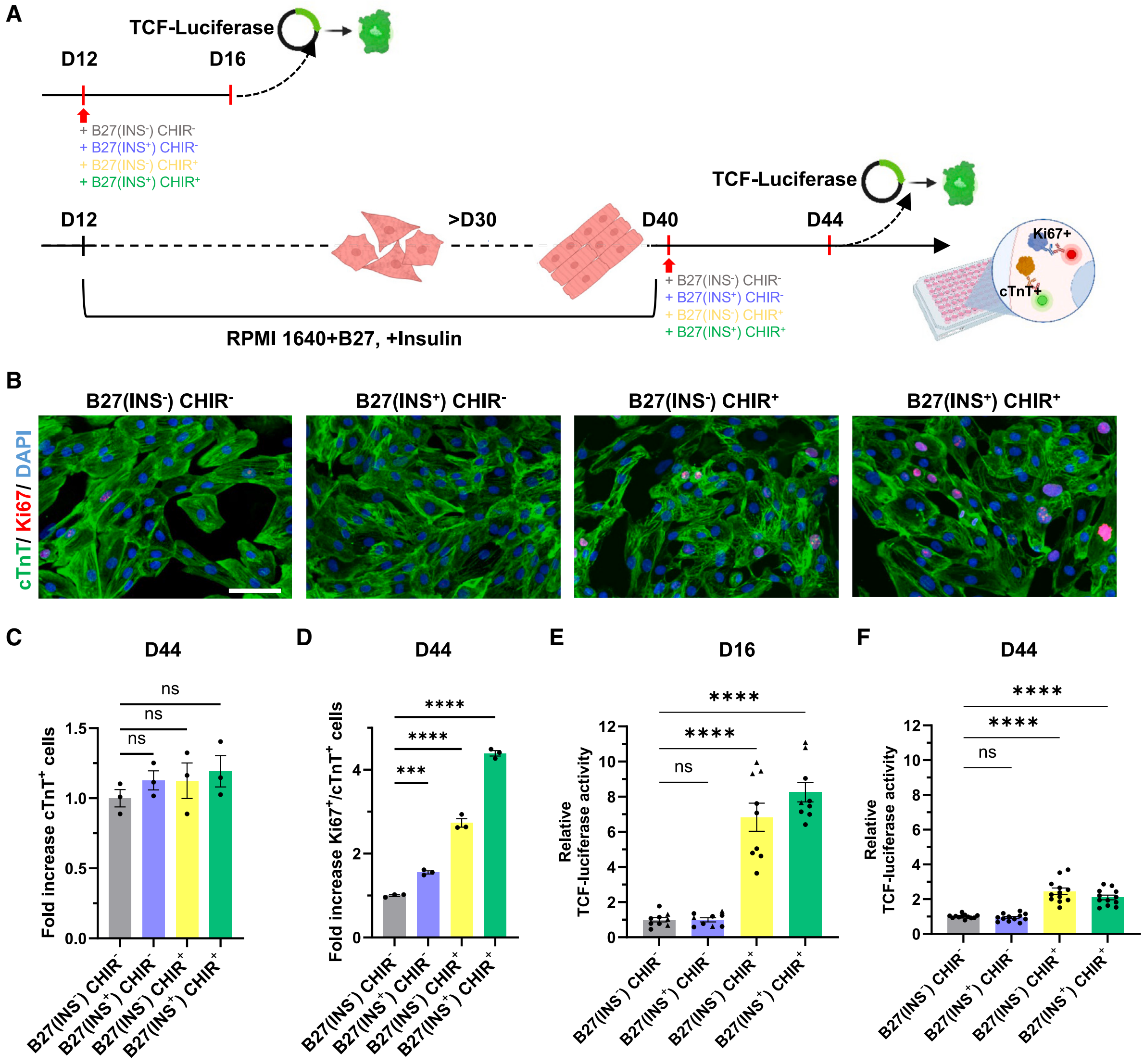

Figure 7. Impaired effects of insulin and Wnt/β-catenin signaling on late-stage hiPSC-CM proliferation

(A) Schematic of early-stage and late-stage hiPSC-CMs proliferation assays.

(B) Representative immunofluorescence images of hiPSC-CMs subjected to cTnT antibody (green), Ki67 antibody (red), and DAPI under the designed culture conditions. Scale bar, 50 μm.

(C and D) Quantification of cTnT+ hiPSC-CMs (C) and fraction of Ki67+/cTnT+ hiPSC-CMs (D) in late-stage (D44) iPSC-CMs by fold increase under the indicated culture conditions.

(E and F) Fold increase in luciferase activity after transfection of luciferase reporter (TOPFlash) in early-stage (D16) hiPSC-CMs (E) and late-stage (D44) hiPSC-CMs (F). Data presented as mean ± SEM. $n \geq 3$ independent cultures from 2 different hiPSC lines (● = SCVI-114, ▲ = SCVI-111). Statistical significance was assessed by one-way ANOVA followed by Dunn's test for multiple comparisons relative to B27(INS^{-}) CHIR^{-}. Not significant (ns), $p > 0.05$, $***p < 0.001$, and $****p < 0.0001$.

signaling cascade serves as a pivotal regulator of FOXO (Litwiniuk et al., 2016). This signaling pathway plays a crucial role in regulating proliferation in both cultured primary embryonic cardiomyocytes (Evans-Anderson et al., 2008) and hiPSC-CMs (Schade et al., 2022). In our studies we found that addition of the FOXO inhibitor AS1842856 to media depleted from insulin and CHIR resulted in proliferation of hiPSC-CMs. This suggests that without insulin in the media, in part FOXO acts as a negative regulator of hiPSC-CM expansion, and subsequent inhibition of FOXO leads to cell division. Moreover, our experiments combining insulin with a FOXO inhibitor demonstrated a more powerful proliferative response, also indicating indirect effects of

AS1842856 (Figure 6). These findings suggest a promising direction for further investigation.

Mills et al.'s investigation into human pluripotent stem cell-derived cardiac organoids revealed that the absence of insulin in the culture medium, when exposed to palmitate, induces cell-cycle arrest and instigates a metabolic shift (Mills et al., 2017). Furthermore, Garay et al.'s work underscored the significance of concurrent inhibition of both the PI3K-AKT pathway and mitogen-activated protein kinase pathway that promotes the maturation of hiPSC-CMs across multiple facets, encompassing transcriptional profiles, sarcomere development, metabolism, and electrophysiology (Garay et al., 2022). In our studies we found that CHIR has significant negative influences on the localization and function of mitochondria. Moreover, insulin had a positive effect on overall metabolism of hiPSC-CMs. Interestingly, in the absence of CHIR and insulin, we found an increase in mitochondria and a preference for fatty acid uptake (Figures 4, S5, and S6).

Notably, our assays in "early-stage" and "late-stage" cardiomyocytes indicated a stage-specific effect of CHIR and insulin on the robust proliferation of hiPSC-CMs. In part, this could be the result of impaired TCF activation by β-catenin such as investigated in the TOPFlash luciferase reporter assay (Figure 7). Alternatively, β-catenin may already have different transcriptional effects in these "late-stage" hiPSC-CMs when compared to the "early-stage," such as has been studied by Quaife-Ryan et al. before (Quaife-Ryan et al., 2020). Moreover, the treatment of hiPSC-CMs with CHIR results in rapid disorganization of sarcomeres either via canonical Wnt signaling or via direct effects on GSK3, resulting in proliferation. Additional evidence suggests that sarcomeres negatively regulated cell replication and promoted polyploidization by activating p53-dependent DNA damage (Pettinato et al., 2021). In line with that evidence, our study observed a most profound effect of insulin and CHIR99021 on producing mononuclear hiPSC-CMs (Figure S3).

Moreover, we found that CHIR99021-mediated GSK3β inhibition disrupts sarcomere structure, which is independent of insulin (Figures 3A and 3B). This implies that inducing sarcomere disassembly through CHIR could be a potential strategy to promote renewal of cardiomyocytes. Although no dramatic effects in calcium handling and contractility were observed in the presence or absence of CHIR and insulin, this could be explained by a number of factors such as increased cell growth, varying spontaneous beating frequencies (Figure S4E), and the two-dimensional culture set-up. For future studies it would be of interest to test the effect of Wnt and insulin in three-dimensional cardiac tissues.

In summary, we provide novel insights into the cues to maintain cardiomyocyte proliferation versus maturation via an interplay between the insulin/FOXO and Wnt/GSK3β/β-catenin/TCF signaling routes. The understanding of embryonic signaling routes would be of importance for future regenerative strategies in heart failure.

EXPERIMENTAL PROCEDURES

HiPSCs culture and differentiation to hiPSC-CMs

Four independent hiPSC lines from the Stanford Cardiovasculair Institute (SCVI) biobank (SCVI-273, SCVI-114, SCVI-202, and SCVI-111) were cultured on Matrigel-coated general cell culture plates and maintained in E8 basal medium supplemented with E8 supplement (1×) (Life Technologies). Medium changes were performed daily, and passaging and initiation of differentiation were performed at 70%–80% confluency. For differentiation into hiPSC-CMs, we used the Wnt-modulation protocol with chemical GSK3 (CHIR99021) and Wnt (C59) inhibitors as previously described (Lian et al., 2013). For the TC1133-ACTN2-Citrine-FUCCI hiPSC line (for a more detailed explanation and characterization of the cell line, see Kim et al., 2024), a different differentiation protocol was used; at day 0 we subjected the cells to base medium (RPMI 1640 + GlutaMAX, 2% B27(INS-), 200 μmol/L L-ascorbic acid, 1 mmol/L Na-pyruvate, 100 U/mL penicillin, 100 μg/mL streptomycin) supplemented with 9 ng/mL Activin A (Bio-Techne), 1 μmol/L CHIR99021 (Merck Chemicals GmbH), 5 ng/mL BMP4 (Bio-Techne), and 5 ng/mL FGF (PreproTech) for 3 days. Afterward, the base medium was supplemented with 5 μmol/L IWP4 (ReproCELL) for cardiomyocyte differentiation for 9 days. All hiPSC lines were between passage number 20 and 35.

Immunofluorescence staining and confocal microscopy imaging

See supplemental information for a detailed description and key resource table for details of antibodies used.

RT-qPCR

See supplemental information and Table S1 for a list of primers used.

Contractility measurements and calcium transient analysis

See supplemental information.

Seahorse XF Mito stress assay

See supplemental information.

Seahorse XF Mito Fuel Flex assay

See supplemental information.

Fatty acid uptake assay

See supplemental information.

Bulk RNA-seq analysis

See supplemental information and GEO submission GSE278598.

Luciferase-based LEF/TCF activity analysis

See supplemental information.

Quantification and statistical analysis

Statistical analysis and visualization were conducted using GraphPad Prism version 9 software and/or R v4.0.2. Data were tested for normality using Pearson D'Agostino's K-squared test and analysis of QQ plots. For normally distributed data, student's t test or ordinary one-way ANOVA followed by Tukey's *post hoc* multiple comparisons (comparisons between all group means) or Dunn's multiple comparisons test (comparisons to control condition) was performed. Non-parametric data with multiple groups were analyzed using Kruskal-Wallis test followed by Dunn's multiple comparisons test. The levels of significance were represented by the following p values: $p > 0.05$ (ns, not significant), $^{*}p \leq 0.05$, $^{**}p \leq 0.01$, $^{***}p \leq 0.001$, and $^{****}p \leq 0.0001$.

hiPSC lines

Research on hiPSC was approved by the medical ethical committee at Amsterdam UMC, the Netherlands. A detailed list of hiPSC lines used for the experiments is provided in the STAR Methods in supplemental information.

RESOURCE AVAILABILITY

Lead contact

Request for further information or more detailed protocols should be directed to and will be fulfilled by the corresponding author, Jan W. Buikema (j.w.buikema@amsterdamumc.nl).

Materials availability

This study did not generate new unique reagents.

Data and code availability

The RNA-seq data were reposited under GEO accession number GSE278598.

ACKNOWLEDGMENTS

We thank Joseph C. Wu from Stanford University for providing lines SCVI-111, SCVI-114, SCVI-202, and SCVI-273. We thank Amsterdam UMC (location VUmc; O2 building) Microscopy and Cytometry Core Facility (MCCF) for the technological support. We thank Babu Kurakula from Amsterdam University Medical Center for sharing Renilla luciferase reporter plasmid. This research was supported by grants from the Chinese Scholarship Council (CSC no. 201706170068) (Q.Y.), the Dutch Heart Foundation Dekker Senior Clinical Scientist grant (J.W.B.) and the Netherlands Organization for Scientific Research (NWO VICI, grant 91818602)(JvV).

AUTHOR CONTRIBUTIONS

Experimental design, data analysis, and manuscript writing, Q.Y., D.V., and J.W.B.; experimental procedures and Figures 1 and S1, Q.Y., R.K., E.S., and D.V.; Figures 2 and S1, Q.Y.; Figures 3 and S2A–S2F, S.A.S., C.D.M., and Q.Y.; Figures 4 and S3, Q.Y. and R.D.; Figure S4, R.D., D.V., and Q.Y.; Figures 5 and S6, Q.Y. and D.V.; Figure 6, Q.Y., L.J.M.K., and D.V.; Figure 7, Q.Y. Manuscript reading and editing was done by J.v.d.V., D.W.D.K., L.C.Z., D.v.d.V., J.H., and R.A.B. All authors have read and agreed to the published version of the manuscript.

DECLARATION OF INTERESTS

The authors declare no competing interests.

SUPPLEMENTAL INFORMATION

Supplemental information can be found online at https://doi.org/10.1016/j.stemcr.2024.11.001.

Received: April 21, 2024
Revised: November 4, 2024
Accepted: November 4, 2024
Published: December 5, 2024

REFERENCES

Abel, E.D. (2021). Insulin signaling in the heart. Am. J. Physiol. Endocrinol. Metab. *321*, E130–E145. https://doi.org/10.1152/ajpendo.00158.2021.

Bersell, K., Arab, S., Haring, B., and Kühn, B. (2009). Neuregulin1/ErbB4 signaling induces cardiomyocyte proliferation and repair of heart injury. Cell *138*. 257–70. https://doi.org/10.1016/j.cell.2009.04.060.

Buikema, J.W., Lee, S., Goodyer, W.R., Maas, R.G., Chirikian, O., Li, G., Miao, Y., Paige, S.L., Lee, D., Wu, H., et al. (2020). Wnt Activation and Reduced Cell-Cell Contact Synergistically Induce Massive Expansion of Functional Human iPSC-Derived Cardiomyocytes. Cell Stem Cell *27*, 50–63.e5. https://doi.org/10.1016/j.stem.2020.06.001.

Buikema, J.W., Mady, A.S., Mittal, N.V., Atmanli, A., Caron, L., Doevendans, P.A., Sluijter, J.P.G., and Domian, I.J. (2013). Wnt/β-catenin signaling directs the regional expansion of first and second heart field-derived ventricular cardiomyocytes. Development *140*, 4165–4176. https://doi.org/10.1242/dev.099325.

Buikema, J., Zwetsloot, P.P., Doevendans, P., Domian, I., and Sluijter, J. (2014). Wnt/β-Catenin Signaling during Cardiac Development and Repair. J. Cardiovasc. Dev. Dis. *1*, 98–110. https://doi.org/10.3390/jcdd1010098.

Collesi, C., Zentilin, L., Sinagra, G., and Giacca, M. (2008). Notch1 signaling stimulates proliferation of immature cardiomyocytes. J. Cell Biol. *183*, 117–128. https://doi.org/10.1083/jcb.200806091.

Decourtye, L., Clemessy, M., Mire, E., Ledent, T., Périn, L., Robinson, I.C., Le Bouc, Y., and Kappeler, L. (2018). Impact of insulin on primary arcuate neurons culture is dependent on early-postnatal nutritional status and neuronal subpopulation. PLoS ONE *13*, e0193196. https://doi.org/10.1371/journal.pone.0193196.

D'Uva, G., Aharonov, A., Lauriola, M., Kain, D., Yahalom-Ronen, Y., Carvalho, S., Weisinger, K., Bassat, E., Rajchman, D., Yifa, O., et al. (2015). ERBB2 triggers mammalian heart regeneration by promoting cardiomyocyte dedifferentiation and proliferation. Nat. Cell Biol. *17*, 627–638. https://doi.org/10.1038/ncb3149.

Dinani, R., Manders, E., Helmes, M., Wang, L., Knollmann, B., Kuster, D.W.D., and van der Velden, J. (2023). Real-Time Measurements of Calcium and Contractility Parameters in Human Induced

Pluripotent Stem Cell-Derived Cardiomyocytes. J. Vis. Exp. *195*. https://doi.org/10.3791/65326.

Engels, M.C., Rajarajan, K., Feistritzer, R., Sharma, A., Nielsen, U.B., Schalij, M.J., de Vries, A.A.F., Pijnappels, D.A., and Wu, S.M. (2014). Insulin-like growth factor promotes cardiac lineage induction in vitro by selective expansion of early mesoderm. Stem Cell. *32*, 1493–1502. https://doi.org/10.1002/stem.1660.

Evans-Anderson, H.J., Alfieri, C.M., and Yutzey, K.E. (2008). Regulation of cardiomyocyte proliferation and myocardial growth during development by FOXO transcription factors. Circ. Res. *102*, 686–694. https://doi.org/10.1161/Circresaha.107.163428.

Fetterman, K.A., Blancard, M., Lyra-Leite, D.M., Vanoye, C.G., Fonoudi, H., Jouni, M., DeKeyser, J.-M.L, Lenny, B., Sapkota, Y., George, A.L., Jr., et al. (2024). Independent compartmentalization of functional, metabolic, and transcriptional maturation of hiPSC-derived cardiomyocytes. Cell Rep *43*, 114160. https://doi.org/10.1016/j.celrep.2024.114160.

Galdos, F.X., Guo, Y., Paige, S.L., VanDusen, N.J., Wu, S.M., and Pu, W.T. (2017). Cardiac Regeneration: Lessons From Development. Circ. Res. *120*, 941–959. https://doi.org/10.1161/CIRCRESAHA.116.309040.

Garay, B.I., Givens, S., Abreu, P., Liu, M., Yücel, D., Baik, J., Stanis, N., Rothermel, T.M., Magli, A., Abrahante, J.E., et al. (2022). Dual inhibition of MAPK and PI3K/AKT pathways enhances maturation of human iPSC-derived cardiomyocytes. Stem Cell Rep. *17*, 2005–2022. https://doi.org/10.1016/j.stemcr.2022.07.003.

Gonzales, K.A.U., Liang, H., Lim, Y.S., Chan, Y.S., Yeo, J.C., Tan, C.P., Gao, B., Le, B., Tan, Z.Y., Low, K.Y., et al. (2015). Deterministic Restriction on Pluripotent State Dissolution by Cell-Cycle Pathways. Cell *162*, 564–579. https://doi.org/10.1016/j.cell.2015.07.001.

Grego-Bessa, J., Gómez-Apiñaniz, P., Prados, B., Gómez, M.J., MacGrogan, D., and de la Pompa, J.L. (2023). Nrg1 Regulates Cardiomyocyte Migration and Cell Cycle in Ventricular Development. Circ. Res. *133*, 927–943. https://doi.org/10.1161/Circresaha.123.323321.

Grego-Bessa, J., Luna-Zurita, L., del Monte, G., Bolós, V., Melgar, P., Arandilla, A., Garratt, A.N., Zang, H., Mukouyama, Y.S., Chen, H., et al. (2007). Notch signaling is essential for ventricular chamber development. Dev. Cell *12*, 415–429. https://doi.org/10.1016/j.devcel.2006.12.011.

Heallen, T., Zhang, M., Wang, J., Bonilla-Claudio, M., Klysik, E., Johnson, R.L., and Martin, J.F. (2011). Hippo pathway inhibits Wnt signaling to restrain cardiomyocyte proliferation and heart size. Science *332*, 458–461. https://doi.org/10.1126/science.1199010.

Homan, T., Delanoë-Ayari, H., Meli, A.C., Cazorla, O., Gergely, C., Mejat, A., Chevalier, P., and Moreau, A. (2021). MorphoScript: a dedicated analysis to assess the morphology and contractile structures of cardiomyocytes derived from stem cells. Bioinformatics *37*, 4209–4215. https://doi.org/10.1093/bioinformatics/btab400.

Jeter, J.R., and Cameron, I.L. (1971). Cell Proliferation Patterns during Cytodifferentiation in Embryonic Chick Tissues - Liver, Heart and Erythrocytes. J. Embryol. Exp. Morphol. *25*, 405–422.

Kim, R., Nagel, S.H., Liaw, N.Y., Zimmermann, W.-H., Zelarayán, L.C., and Schoger, E. (2024). Human induced pluripotent stem cells for live cell cycle monitoring and endogenous gene activation. Stem Cell Res. *80*, 103531. https://doi.org/10.1016/j.scr.2024.103531.

Klaus, A., Saga, Y., Taketo, M.M., Tzahor, E., and Birchmeier, W. (2007). Distinct roles of Wnt/beta-catenin and Bmp signaling during early cardiogenesis. Proc. Natl. Acad. Sci. USA *104*, 18531–18536. https://doi.org/10.1073/pnas.0703113104.

Lazar, E., Sadek, H.A., and Bergmann, O. (2017). Cardiomyocyte renewal in the human heart: insights from the fall-out. Eur. Heart J. *38*, 2333–2342. https://doi.org/10.1093/eurheartj/ehx343.

Li, F., Wang, X., Capasso, J.M., and Gerdes, A.M. (1996). Rapid transition of cardiac myocytes from hyperplasia to hypertrophy during postnatal development. J. Mol. Cell. Cardiol. *28*, 1737–1746. https://doi.org/10.1006/jmcc.1996.0163.

Lian, X., Hsiao, C., Wilson, G., Zhu, K., Hazeltine, L.B., Azarin, S.M., Raval, K.K., Zhang, J., Kamp, T.J., and Palecek, S.P. (2012). Robust cardiomyocyte differentiation from human pluripotent stem cells via temporal modulation of canonical Wnt signaling. Proc. Natl. Acad. Sci. USA *109*, E1848–E1857. https://doi.org/10.1073/pnas.1200250109.

Lian, X., Zhang, J., Azarin, S.M., Zhu, K., Hazeltine, L.B., Bao, X., Hsiao, C., Kamp, T.J., and Palecek, S.P. (2013). Directed cardiomyocyte differentiation from human pluripotent stem cells by modulating Wnt/β-catenin signaling under fully defined conditions. Nat. Protoc. *8*, 162–175. https://doi.org/10.1038/nprot.2012.150.

Lin, Z., Zhou, P., von Gise, A., Gu, F., Ma, Q., Chen, J., Guo, H., van Gorp, P.R.R., Wang, D.Z., and Pu, W.T. (2015). Pi3kcb links Hippo-YAP and PI3K-AKT signaling pathways to promote cardiomyocyte proliferation and survival. Circ. Res. *116*, 35–45. https://doi.org/10.1161/CIRCRESAHA.115.304457.

Litwiniuk, A., Pijet, B., Pijet-Kucicka, M., Gajewska, M., Pająk, B., and Orzechowski, A. (2016). FOXO1 and GSK-3β Are Main Targets of Insulin-Mediated Myogenesis in C2C12 Muscle Cells. PLoS ONE *11*, e0146726. https://doi.org/10.1371/journal.pone.0146726.

Liu, L., Michowski, W., Kolodziejczyk, A., and Sicinski, P. (2019). The cell cycle in stem cell proliferation, pluripotency and differentiation. Nat. Cell Biol. *21*, 1060–1067. https://doi.org/10.1038/s41556-019-0384-4.

Lopaschuk, G.D., Spafford, M.A., and Marsh, D.R. (1991). Glycolysis Is Predominant Source of Myocardial Atp Production Immediately after Birth. Am. J. Physiol. *261*, H1698–H1705. https://doi.org/10.1152/ajpheart.1991.261.6.H1698.

Luxán, G., Casanova, J.C., Martínez-Poveda, B., Prados, B., D'Amato, G., MacGrogan, D., Gonzalez-Rajal, A., Dobarro, D., Torroja, C., Martinez, F., et al. (2013). Mutations in the NOTCH pathway regulator cause left ventricular noncompaction cardiomyopathy. Nat. Med. *19*, 193–201. https://doi.org/10.1038/nm.3046.

Maas, R.G.C., Lee, S., Harakalova, M., Snijders Blok, C.J.B., Goodyer, W.R., Hjortnaes, J., Doevendans, P.A.F.M., Van Laake, L.W., van der Velden, J., Asselbergs, F.W., et al. (2021). Massive expansion and cryopreservation of functional human induced pluripotent stem cell-derived cardiomyocytes. STAR Protoc. *2*, 100334. https://doi.org/10.1016/j.xpro.2021.100334.

Menendez-Montes, I., Escobar, B., Palacios, B., Gómez, M.J., Izquierdo-Garcia, J.L., Flores, L., Jiménez-Borreguero, L.J., Aragones, J., Ruiz-Cabello, J., Torres, M., and Martin-Puig, S. (2016). Myocardial VHL-HIF Signaling Controls an Embryonic Metabolic Switch Essential for Cardiac Maturation. Dev. Cell *39*, 724–739. https://doi.org/10.1016/j.devcel.2016.11.012.

Mills, R.J., Parker, B.L., Quaife-Ryan, G.A., Voges, H.K., Needham, E.J., Bornot, A., Ding, M., Andersson, H., Polla, M., Elliott, D.A., et al. (2019). Drug Screening in Human PSC-Cardiac Organoids Identifies Pro-proliferative Compounds Acting via the Mevalonate Pathway. Cell Stem Cell *24*, 895–907.e6. https://doi.org/10.1016/j.stem.2019.03.009.

Mills, R.J., Titmarsh, D.M., Koenig, X., Parker, B.L., Ryall, J.G., Quaife-Ryan, G.A., Voges, H.K., Hodson, M.P., Ferguson, C., Drowley, L., et al. (2017). Functional screening in human cardiac organoids reveals a metabolic mechanism for cardiomyocyte cell cycle arrest. Proc. Natl. Acad. Sci. USA *114*, E8372–E8381. https://doi.org/10.1073/pnas.1707316114.

Miyata, S., Minobe, W., Bristow, M.R., and Leinwand, L.A. (2000). Myosin heavy chain isoform expression in the failing and nonfailing human heart. Circ. Res. *86*, 386–390. https://doi.org/10.1161/01.res.86.4.386.

Nusse, R., and Clevers, H. (2017). Wntβ-Catenin Signaling, Disease, and Emerging Therapeutic Modalities. Cell *169*, 985–999. https://doi.org/10.1016/j.cell.2017.05.016.

Ouwerkerk, W., Belo Pereira, J.P., Maasland, T., Emmens, J.E., Figarska, S.M., Tromp, J., Koekemoer, A.L., Nelson, C.P., Nath, M., Romaine, S.P.R., et al. (2023). Multiomics Analysis Provides Novel Pathways Related to Progression of Heart Failure. J. Am. Coll. Cardiol. *82*, 1921–1931. https://doi.org/10.1016/j.jacc.2023.08.053.

Pettinato, A.M., Yoo, D., VanOudenhove, J., Chen, Y.S., Cohn, R., Ladha, F.A., Yang, X.L., Thakar, K., Romano, R., Legere, N., et al. (2021). Sarcomere function activates a p53-dependent DNA damage response that promotes polyploidization and limits cell engraftment. Cell Rep. *35*, 109088. https://doi.org/10.1016/j.celrep.2021.109088.

Quaife-Ryan, G.A., Mills, R.J., Lavers, G., Voges, H.K., Vivien, C.J., Elliott, D.A., Ramialison, M., Hudson, J.E., and Porrello, E.R. (2020). β-Catenin drives distinct transcriptional networks in proliferative and nonproliferative cardiomyocytes. Development *147*, dev193417. https://doi.org/10.1242/dev.193417.

Reiser, P.J., Portman, M.A., Ning, X.H., and Schomisch Moravec, C. (2001). Human cardiac myosin heavy chain isoforms in fetal and failing adult atria and ventricles. Am. J. Physiol. Heart Circ. Physiol. *280*, H1814–H1820. https://doi.org/10.1152/ajpheart.2001.280.4.H1814.

Schade, D., Drowley, L., Wang, Q.D., Plowright, A.T., and Greber, B. (2022). Phenotypic screen identifies FOXO inhibitor to counteract maturation and promote expansion of human iPS cell-derived cardiomyocytes. Bioorg. Med. Chem. *65*, 116782. https://doi.org/10.1016/j.bmc.2022.116782.

Sharma, A., Zhang, Y., Buikema, J.W., Serpooshan, V., Chirikian, O., Kosaric, N., Churko, J.M., Dzilic, E., Shieh, A., Burridge, P.W., et al. (2018). Stage-specific Effects of Bioactive Lipids on Human iPSC Cardiac Differentiation and Cardiomyocyte Proliferation. Sci. Rep. *8*, 6618. https://doi.org/10.1038/s41598-018-24954-3.

Titmarsh, D.M., Glass, N.R., Mills, R.J., Hidalgo, A., Wolvetang, E.J., Porrello, E.R., Hudson, J.E., and Cooper-White, J.J. (2016). Induction of Human iPSC-Derived Cardiomyocyte Proliferation Revealed by Combinatorial Screening in High Density Microbioreactor Arrays. Sci. Rep. *6*, 24637. https://doi.org/10.1038/srep24637.

Uosaki, H., Magadum, A., Seo, K., Fukushima, H., Takeuchi, A., Nakagawa, Y., Moyes, K.W., Narazaki, G., Kuwahara, K., Laflamme, M., et al. (2013). Identification of chemicals inducing cardiomyocyte proliferation in developmental stage-specific manner with pluripotent stem cells. Circ. Cardiovasc. Genet. *6*, 624–633. https://doi.org/10.1161/CIRCGENETICS.113.000330.

von Gise, A., Lin, Z., Schlegelmilch, K., Honor, L.B., Pan, G.M., Buck, J.N., Ma, Q., Ishiwata, T., Zhou, B., Camargo, F.D., and Pu, W.T. (2012). YAP1, the nuclear target of Hippo signaling, stimulates heart growth through cardiomyocyte proliferation but not hypertrophy. Proc. Natl. Acad. Sci. USA *109*, 2394–2399. https://doi.org/10.1073/pnas.1116136109.

Xin, M., Kim, Y., Sutherland, L.B., Murakami, M., Qi, X., McAnally, J., Porrello, E.R., Mahmoud, A.I., Tan, W., Shelton, J.M., et al. (2013). Hippo pathway effector Yap promotes cardiac regeneration. Proc. Natl. Acad. Sci. USA *110*, 13839–13844. https://doi.org/10.1073/pnas.1313192110.

Ye, B., Hou, N., Xiao, L., Xu, Y., Boyer, J., Xu, H., and Li, F. (2015). APC controls asymmetric Wnt/β-catenin signaling and cardiomyocyte proliferation gradient in the heart. J. Mol. Cell. Cardiol. *89*, 287–296. https://doi.org/10.1016/j.yjmcc.2015.10.018.

Yuan, Q., Maas, R.G.C., Brouwer, E.C.J., Pei, J., Blok, C.S., Popovic, M.A., Paauw, N.J., Bovenschen, N., Hjortnaes, J., Harakalova, M., et al. (2022). Sarcomere Disassembly and Transfection Efficiency in Proliferating Human iPSC-Derived Cardiomyocytes. J. Cardiovasc. Dev. Dis. *9*, 43. https://doi.org/10.3390/jcdd9020043.

Stem Cell Reports, Volume *20*

Supplemental Information

Glycogen synthase kinase-3 inhibition and insulin enhance proliferation and inhibit maturation of human iPSC-derived cardiomyocytes via TCF and FOXO signaling

Qianliang Yuan, Devin Verbueken, Rafeeh Dinani, Rosa Kim, Eric Schoger, Chloé D. Morsink, Shamim Amiri Simkooei, Luuk J.M. Kemna, Jesper Hjortnaes, Diederik W.D. Kuster, Reinier A. Boon, Laura Cecilia Zelarayan, Jolanda van der Velden, and Jan W. Buikema

Supplemental Information

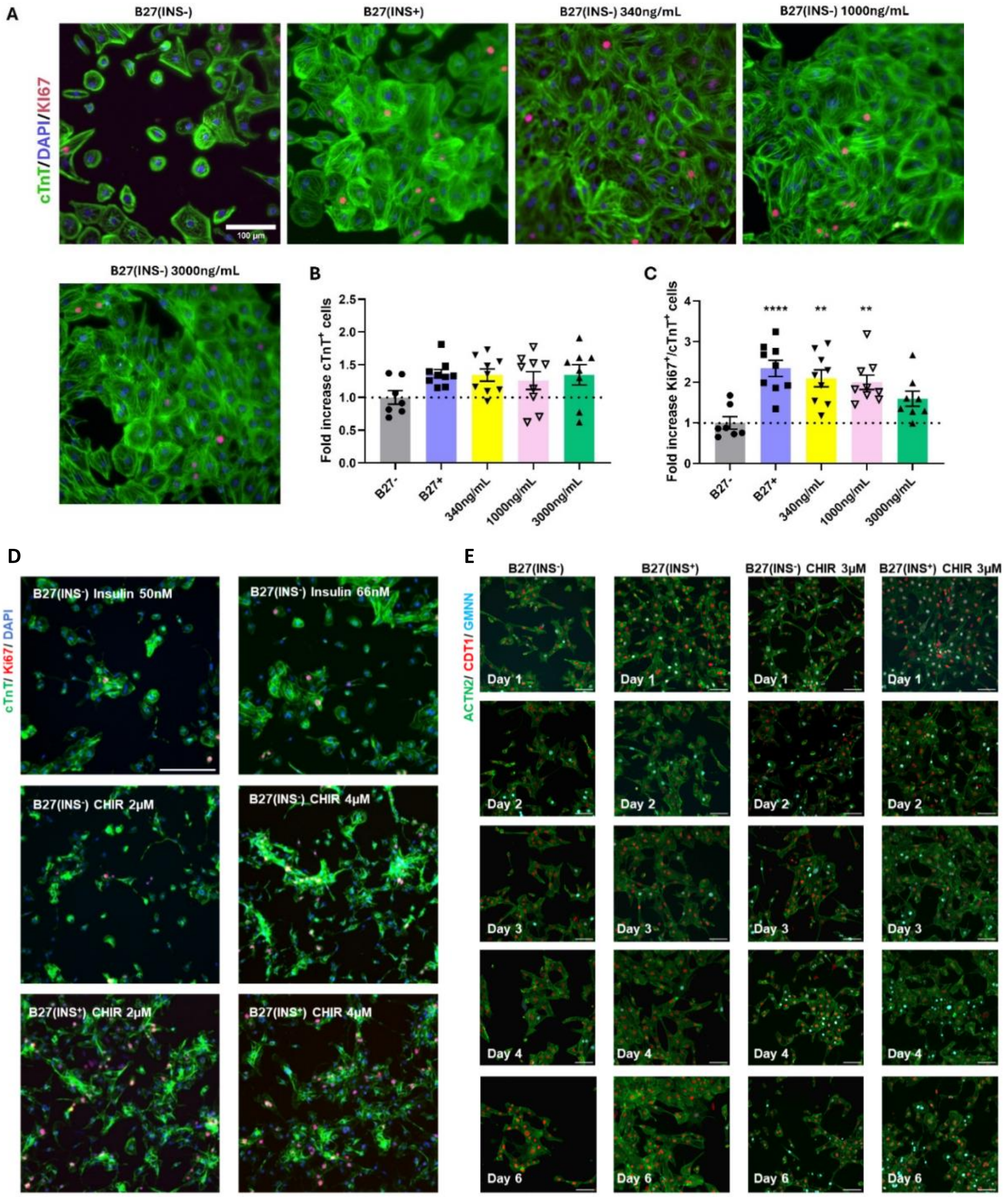

Figure S1: Insulin concentration screening for proliferative response in immature hiPSC-CMs and monitoring of cell cycle activity. (**A**) Representative immunofluorescence images of immature SCVI-273 hiPSC-CMs that were cultured over 7 days exposed to an exponential array of Insulin concentration. Cells were stained for cTnT (green), Ki67 (red), and nuclei (DAPI, blue). (**B**) Quantification of cTnT$^+$ hiPSC-CMs cultured in B27(INS+), B27(INS-) + 340ng/mL (~58nM), B27(INS-) + 1000ng/mL (~170nM), B27(INS-) + 3000ng/mL (~500nM), with fold increases relative to B27(INS-) condition. (**C**) Quantification of Ki67$^+$ cells among cTnT$^+$ hiPSC-CMs for all conditions of (B) with fold increased relative to B27(INS-). Data are presented as mean +/- SEM. Experiment was performed in

triplicate, every data point represents analysis of one image. After confirmation of normality using the D'Agostino & Pearson test, statistical significance was tested using one-way ANOVA followed by Dunn's test for multiple comparisons relative to B27(INS-). $^{**}p < 0.01$, $^{****}p < 0.0001$. (**D)** Representative immunofluorescence images of hiPSC-CMs exposed to the gradient concentrations of stimuli, Insulin and/or CHIR in 7 days. The cells were stained for cTnT (green), Ki67 (red), and nuclei (DAPI, blue). Scale bar=200 µm. (**E**) Live cell imaging of TC1133-ACTN2-Citrine-FUCCI hiPSC-CMs (ACTN2+, green) in the absence or presence of CHIR and Insulin. G1-cardiomyocytes are labeled with mCherry (CDT1, red) and S/G2-cardiomyocytes are labeled with ECFP (GMNN, light blue). Cells were monitored over a time course of 6 days. Scale bar=100 µm.

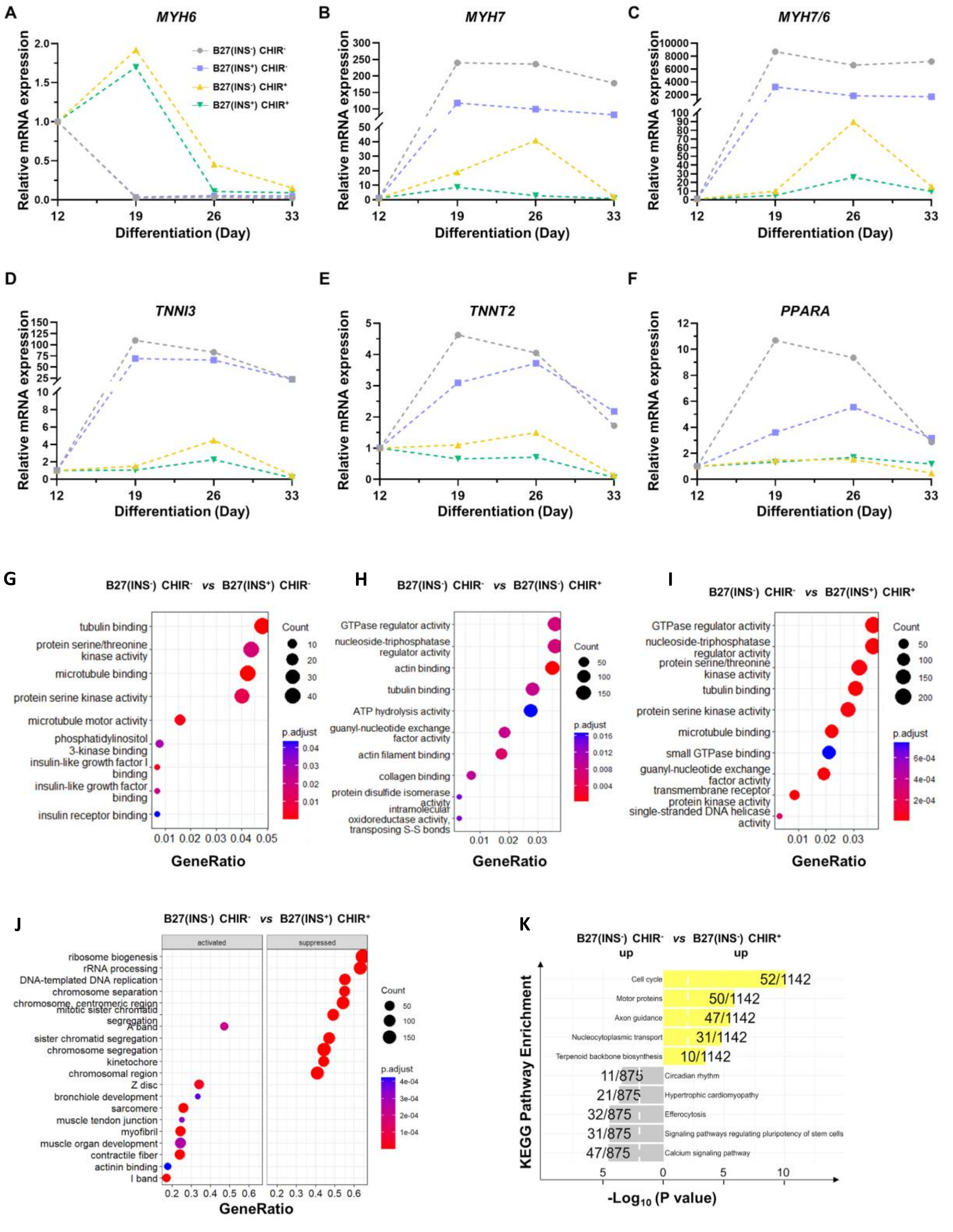

Figure S2. Expression of selected genes in hiPSC-CMs during prolonged treatment with Insulin/AKT and CHIR99021/Wnt activators (Related to Figure 2) and GO terms and KEGG pathway analysis.

(A-F) Gene expression, including *MYH6* (**A**), *MYH7* (**B**), *MYH7*/*MYH6* (**C**), *TNNI3* (**D**), *TNNT2* (**E**), *PPARA* (**F)**, displays a temporal pattern following treatment with insulin and/or CHIR99021 post-differentiation (Day 12). **(G-I**) Gene Ontology (GO) analysis of DEGs identified molecular functions

enriched in the designed conditions in hiPSC-CMs culture B27(INS$^+$) CHIR$^-$ (**G**), B27(INS$^-$) CHIR$^+$ (**H**), and B27(INS$^+$) CHIR$^+$ (**I**), comparing to B27(INS$^-$) CHIR$^-$, respectively. **GeneRatio**, the ratio of the number of DEGs to the total number of genes in the gene set. (**J**) GSEA-GO analysis of B27(INS$^-$) CHIR$^-$ versus B27(INS$^+$) CHIR$^+$. (**K**) Top KEGG pathways enrichment analysis between B27(INS$^-$) CHIR$^-$ and B27(INS$^-$) CHIR$^+$.

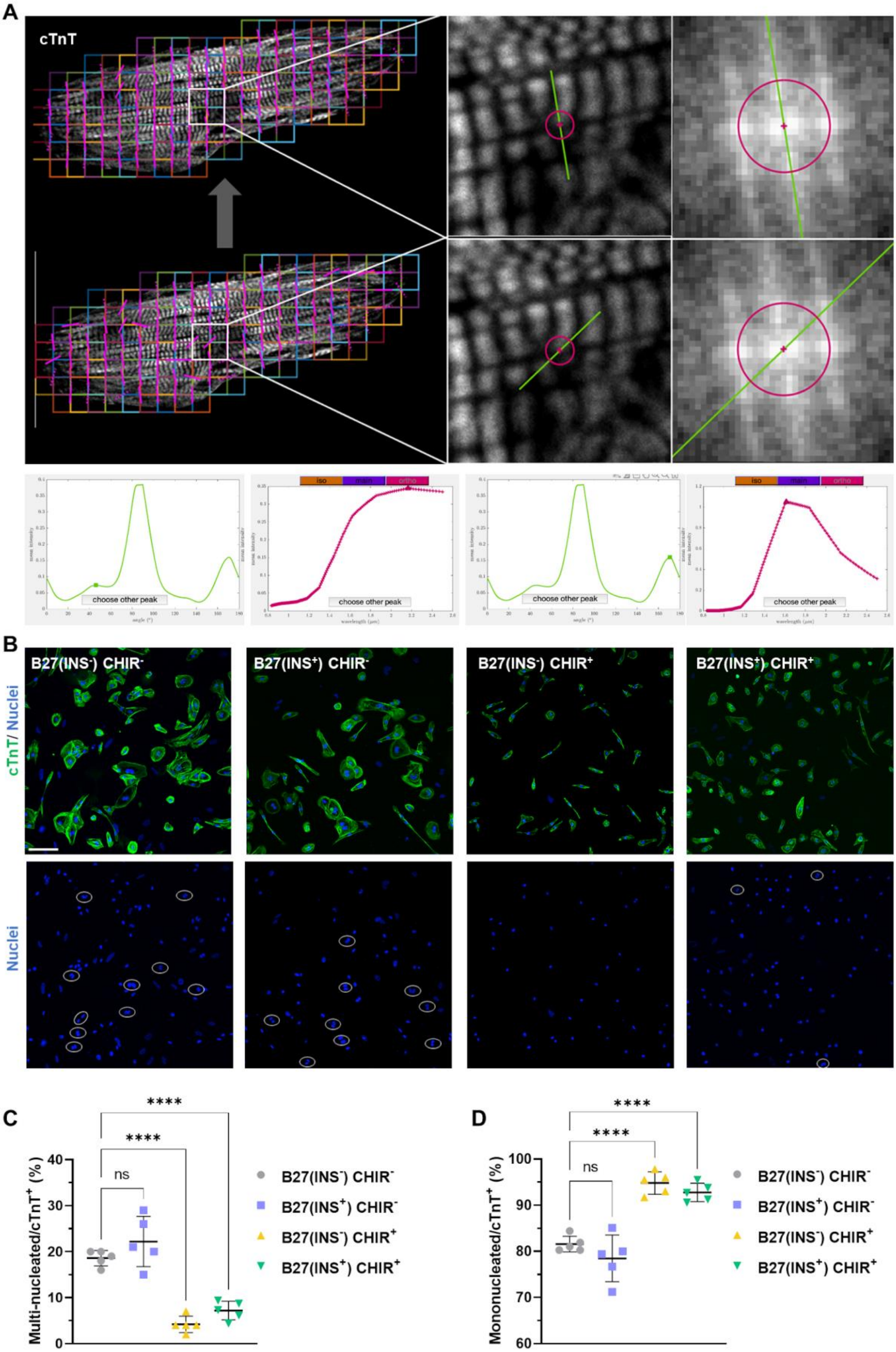
A
cTnT
iso
choose other peak
B
B27(INS-) CHIR-
B27(INS+) CHIR-
B27(INS-) CHIR+
B27(INS+) CHIR+
cTnT/ Nuclei
Nuclei
C
Multi-nucleated/cTnT+ (%)
ns
****
0
10
20
30
40
D
Mononucleated/cTnT+ (%)
60
70
80
90
100
B27(INS-) CHIR-
B27(INS+) CHIR-
B27(INS-) CHIR+
B27(INS+) CHIR+

Figure S3. Insulin and CHIR99021 prompted the generation of mononuclear hiPSC-CMs. (**A**) Workflow for sarcomere quantification using MATLAB. (**B**) Representative immunofluorescence images of hiPSC-CMs show cTnT staining in red, with nuclei labeled in blue. Multi-nucleated cells are highlighted by circles as indicated. Scale bar=100 µm. (**C**) Quantification of the percentage of multi-nucleated hiPSC-CMs among total cTnT-positive hiPSC-CMs. (**D**) Quantification of the percentage of mononucleated hiPSC-CMs among total cTnT-positive hiPSC-CMs. Data are presented as mean ± SD and compared to B27(INS$^-$) CHIR$^-$ condition. N=5 independent cultures from SCVI-273 cell line. Not significant (ns), $p > 0.05$, ***$p < 0.001$, and ****$p < 0.0001$.

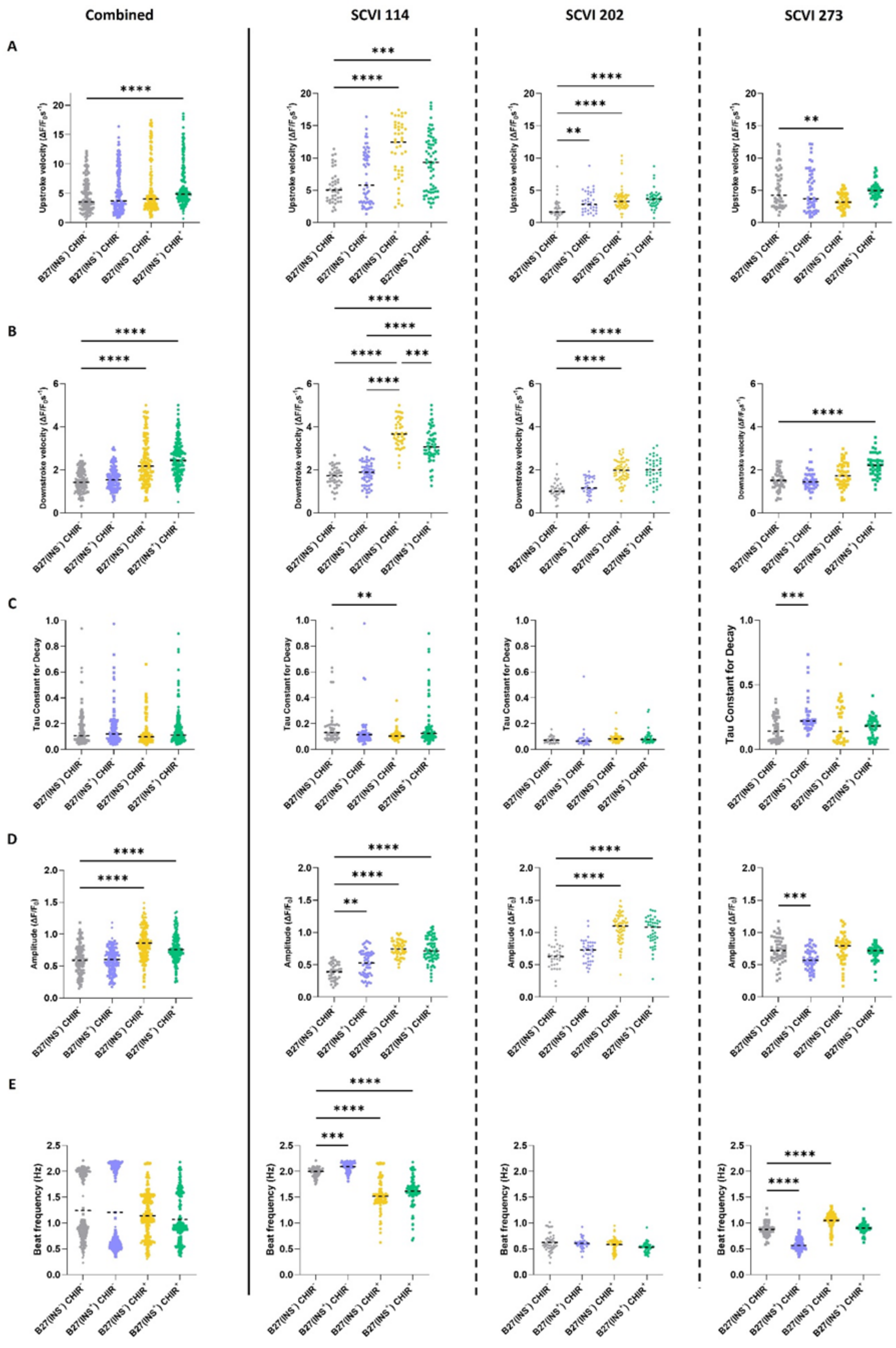

Combined
SCVI 114
SCVI 202
SCVI 273
A
B
C
D
E
Upstroke velocity (ΔF/F0s-1)
Downstroke velocity (ΔF/F0s-1)
Tau Constant for Decay
Amplitude (ΔF/F0)
Beat frequency (Hz)
B27(INS-) CHIR-
B27(INS+) CHIR-
B27(INS-) CHIR+
B27(INS+) CHIR+

Figure S4. Results of calcium measurements subdivided per cell line.
Scatterplots of upstroke velocity (**A**), downstroke velocity (**B**), tau constant for decay (**C**), calcium amplitude (**D**), and beating frequency (**E**) per cell line. Dotted line represents the median (for E the mean is displayed) and every dot is a measurement of one beating area (●= SCVI-114, ♦= SCVI-202, ■= SCVI-273). Comparisons are performed by Kruskal-Wallis test followed by Dunn's multiple comparisons test with B27(INS$^-$) CHIR$^-$ as the reference condition. Not significant, $p > 0.05$, *$p < 0.05$, **$p < 0.01$, ****$p < 0.0001$.

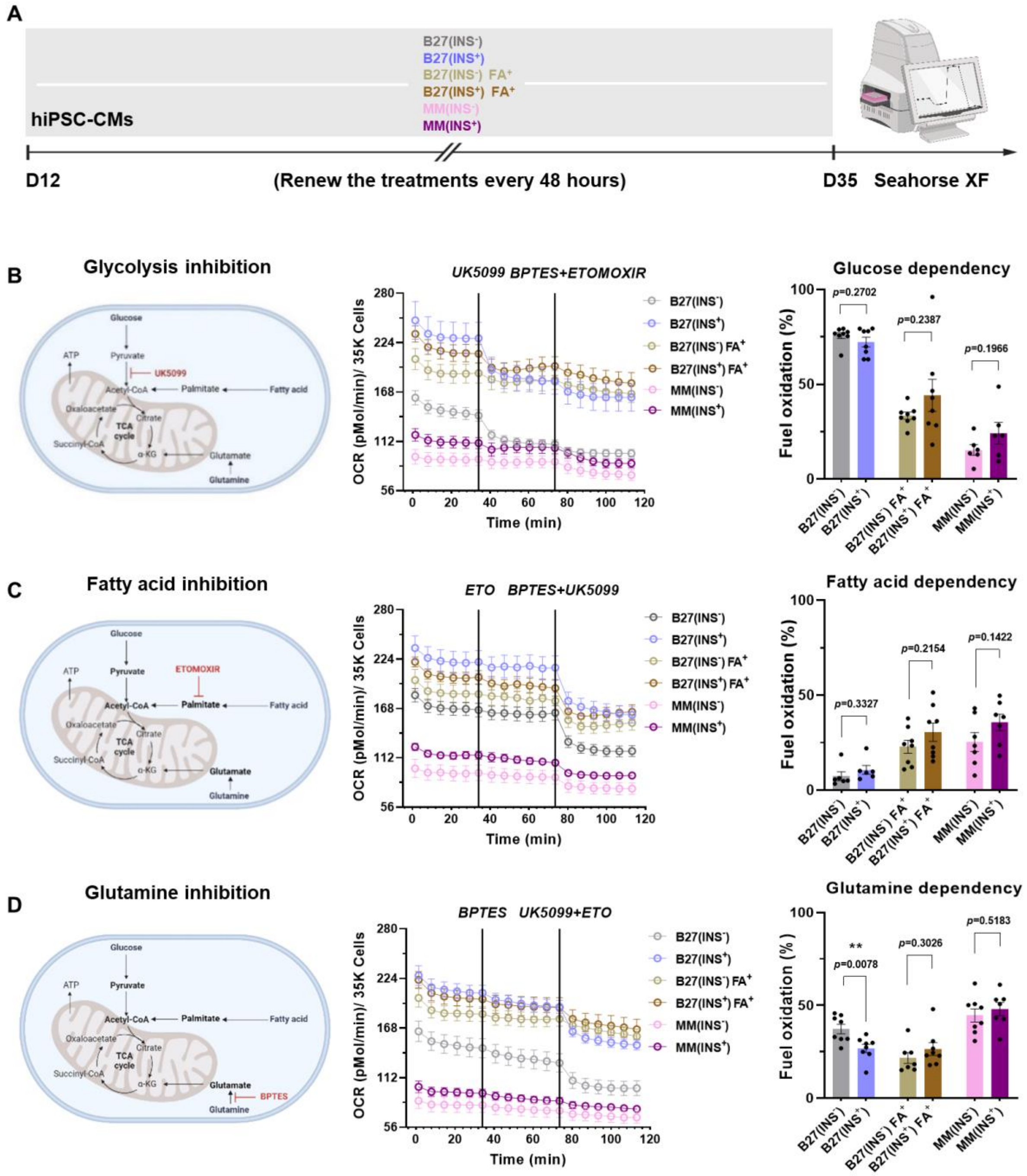

Figure S5. Differential substrate fuel utilization in hiPSC-CMs under defined culture media conditions modulated by insulin. (**A**) Schematic of the experimental timeline for Seahorse XF Mito Fuel-Flex assay involving hiPSC-CMs in culture separately with B27 with insulin (B27(INS^{+})), B27 without insulin (B27(INS^{-})), the addition of free fatty acid to B27(INS^{-}) or B27(INS^{+}), maturation medium (MM) with insulin (MM(INS^{+})) or without insulin (MM(INS^{-})). (**B-D**) Dependency on glucose (**B**), fatty acid (**C**), and glutamine (**D**) oxidation of hiPSC-CMs cultured in the indicated media. Created with BioRender.com. Data are presented as mean ± SEM. N≥ 6 independent cultures from 3 batches of SCVI-273 cell line. Significance assessed with unpaired Student's t-test between the groups, B27(INS^{-}) and B27(INS^{+}), B27(INS^{-}) FA^{+} and B27(INS^{+}) FA^{+}, MM(INS^{-}) and MM(INS^{+}), respectively. $^{**}p < 0.01$, $p > 0.05$, not significant (ns).

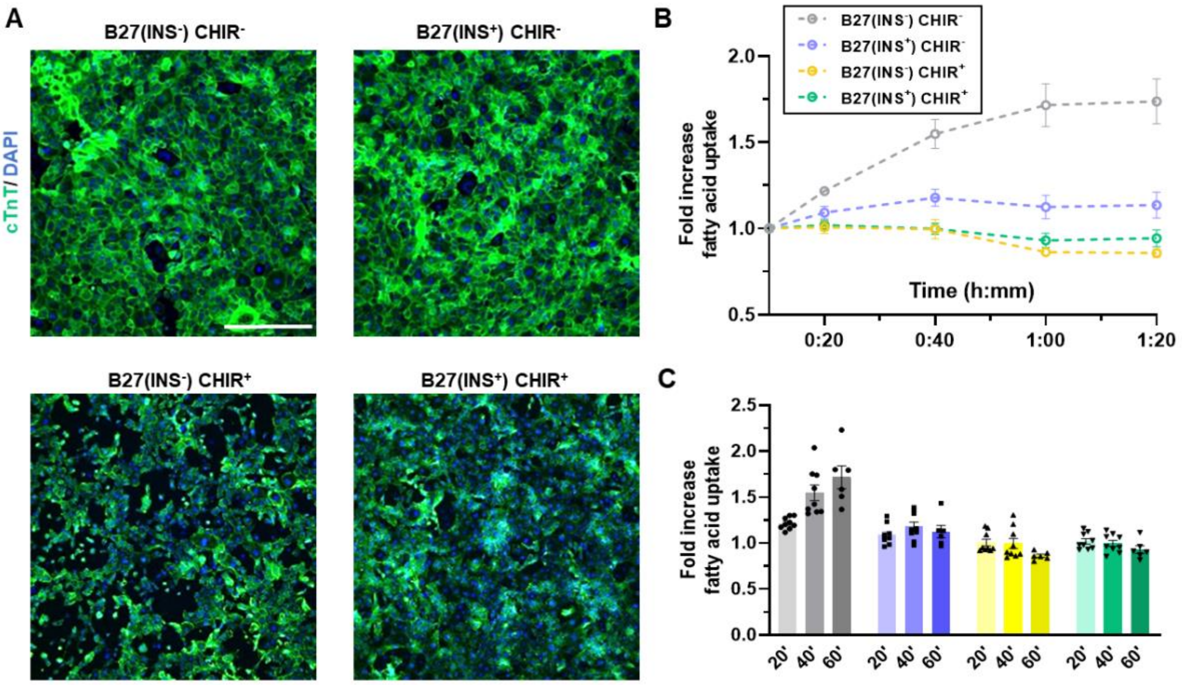

Figure S6. Fatty acid uptake in hiPSC-CMs treated with or without Insulin and/or CHIR99021. (Related to Figure 4). (**A**) Representative immunofluorescence images of hiPSC-CMs cultured under the indicated conditions with cTnT staining (green) and nuclei labeled with DAPI (blue). Scale bar=200 µm. (**B-C**) Fatty acid uptake fold increases measured every 20 minutes for cultured hiPSC-CMs **(B)**. Highlight trends for fatty acid uptake increase in 20, 40, and 60 minutes for individual culture conditions (**C**). Data are normalized to the starting point, and presented as mean ± SEM. N≥ 6 independent cultures from 3 batches of SCVI-273 and SCVI-114 cell lines.

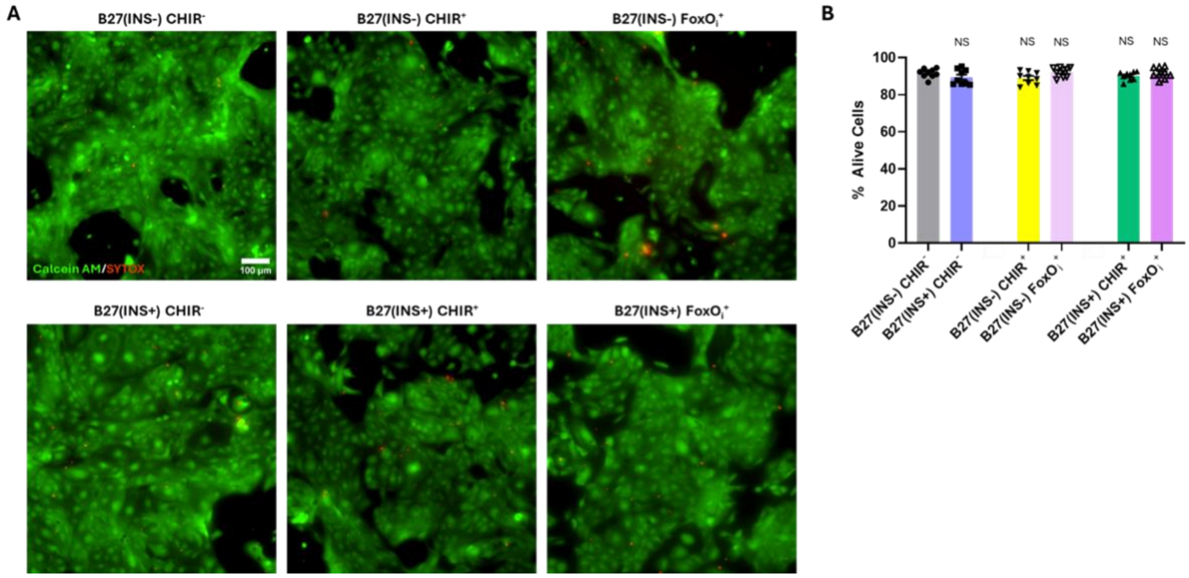

Figure S7. Administration of WNT activator (CHIR99021) and FOXO inhibitor (AS1842856) do not affect cell viability of immature hiPSC-CMs. (**A**) Live/Dead staining of immature SCVI-273 hiPSC-CMs after exposure for 24 hours to the designed culture conditions. Calcein AM indicates alive and viable cells (green), and SYTOX Deep Red staining indicates dead cells (red). (**B**) Quantification of percentage viable hiPSC-CMs per total cells for each condition. Data are presented as mean ± SEM. Experiment was performed in triplicate, every data point represents analysis of one image. After confirmation of normality using the D'Agostino & Pearson test, statistical significance was tested using one-way ANOVA followed by Tukey's multiple comparisons test relative to B27(INS-) CHIR$^-$.

Table S1. Primers

Function	Target	Sequence (5' --> 3')	Length	Tm
Myofibril maturation	*MYH6*	F: CGGTGCTTTTCAACCTCAAGG	21	61.7
		R: GGACTGGTTCTCCCGATCTGT	21	63.0
	MYH7	F: CTTTGCTGTTATTGCAGCCATT	22	60.0
		R: AGATGCCAACTTTCCTGTTGC	21	61.1
	TNNI1	F: CCGGAAGTCGAGAGAAAACCC	21	62.4
		R: TCAATGTCGTATCGCTCCTCA	21	60.7
	TNNI3	F: TTTGACCTTCGAGGCAAGTTT	21	60.1
		R: CCCGGTTTTCCTTCTCGGTG	20	62.8
	TNNT2	F: GGAGGAGTCCAAACCAAAGCC	21	62.9
		R:TCAAAGTCCACTCTCTCTCCATC	23	60.8
	MYL2	F: TTGGGCGAGTGAACGTGAAAA	21	62.7
		R: CCGAACGTAATCAGCCTTCAG	21	60.8
Cellular metabolism	*PPARA*	F: CGGTGACTTATCCTGTGGTCC	21	61.9
		R:CCGCAGATTCTACATTCGATGTT	23	60.7
	PPARG	F: GGGATCAGCTCCGTGGATCT	20	63.0
		R:TGCACTTTGGTACTCTTGAAGTT	23	60.0
	PPARGC1a	F: GCTTTCTGGGTGGACTCAAGT	21	62.0
		R: GAGGGCAATCCGTCTTCATCC	21	62.5
Wnt targets	*CTNNB1*	F: AAAGCGGCTGTTAGTCACTGG	21	62.6
		R: CGAGTCATTGCATACTGTCCAT	22	60.2
	AXIN2	F: CAACACCAGGCGGAACGAA	19	62.8
		R:GCCCAATAAGGAGTGTAAGGACT	23	61.4
	CCND2	F: CTGTCTCTGATCCGCAAGCAT	21	62.2
		R:GGTGGGTACATGGCAAACTTAAA	23	61.0
	LEF1	F: AGAACACCCCGATGACGGA	19	68.0
		R: GGCATCATTATGTACCCGGAAT	22	65.4
Housekeeping gene	*GAPDH*	F: GGAGCGAGATCCCTCCAAAAT	21	61.6
		R: GGCTGTTGTCATACTTCTCATGG	23	60.9

Table S2. Composition of metabolic substrates in media

Media<br><br>Compositions	RPMI 1640 basal medium		
	B27 (+/- Insulin)	B27 (+/- Insulin)+ FA	MM (+/- Insulin)
**Glucose**	11 mM	11 mM	3 mM
**L-Glutamine**	2 mM	2 mM	4 mM
**Fatty Acids**	Not disclosed	5 mg/mL	5 mg/mL
**L-carnitine**	Exists, but the conc. not disclosed	2 mM	2 mM

Table S3. Top 50 differentially expressed genes for the indicated comparisons

B27(INS-) CHIR- vs. B27(INS+) CHIR-

Downregulated genes				
	Gene	*ENSEMBL*	*Log2FoldChange*	*Padj*
1	PDK4	ENSG00000004799	-2,86	1,47E-04
2	ENSG00000231170	ENSG00000231170	-2,20	1,88E-03
3	CCBE1	ENSG00000183287	-2,20	2,17E-04
4	PLSCR5	ENSG00000231213	-2,00	2,79E-06
5	GFRA1	ENSG00000151892	-1,49	5,89E-06
6	DLK1	ENSG00000185559	-1,45	1,64E-27
7	UNC5A	ENSG00000113763	-1,44	2,41E-02
8	PCARE	ENSG00000179270	-1,29	1,05E-10
9	LINC01122	ENSG00000233723	-1,28	3,89E-02
10	TDRD6	ENSG00000180113	-1,26	4,35E-02
11	MT1F	ENSG00000198417	-1,26	4,56E-02
12	PMEL	ENSG00000185664	-1,25	4,92E-12
13	POLN	ENSG00000130997	-1,23	3,98E-02
14	ENSG00000287109	ENSG00000287109	-1,22	2,42E-03
15	NDRG1	ENSG00000104419	-1,18	1,22E-08
16	FSTL5	ENSG00000168843	-1,18	1,84E-02
17	KLF5	ENSG00000102554	-1,17	3,95E-03
18	PIK3IP1	ENSG00000100100	-1,14	8,32E-12
19	C11orf21	ENSG00000110665	-1,13	5,04E-05
20	NEGR1	ENSG00000172260	-1,11	6,59E-03
21	CNTN5	ENSG00000149972	-1,10	2,20E-03
22	NKAIN2	ENSG00000188580	-1,07	3,27E-02
23	PTCHD1	ENSG00000165186	-1,04	5,25E-05
24	SCN11A	ENSG00000168356	-1,01	1,36E-02
25	IRS2	ENSG00000185950	-1,01	2,80E-14
26	ACOXL-AS1	ENSG00000204581	-0,99	2,23E-02
27	LRRN1	ENSG00000175928	-0,99	1,58E-03
28	KCNJ10	ENSG00000177807	-0,97	1,13E-02
29	SLC22A3	ENSG00000146477	-0,97	9,12E-03
30	LSAMP	ENSG00000185565	-0,96	7,00E-17
31	GPR35	ENSG00000178623	-0,95	4,87E-02
32	CNR1	ENSG00000118432	-0,94	4,61E-02
33	PKD1L2	ENSG00000166473	-0,94	1,19E-06
34	KLF3-AS1	ENSG00000231160	-0,92	4,00E-04
35	ACSM3	ENSG00000005187	-0,92	2,05E-02
36	PELI2	ENSG00000139946	-0,92	5,37E-08
37	ENSG00000287315	ENSG00000287315	-0,91	4,94E-05
38	ENSG00000261888	ENSG00000261888	-0,90	4,52E-02
39	ITGB8	ENSG00000105855	-0,89	1,99E-03
40	STAC	ENSG00000144681	-0,89	1,56E-02
41	GRM8	ENSG00000179603	-0,89	4,33E-02
42	ADGRG2	ENSG00000173698	-0,88	1,79E-03
43	UNC80	ENSG00000144406	-0,86	8,59E-03
44	ITGA9	ENSG00000144668	-0,86	3,82E-06
45	ENSG00000271155	ENSG00000271155	-0,86	6,69E-03
46	LINC00842	ENSG00000285294	-0,86	1,28E-02
47	SEMA3E	ENSG00000170381	-0,84	1,04E-04
48	SLITRK5	ENSG00000165300	-0,83	9,72E-03
49	AMPH	ENSG00000078053	-0,82	4,55E-02
50	WNT2B	ENSG00000134245	-0,82	4,52E-02

Upregulated genes				
	Gene	*ENSEMBL*	*Log2FoldChange*	*Padj*
1	ELF3	ENSG00000163435	2,20	1,28E-04
2	ENG	ENSG00000106991	2,16	4,99E-02
3	FUT1	ENSG00000174951	1,82	1,56E-02
4	SERPINA1	ENSG00000197249	1,77	3,34E-03
5	NEFH	ENSG00000100285	1,77	4,04E-06
6	DNAI3	ENSG00000162643	1,75	8,75E-10
7	CCDC68	ENSG00000166510	1,75	2,88E-03
8	ODAM	ENSG00000109205	1,74	2,49E-03
9	LINC01182	ENSG00000250634	1,68	1,81E-02
10	STC1	ENSG00000159167	1,65	1,03E-04
11	AHNAK2	ENSG00000185567	1,64	8,35E-36
12	GJA5	ENSG00000265107	1,59	2,19E-10
13	RELT	ENSG00000054967	1,50	7,58E-06
14	TMEM63C	ENSG00000165548	1,44	1,32E-04
15	GPR3	ENSG00000181773	1,43	2,11E-02
16	CDH1	ENSG00000039068	1,40	1,52E-02
17	DYNLT5	ENSG00000152760	1,40	4,90E-04
18	SPOCK2	ENSG00000107742	1,39	1,65E-04
19	DUSP13	ENSG00000079393	1,36	2,41E-03
20	GRIN2B	ENSG00000273079	1,34	2,10E-03
21	SPAG4	ENSG00000061656	1,30	6,71E-03
22	DPF1	ENSG00000011332	1,29	7,90E-04
23	MOG	ENSG00000204655	1,24	9,79E-04
24	CNIH2	ENSG00000174871	1,22	1,63E-02
25	MMP1	ENSG00000196611	1,22	3,51E-02
26	HMGB1P1	ENSG00000124097	1,20	4,51E-02
27	HNRNPA1P27	ENSG00000233680	1,20	2,68E-02
28	MAT1A	ENSG00000151224	1,20	2,99E-02
29	XKRX	ENSG00000182489	1,19	1,11E-02
30	TRPV4	ENSG00000111199	1,19	7,50E-03
31	LAMB3	ENSG00000196878	1,19	3,15E-02
32	SNX10	ENSG00000086300	1,17	2,85E-04
33	RDM1	ENSG00000278023	1,17	4,78E-02
34	SLC6A20	ENSG00000163817	1,14	6,09E-06
35	ARHGAP45	ENSG00000180448	1,14	2,90E-07
36	CYTOR	ENSG00000222041	1,12	1,55E-02
37	SERTM2	ENSG00000260802	1,11	2,36E-07
38	NEIL3	ENSG00000109674	1,10	8,80E-05
39	DBNDD1	ENSG00000003249	1,10	1,84E-03
40	RNFT2	ENSG00000135119	1,10	3,96E-06
41	TUBB4A	ENSG00000104833	1,09	4,91E-03
42	ATP8B3	ENSG00000130270	1,09	2,06E-02
43	PLEKHG1	ENSG00000120278	1,08	6,48E-05
44	DDIT4	ENSG00000168209	1,08	2,93E-19
45	SREBF1	ENSG00000072310	1,06	5,74E-13
46	TM6SF1	ENSG00000136404	1,06	1,77E-02
47	L1TD1	ENSG00000240563	1,06	1,57E-02
48	GLT1D1	ENSG00000151948	1,04	2,23E-02
49	LINC00880	ENSG00000243629	1,03	1,07E-02
50	P2RX1	ENSG00000108405	1,02	4,52E-02

B27(INS-) CHIR- vs. B27(INS-) CHIR+

Downregulated genes				
	Gene	*ENSEMBL*	*Log2FoldChange*	*Padj*
1	XIRP2	ENSG00000163092	-4,59	5,28E-23
2	LMOD3	ENSG00000163380	-4,41	1,19E-57
3	PDE1A	ENSG00000115252	-4,41	2,56E-12
4	GPR22	ENSG00000172209	-4,31	4,22E-32
5	TCIM	ENSG00000176907	-4,16	1,58E-04
6	PSKH2	ENSG00000147613	-3,87	6,53E-05
7	BMP10	ENSG00000163217	-3,86	2,75E-37
8	ENSG00000230623	ENSG00000230623	-3,83	4,20E-04
9	NRAP	ENSG00000197893	-3,76	4,48E-04
10	SDAD1P1	ENSG00000228451	-3,61	5,33E-22
11	ASB11	ENSG00000165192	-3,53	1,66E-45
12	LINC01034	ENSG00000224933	-3,50	2,46E-04
13	ANKRD2	ENSG00000165887	-3,46	1,27E-03
14	SLC6A20	ENSG00000163817	-3,45	2,66E-12
15	C11orf21	ENSG00000110665	-3,44	7,99E-30
16	OR51E1	ENSG00000180785	-3,37	1,88E-03
17	ENSG00000249261	ENSG00000249261	-3,36	9,35E-04
18	NT5E	ENSG00000135318	-3,34	7,74E-05
19	KCNA5	ENSG00000130037	-3,34	5,16E-26
20	DMGDH	ENSG00000132837	-3,30	1,31E-05
21	KRT7	ENSG00000135480	-3,27	2,27E-11
22	PTGS2	ENSG00000073756	-3,24	2,55E-05
23	DLK1	ENSG00000185559	-3,21	5,14E-77
24	ENSG00000254202	ENSG00000254202	-3,21	1,40E-06
25	KLHL38	ENSG00000175946	-3,19	3,60E-48
26	KCNA7	ENSG00000104848	-3,16	6,33E-18
27	RGS8	ENSG00000135824	-3,15	1,57E-03
28	CFAP61	ENSG00000089101	-3,15	1,71E-06
29	ENSG00000254966	ENSG00000254966	-3,12	1,54E-04
30	HHATL	ENSG00000010282	-3,08	2,26E-09
31	ACTG2	ENSG00000163017	-3,08	1,38E-26
32	SLITRK6	ENSG00000184564	-3,07	3,73E-07
33	RASSF10	ENSG00000189431	-3,05	6,23E-04
34	SLC16A14	ENSG00000163053	-3,04	4,75E-29
35	ACTN3	ENSG00000248746	-3,03	2,91E-05
36	PPP1R3A	ENSG00000154415	-3,03	3,30E-06
37	ACOXL-AS1	ENSG00000204581	-2,98	1,79E-10
38	ENSG00000231170	ENSG00000231170	-2,96	7,05E-06
39	TTR	ENSG00000118271	-2,94	2,17E-04
40	SOX15	ENSG00000129194	-2,93	1,15E-08
41	NPIPB2	ENSG00000234719	-2,91	1,98E-06
42	SCT	ENSG00000070031	-2,90	4,16E-03
43	ENG	ENSG00000106991	-2,88	5,30E-06
44	CYSLTR1	ENSG00000173198	-2,84	3,40E-08
45	KLRD1	ENSG00000134539	-2,83	1,41E-04
46	LINC01449	ENSG00000224017	-2,83	8,99E-08
47	ATP1B4	ENSG00000101892	-2,80	1,22E-04
48	CACNA1S	ENSG00000081248	-2,77	4,94E-04
49	CTXND1	ENSG00000259417	-2,76	1,62E-13
50	DSCAML1	ENSG00000177103	-2,75	8,41E-05

Upregulated genes				
	Gene	*ENSEMBL*	*Log2FoldChange*	*Padj*
1	TMEM132C	ENSG00000181234	7,79	1,14E-29
2	GLDC	ENSG00000178445	6,44	7,26E-93
3	RSPO2	ENSG00000147655	5,01	4,54E-30
4	ISM1	ENSG00000101230	4,94	9,99E-08
5	SP5	ENSG00000204335	4,68	8,45E-32
6	LURAP1L	ENSG00000153714	4,58	1,20E-75
7	TAGLN3	ENSG00000144834	4,40	2,44E-06
8	UNC5D	ENSG00000156687	4,30	3,15E-130
9	LHX4	ENSG00000121454	4,10	3,34E-13
10	LINC02395	ENSG00000257771	4,06	1,32E-08
11	PDYN	ENSG00000101327	4,04	2,12E-11
12	CES1	ENSG00000198848	3,81	2,34E-05
13	ADAMTSL2	ENSG00000197859	3,72	5,60E-25
14	ENSG00000213981	ENSG00000213981	3,64	2,50E-04
15	ENSG00000278514	ENSG00000278514	3,59	3,24E-13
16	GRIN2B	ENSG00000273079	3,56	5,10E-33
17	LINC00269	ENSG00000215162	3,50	1,16E-04
18	ENSG00000285637	ENSG00000285637	3,44	3,97E-04
19	NEGR1	ENSG00000172260	3,42	5,22E-83
20	DCN	ENSG00000011465	3,42	2,86E-28
21	DIRAS2	ENSG00000165023	3,30	2,12E-52
22	RGCC	ENSG00000102760	3,27	2,18E-13
23	CMTM5	ENSG00000166091	3,21	8,53E-24
24	ENSG00000236924	ENSG00000236924	3,16	6,46E-05
25	OTULINL	ENSG00000145569	3,13	3,03E-28
26	PLPPR4	ENSG00000117600	3,08	8,35E-44
27	KCNG1	ENSG00000026559	3,06	4,56E-76
28	MEGF10	ENSG00000145794	3,05	5,00E-29
29	LYPD1	ENSG00000150551	3,00	1,71E-39
30	NDP	ENSG00000124479	3,00	1,07E-05
31	PALM3	ENSG00000187867	2,94	1,70E-09
32	NKD1	ENSG00000140807	2,91	4,93E-50
33	SLC25A48	ENSG00000145832	2,88	8,69E-23
34	ECEL1	ENSG00000171551	2,85	1,11E-35
35	TOX3	ENSG00000103460	2,85	6,33E-09
36	CD74	ENSG00000019582	2,83	5,93E-18
37	ENSG00000283235	ENSG00000283235	2,81	7,19E-03
38	DISC1FP1	ENSG00000261645	2,79	5,62E-05
39	WNT2	ENSG00000105989	2,78	1,10E-12
40	MYO10	ENSG00000145555	2,76	9,76E-49
41	CA8	ENSG00000178538	2,75	5,58E-59
42	ROBO2	ENSG00000185008	2,75	6,42E-21
43	HOXD9	ENSG00000128709	2,73	3,35E-04
44	HOXD8	ENSG00000175879	2,72	3,12E-08
45	ENSG00000280061	ENSG00000280061	2,71	1,88E-19
46	RP1	ENSG00000104237	2,64	1,62E-03
47	PALMD	ENSG00000099260	2,64	9,15E-10
48	AJAP1	ENSG00000196581	2,63	3,34E-07
49	RPRM	ENSG00000177519	2,63	7,97E-05
50	NRXN3	ENSG00000021645	2,58	2,33E-31

B27(INS-) CHIR- vs. B27(INS+) CHIR+

Downregulated genes				
	Gene	*ENSEMBL*	*Log2FoldChange*	*Padj*
1	PDK4	ENSG00000004799	-4,64	5,18E-23
2	XIRP2	ENSG00000163092	-4,56	6,13E-30
3	BMP10	ENSG00000163217	-4,55	1,80E-59
4	KCNA5	ENSG00000130037	-4,40	1,03E-39
5	DNAAF3-AS1	ENSG00000267577	-4,32	1,09E-16
6	LINC00702	ENSG00000233117	-4,29	2,70E-19
7	SEMA5B	ENSG00000082684	-4,21	1,02E-55
8	ENSG00000231170	ENSG00000231170	-4,20	3,73E-07
9	ATP1B4	ENSG00000101892	-4,18	1,60E-05
10	LMOD3	ENSG00000163380	-4,07	1,21E-77
11	SOX15	ENSG00000129194	-3,93	1,98E-13
12	KRT80	ENSG00000167767	-3,93	1,04E-14
13	KLHL38	ENSG00000175946	-3,89	6,99E-88
14	GPR22	ENSG00000172209	-3,85	9,54E-42
15	ENSG00000286198	ENSG00000286198	-3,81	9,35E-12
16	CFAP61	ENSG00000089101	-3,80	5,83E-12
17	C11orf21	ENSG00000110665	-3,79	7,77E-45
18	SLITRK6	ENSG00000184564	-3,79	2,59E-10
19	PKD1L2	ENSG00000166473	-3,79	5,81E-48
20	ENSG00000275216	ENSG00000275216	-3,78	3,91E-06
21	ANKRD34C	ENSG00000235711	-3,78	3,10E-05
22	TCIM	ENSG00000176907	-3,76	1,25E-05
23	NT5E	ENSG00000135318	-3,70	2,67E-06
24	DLK1	ENSG00000185559	-3,69	9,03E-122
25	VIPR2	ENSG00000106018	-3,62	5,45E-25
26	ASB11	ENSG00000165192	-3,62	2,27E-57
27	LINC01449	ENSG00000224017	-3,61	5,37E-10
28	NRAP	ENSG00000197893	-3,58	1,43E-04
29	KCNA7	ENSG00000104848	-3,55	7,62E-26
30	SLC5A7	ENSG00000115665	-3,53	1,88E-15
31	SYNPR	ENSG00000163630	-3,49	9,09E-24
32	ACTG2	ENSG00000163017	-3,48	6,52E-36
33	ENSG00000285774	ENSG00000285774	-3,47	4,66E-04
34	HOPX	ENSG00000171476	-3,39	5,12E-88
35	TNS1-AS1	ENSG00000223923	-3,39	3,33E-61
36	ACOXL-AS1	ENSG00000204581	-3,37	1,73E-12
37	IL31RA	ENSG00000164509	-3,36	1,36E-07
38	CTXND1	ENSG00000259417	-3,34	3,78E-22
39	LINC02175	ENSG00000262155	-3,32	9,17E-45
40	SLC6A5	ENSG00000165970	-3,31	3,70E-06
41	DSCAML1	ENSG00000177103	-3,30	3,99E-06
42	SDAD1P1	ENSG00000228451	-3,30	1,54E-24
43	KRT7	ENSG00000135480	-3,29	2,35E-14
44	ANKRD2	ENSG00000165887	-3,28	5,50E-04
45	C5orf46	ENSG00000178776	-3,22	4,64E-08
46	KLHDC7B	ENSG00000130487	-3,21	1,11E-03
47	TCP11L2	ENSG00000166046	-3,19	1,84E-74
48	ENSG00000283128	ENSG00000283128	-3,19	9,18E-07
49	FGF1	ENSG00000113578	-3,18	1,07E-194
50	ENSG00000279619	ENSG00000279619	-3,17	4,74E-06

Upregulated genes				
	Gene	*ENSEMBL*	*Log2FoldChange*	*Padj*
1	TMEM132C	ENSG00000181234	8,31	4,28E-34
2	GLDC	ENSG00000178445	6,69	2,18E-112
3	RSPO2	ENSG00000147655	5,48	6,63E-39
4	PDYN	ENSG00000101327	5,23	4,70E-21
5	TAGLN3	ENSG00000144834	5,22	1,31E-08
6	LINC00269	ENSG00000215162	5,14	1,53E-08
7	SP5	ENSG00000204335	5,08	9,53E-37
8	ISM1	ENSG00000101230	5,08	2,69E-08
9	CES1	ENSG00000198848	4,90	6,28E-08
10	GRIN2B	ENSG00000273079	4,76	1,05E-115
11	UNC5D	ENSG00000156687	4,61	1,31E-237
12	LURAP1L	ENSG00000153714	4,57	8,99E-86
13	LINC02395	ENSG00000257771	4,55	2,65E-12
14	NEFH	ENSG00000100285	4,45	3,52E-89
15	LHX4	ENSG00000121454	4,34	2,10E-15
16	WNT2	ENSG00000105989	4,32	1,10E-57
17	USP18	ENSG00000184979	4,25	3,41E-06
18	ENSG00000278514	ENSG00000278514	4,12	1,35E-18
19	ADAMTSL2	ENSG00000197859	4,10	1,50E-38
20	SPTA1	ENSG00000163554	4,00	1,49E-05
21	ENPP2	ENSG00000136960	3,93	1,57E-15
22	OTULINL	ENSG00000145569	3,90	2,14E-52
23	KCNG1	ENSG00000026559	3,79	1,39E-187
24	NKD1	ENSG00000140807	3,77	1,26E-74
25	CMTM5	ENSG00000166091	3,76	8,60E-46
26	DRD2	ENSG00000149295	3,68	1,55E-09
27	TLR3	ENSG00000164342	3,57	2,03E-06
28	PALM3	ENSG00000187867	3,57	3,48E-17
29	LINC02200	ENSG00000250358	3,57	3,09E-04
30	PLPPR4	ENSG00000117600	3,57	6,31E-96
31	CD74	ENSG00000019582	3,55	1,46E-36
32	CLEC1B	ENSG00000165682	3,53	1,83E-04
33	MEGF10	ENSG00000145794	3,51	1,09E-63
34	LINC01351	ENSG00000237457	3,47	1,69E-04
35	LINC01315	ENSG00000229891	3,42	4,55E-05
36	ENSG00000285637	ENSG00000285637	3,40	3,39E-04
37	DCN	ENSG00000011465	3,39	4,20E-38
38	ENSG00000213981	ENSG00000213981	3,37	5,60E-04
39	DPF1	ENSG00000011332	3,36	1,09E-65
40	HOXD8	ENSG00000175879	3,34	2,73E-13
41	NRXN3	ENSG00000021645	3,34	4,87E-80
42	ENSG00000236924	ENSG00000236924	3,30	1,77E-05
43	ENSG00000287311	ENSG00000287311	3,30	3,76E-04
44	TMEM100	ENSG00000166292	3,28	2,60E-09
45	SLC35D3	ENSG00000182747	3,27	1,98E-18
46	CA8	ENSG00000178538	3,22	6,28E-125
47	SLCO4A1	ENSG00000101187	3,22	6,76E-06
48	DIRAS2	ENSG00000165023	3,22	4,66E-29
49	ECEL1	ENSG00000171551	3,21	1,41E-82
50	GJA5	ENSG00000265107	3,20	3,54E-92

STAR Methods

Key resource table

REAGENT or RESOURCE	SOURCE	IDENTIFIER
**Antibodies**		
Cardiac Troponin T	Abcam	# Ab8295
Ki67	Abcam	# Ab15580
MLC2V	Abcam	# Ab48003
TOMM20	Abcam	# Ab186735
Donkey anti-Rabbit IgG (H+L) Alexa Fluor-488	Invitrogen	# A21206
Donkey anti-Rabbit IgG (H+L) Alexa Fluor-555	Invitrogen	# A31572
Donkey anti-Mouse IgG (H+L) Alexa Fluor-647	Invitrogen	# A31571
**Critical commercial assays**		
PSC Cryopreservation kit	ThermFisher	# A2644601
RNeasy Plus Mini Kit	QIAGEN	# 74134
Cell Line Optimization 4D-Nucleofector™ X Kit	Lonza	# V4XC-9064
Superscript cDNA synthesis kit	ThermFisher	# 11904018
iTaq SYBR Green Supermix	Bio-Rad	# 1725120
Free Fatty Acid Uptake Assay Kit	Abcam	# Ab176768
Seahorse XF Cell Mito Stress Test Kit	Agilent	# 103015-100
Seahorse XF Mito Fuel Flex Test Kit	Agilent	# 103260-100
Lipofectamine™ 3000 Transfection Kit	Invitrogen	# L3000001
Dual-Luciferase® Reporter Assay System	Promega	# E1910
**Biological samples**		
TCF reporter plasmid	Addgene	# M50
**Chemicals, peptides, and recombinant proteins**		
CHIR99021	Selleckchem	# S2924

C59	Selleckchem	# S737
Insulin solution	Sigma	# I9278
Matrigel® Growth Factor Reduced	Corning	# 354230
RPMI 1640	ThermoFisher	# 11875119
RPMI NO GLUCOSE	ThermoFisher	# 11879020
B27 supplement plus insulin	GIBCO	# 317504044
B27 supplement minus insulin	GIBCO	# A1895601
Essential 8 medium	GIBCO	# A1517001
DMEM/F12	GIBCO	# 21331020
DMEM, no glucose	GIBCO	# 11966025
KnockOut™ Serum Replacement	GIBCO	# 10828010
DAPI	ThermFisher	# 62248
DPBS (1X)	GIBCO	# 14190144
Bovine Serum Albumin	Sigma-Aldrich	# A7906
Hoechst 33342	ThermoFisher	# 62249
EDTA	ThermoFisher	# 15575020
TRYPLE 10X SEL	ThermoFisher	# A1217701
Revitacell Supplement (100x)	ThermoFisher	# A2644501
Rock inhibitor Y-27632	StemCell Technologies	# 72304
DMSO	Merck-Sigma	# D2650
Paraformaldehyde, 4% in PBS	ThermFisher	# 61899-AK
2-Phospho-L-ascorbic acid trisodium salt	Sigma-Aldrich	# 66170-10-3
Albumin	Sigma-Aldrich	# 70024-90-7
AlbuMAX™ I Lipid-Rich BSA	ThermFisher	# 11020021
Glucose	Sigma-Aldrich	# G7021
L-lactate	Sigma-Aldrich	# 71718
L-carnitine	Sigma-Aldrich	# C0283
Ascorbic acid	Sigma-Aldrich	# A8960
1x NEAA	ThermFisher	# 11140

Penicillin/Streptomycin	ThermoFisher	# 15140122
Fura-2, AM	Thermo Fisher	# F1221
Lipofectamine	Invitrogen	# L3000001
Luciferase substrate	Promega	# E1910
**Deposited data**		
Bulk RNA sequencing		GSE278598
**Experimental model: Cell lines**		
hiPSC line	Stanford Biobank	SCVI-273
hiPSC line	Stanford Biobank	SCVI-114
hiPSC line	Stanford Biobank	SCVI-111
hiPSC line	Stanford Biobank	SCVI-202
hiPSC line	UMG	TC1133-ACTN2-Citrine
**Oligonucleotide**		
**Primers**	This paper	Table S1
**Software and algorithms**		
GraphPad Prism 9	GraphPad Software	http://survey-smiles.com
Fiji-ImageJ	Shindelin et al., 2012	https://imagej.net/software/fiji/
Python 3.10.13	Python Software Foundation	https://www.python.org/
MATLAB_R2022b	MathWorks	https://www.mathworks.com/products/matlab.html
Ionwizard software	CytoCypher	https://www.ionoptix.com/products/software/ionwizard-core-and-analysis/
QuPath 0.5.0	Bankhead et al., 2017	https://github.com/qupath/qupath
BioRender	Science Suite Inc.	https://www.biorender.com/

R v4.0.2	R Core Team, 2021	https://www.r-project.org/
clusterProfiler	Yu et al., 2012	https://bioconductor.org/packages<br>/release/bioc/html/clusterProfiler.html
DESeq2	Love et al., 2014	https://bioconductor.org/packages<br>/release/bioc/html/DESeq2.html
PCAtools	Blighe and Lun, 2020	https://bioconductor.org/packages<br>/release/bioc/html/PCAtools.html
ggplot2 package	R. A. M. Villanueva 2019	https://ggplot2.tidyverse.org

Resource availability

Lead contact

For additional information or requests regarding resources and reagents, please contact the corresponding author, Jan W. Buikema, j.w.buikema@amsterdamumc.nl

Materials availability

Please direct any additional information or requests for materials to the lead contact.

METHOD DETAILS

Immunofluorescence staining and confocal microscopy imaging

HiPSC-CMs were seeded at a density of approximately $6x10^4$ cells/well into a µ-Plate 8-well chamber or $2x10^4$ cells/well into a 96-well plate in this study. After experimental treatments, the cells were gently washed with PBS (1X) and fixed using 4% PFA for 10 minutes at room temperature. Following fixation, cells were permeabilized with 0.25% PBS-Triton for 10 minutes and then blocked using 3% BSA for 1 hour. Subsequently, the cells were incubated overnight at 4°C with primary antibodies (see key resource table for details). On the following day, the cells were incubated with corresponding fluorescently labeled secondary antibodies for

1 hour at room temperature. DAPI staining was applied for visualizing nuclear DNA. Imaging was conducted using either an AXR confocal microscope or an Eclipse Ti2 inverted microscope system (Nikon). Optionally, Z-stacks were acquired with a slice thickness of 0.22 µm under the 40X/0.95 NA objective. Subsequent image analysis and quantification were performed using NIS Elements software or ImageJ software.

For FUCCI CM live cell imaging: CQ1 Confocal Imaging Cytometer (Yokogawa) was used with a 20X/0.8 NA objective. Cells seeding density was $5x10^5$ cells/well for a 6-well cell culture plate. Images were quantified by Python and ImageJ.

Quantitative reverse transcription PCR (RT-qPCR)

HiPSC-CMs were harvested and lysed using TRIzol™ reagent (Invitrogen). Total RNA extraction was conducted according to the manufacturer's instructions using the miRNeasy Mini kit (Qiagen). RNA concentration was measured by loading 1µL of extracted RNA samples on NanoDrop (Thermo Fisher Scientific). A total of 1000 ng of RNA was used for cDNA synthesis by using iScript cDNA synthesis kit (BioRad). Subsequently, the cDNA was diluted to a concentration of 5 ng/µL for subsequent RT-qPCR analysis. The RT-qPCR experiment was carried out using SYBR Green Supermix (Bio-Rad) on a Bio-Rad CFX96 device. For gene expression analysis, normalization was performed using the housekeeping gene glyceraldehyde-3-phosphate dehydrogenase (GAPDH) and calculated by the $2^{-\Delta\Delta Ct}$ method (Livak & Schmittgen, 2001).

Contractility measurements and calcium transient analysis

All contractility and calcium imaging experiments were performed on spontaneously beating hiPSC-CMs. To this end, d12 hiPSC-CMs were cultured under the following conditions: B27(INS$^-$) CHIR$^-$, B27(INS$^+$) CHIR$^-$, B27(INS$^-$) CHIR$^+$, and B27(INS$^+$) INS$^+$ for 4 days. Subsequently, the cells were seeded at a concentration of $4x10^5$ cells/well on a 24-well black 14 mm plate (IBIDI, 82421) coated with Matrigel (Corning) for another 3 days, with continuous treatment under the defined conditions as described above. Contractility and calcium transient measurements followed previously described methods (Dinani et al., 2023), with details modified and adapted for this study. Contractility measurements were conducted using the Cytocypher Multicell High Throughput System (Cytocypher BV (Amsterdam, The Netherlands), IonOptix Corporation (Westwood, MA, United States) when the cells were grown and treated under the aforementioned conditions. Spontaneously beating areas from monolayer cells were randomly selected and measured for 10 seconds per well across 12 regions for a total of 10 minutes. The data were collected using Transient Analysis Tool software (Cytocypher BV), and analyzed according to the following calculations: Contraction time = Time to peak – Time to peak 10. Relaxation time = Time to baseline 80 – Time to peak.

%Shortening = Peak height/ Baseline. For calcium transient measurements, Tyrode solution with a 1 mM Calcium concentration was prepared by thawing a 10 mL 10x Tyrode stock. To this stock, 80 mL MilliQ water, 55 mg sodium pyruvate, and 100 µL $CaCl_2$ were added. The pH was adjusted to 7.4 at 37°C. The medium in the wells was replaced with warm Tyrode solution at 37°C and incubated for 15 minutes. Fura-2-AM was diluted to a concentration of 2 µM by adding 2 µL per milliliter. Subsequently, the Tyrode solution was replaced with Fura-2-AM-containing Tyrode and incubated for 15 minutes. This was followed by replacement with normal Tyrode and an additional incubation for 5 minutes. Finally, the Tyrode was refreshed once, and the plate was measured in the Cytocypher (IonOptix) for calcium measurements. Spontaneously beating areas from monolayer cells were randomly selected and measured for 10 seconds per well across 12 regions, totaling 10 minutes. Data were collected using Transient Analysis Tool software (Cytocypher BV) and analyzed for ΔF/F0.

Seahorse XF Mito stress assay

To evaluate the effect of the CHIR99021 and insulin on mitochondrial respiration, Seahorse XF Mito-stress assay was performed according to manufacturer's instructions. HiPSC-CMs were independently cultured for 7 days in B27(INS$^-$) CHIR$^-$, B27(INS$^+$) CHIR$^-$, B27(INS$^-$) CHIR$^+$, and B27(INS$^-$) CHIR$^+$. 3-5 days prior to the experiment cells were replated into the Seahorse XF96-well cell culture microplate (Agilent) coated with Matrigel (Corning). On the day of the experiment, media were replaced by RPMI 1640 basal medium supplemented with 2 mM glutamine, 10 mM glucose, and 1 mM sodium pyruvate. To normalize the oxygen consumption rate (OCR) values to the actual cell number, cells were incubated with Hoechst (Thermo Fisher Scientific) for live cell imaging, and cell number was counted by a cell analyzer. Subsequently, mitochondrial stress test conditions were assessed according to manufacturer's instructions of the Seahorse XF Cell Mito Stress Test Kit (Agilent) using Seahorse XF96 platform by injecting the following inhibitors in sequence: Oligomycin (2.5 µM), FCCP (carbonyl cyanide-4 (trifluoromethoxy) phenylhydrazone, 2 µM), and Rotenone/antimycin A (2.5 µM). Parameters, including basal respiration, ATP-linked respiration, proton leak, and respiratory capacity, were calculated based on the changes in the OCR upon injection of the beforehand mentioned inhibitors.

Seahorse XF Mito Fuel Flex assay

To assess the effect of insulin on the substrate metabolism dependency of hiPSC-CMs in different defined culture media, Fuel-Flex assay was performed according to manufacturer's instructions. HiPSC-CMs were independently cultured in B27(INS$^-$) and B27(INS$^+$), B27(INS$^-$) FA$^+$ and B27(INS$^+$) FA$^+$, maturation medium minus insulin (MM(INS$^-$)) and maturation medium plus insulin (MM(INS$^+$)) until day 35 post-differentiation. 3-5 days prior to the experiment, cells were replated into the Seahorse XFe96 Cell Culture Microplates (Agilent)

coated with Matrigel (Corning). On the day of the experiment, media were replaced by RPMI 1640 basal medium (Thermo Fisher Scientific) supplemented with 2 mM glutamine, 10 mM glucose, and 1 mM sodium pyruvate. To normalize the OCR values to the actual cell number, cells were incubated with Hoechst (Thermo Fisher Scientific) for live cell imaging. Subsequently, cell number was counted by a cell analyzer shortly before the seahorse experiments. Three main substrate oxidation pathways were analyzed according to manufacturer's instructions from Seahorse XF Mito Fuel Flex Test Kit (Agilent). The inhibitors from the kit were injected sequentially for the test: A) UK5099, a pyruvate carrier inhibitor, was used to assess the mitochondrial glycolysis dependency, B) Etomoxir, an inhibitor of long chain fatty acid metabolism, and C) BPTES, an inhibitor of glutamine oxidation pathway. Mitochondrial dependency to each substrate was calculated using the following formula: Substrate fuel dependency = ((baseline OCR – target inhibitor OCR)/ (baseline OCR - all inhibitors OCR))*100.

Fatty acid uptake assay

Four days prior to the assay, the Matrigel-coated 96-well black plate with clear bottom was seeded with $6x10^4$ hiPSC-CMs. The assessment of fatty acid was carried out using the Free Fatty Acid Uptake Assay Kit (Abcam) following the manufacturer's instruction. Briefly, after being washed thrice with serum-free media, the cells were pre-incubated in serum-free medium for 2 hours. A baseline measurement was conducted before introducing the fluorescent fatty acid mixture, followed by measurements every 10 minutes after the addition using a microplate fluorescence reader (Greiner Bio-One) at 485/528 nm performed in Spectramax ID3 (Molecular Devices).

Bulk RNA-seq analysis

D12 hiPSC-CMs were cultured, respectively, under the defined conditions, including B27(INS$^-$) CHIR$^-$, B27(INS$^+$) CHIR$^-$, B27(INS$^-$) CHIR$^+$, and B27(INS$^-$) CHIR$^+$, for 3 days. Subsequently, total RNA was extracted following the described method as above. Library preparations were carried out using the NEBNext® Ultra RNA Library Prep Kit for Illumina® (New England Biolabs), and the prepared libraries underwent sequencing on a Hiseq 4000 platform (Novogene). The RNA-seq reads were aligned to the human reference genome hg19. Differential expression analysis of genes (DEGs) between groups (adjusted p value < 0.01) was conducted by DESeq using RStudio. Functional enrichment analysis was performed by Bioconductor packages using RStudio.

Luciferase-based LEF/TCF activity analysis

β-Catenin/TCF activity was evaluated using the luciferase-based LEF/TCF reporter, TOPFlash plasmid (Addgene). For early-stage cells, D12 hiPSC-CMs were cultured in RPMI 1640 basal medium-based conditions: B27(INS$^-$) CHIR$^-$, B27(INS$^+$) CHIR$^-$, B27(INS$^-$) CHIR$^+$, and B27(INS$^-$) CHIR$^+$ for 4 days (D16). For late-stage cells, D12 hiPSC-CMs were cultured in RPMI 1640 basal media plus insulin until D40, cells were switched to the conditions: B27(INS$^-$) CHIR$^-$, B27(INS$^+$) CHIR$^-$, B27(INS$^-$) CHIR$^+$, and B27(INS$^-$) CHIR$^+$ for 4 days (D44). The D16 and D44 cells were, respectively, replated into 96-well clear black bottom plates (Greiner Bio-One) with a cell density 1×10^5 cells/well. The following day, transfection was performed using Lipofectamine™ 3000 Transfection Reagent (Invitrogen) according to the manufacturer's protocol, with the Renilla luciferase gene as an internal control. After 36 h, cells were lysed in standard 1X lysis buffer (Promega) for 30 min at room temperature, and the cell lysates were subjected for both firefly and Renilla luciferase activity using the Dual-Luciferase Reporter assay kit (Promega) following the manufacturer's instructions. Fluorescence was measured using SoftMax® Pro (Molecular Devices), Renilla luciferase was used as a control reporter for normalization. LEF/TCF activity from each culture condition was calculated and finally presented relative to B27(INS$^-$) CHIR$^-$.
